# Association Between Hypoxia‐Inducible Factor‐1α and Neurological Diseases: A Bidirectional Two‐Sample Mendelian Randomization Analysis

**DOI:** 10.1002/brb3.70398

**Published:** 2025-02-28

**Authors:** Jing Liang, Xiaoyan Du, Mengfei Wang, Hongqin Zheng, Yang Sun, Yi Lin

**Affiliations:** ^1^ Department of Neurology and Institute of Neurology of First Affiliated Hospital, Institute of Neuroscience, and Fujian Key Laboratory of Molecular Neurology Fujian Medical University Fuzhou Fujian China

**Keywords:** causal association, HIF‐1α, Mendelian randomization, neurological diseases

## Abstract

**Background:**

Previous studies have suggested that hypoxia‐inducible factor 1‐α (HIF‐1α) exerted multiple effects on different central nervous system disorders. However, it is still uncertain whether plasma HIF‐1α can be a causal indicator for the relevant diseases. This study aimed to test the causality relationship between plasma HIF‐1α and neurological diseases, including cerebrovascular diseases, migraines, and neurodegenerative diseases with a Mendelian randomization (MR) method.

**Methods:**

Single‐nucleotide polymorphisms (SNPs) genetically representing plasma HIF‐1α were screened as instrumental variables (IVs). Summary‐level data for neurological disorder from genome‐wide association studies (GWAS) were identified as outcomes. The causal effects between the IVs and outcomes were determined via the major analysis of inverse‐variance‐weighted (IVW) method. The reverse causal direction was also performed to investigate the possibility of reverse causation.

**Results:**

The findings revealed that plasma HIF‐1α was identified to be genetically associated with cardioembolic stroke (CES) (OR = 0.885; 95% confidence interval [CI] = 0.796–0.985, *p* = 0.026), migraine (OR = 0.941, 95% CI = 0.888–0.998, *p* = 0.041), and drug‐induced migraine without aura (MOA) (OR = 0.586, 95% CI = 0.375–0.916, *p* = 0.019). There was no association identified in plasma HIF‐1α with subarachnoid hemorrhage (SAH), other stroke and migraine subtype, and neurodegenerative disorders. The reverse‐MR analysis revealed that the above‐stated neurological diseases did not have a causal effect on plasma HIF‐1α levels. Sensitivity and validation analyses support that the above results are stable.

**Conclusions:**

Our research indicated that plasma HIF‐1α may have a causal effect on the risk of CES, migraine and drug‐induced MOA, providing new insights for those disease prevention and therapeutic approaches.

## Graphical abstract

A Mendelian randomized study was used to investigate the existence of a causal relationship between plasma HIF‐1α and neurological diseases. Genetically predicted plasma HIF‐1α was negatively associated with the risk of CES, migraine and drug‐induced migraine without aura.

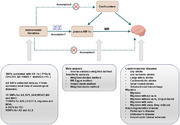

## 1 Introduction

Neurological disorders are increasingly recognized as debilitating diseases with a relatively high lifetime prevalence, posing a significant challenge for nations with aging populations (GBD 2021 Nervous System Disorders Collaborators 2024; Wyss‐Coray 2016). Most neurological diseases remain essentially difficult to treat because of the complex underlying biology and long prodromal periods. Screening for biomarkers associated with disease progression enhances dynamic disease monitoring and facilitates advanced targeted treatment, improving the timing of diagnosis and prevention of neurological diseases (Aslam et al. 2023; Wilson et al. 2023).

The hypoxia‐inducible factor 1‐α (HIF‐1α), a vital mediator of oxygen homeostasis, plays a crucial role in maintaining stability in the brain, which has the highest oxygen demand (Ransohoff 2016; Sanmarco et al. 2023). The expression of HIF‐1α remains stable and low under physiological conditions, but higher expression can be triggered by hypoxia and metabolites (Tannahill et al. 2013; Colgan et al. 2020). HIF‐1α plays an adaptive protective role in ischemic stroke (IS) but can also be destructive, depending on the degree, location, and duration of ischemia (Pan et al. 2021; Jin et al. 2022; Jiang et al. 2024). Elevated serum HIF‐1α levels were discovered in subarachnoid hemorrhage (SAH) and were independently associated with poor clinical outcomes after SAH (Cai et al. 2022). HIF‐1α plays a vital role in promoting the sensitization of migraine by regulating the expression of transient receptor potential melastatin 2 (Ma et al. 2024). HIF‐1α contributes to the formation and accumulation of Aβ peptides, which are considered activators of the initiation and progression of Alzheimer's disease (AD) (Alexander et al. 2022). However, some studies suggest that HIF‐1α inhibits the progression of AD by reducing the activation of glial cells, reactive oxygen species (ROS) production, inflammation, and related processes (Lin et al. 2024; Cirovic et al. 2023). In vitro studies simulating Parkinson's disease (PD) have shown that inducing the expression of HIF‐1α can promote the expression of its downstream genes, including erythropoietin and vascular endothelial growth factor (VEGF), and inhibit neuronal damage (Feng et al. 2014). However, knocking out HIF‐1α in microglia hindered the formation of innate immune memory and suppressed neuroinflammation in MPTP‐intoxicated mice, resulting in neuroprotective effects (Dong et al. 2024). Upregulation of HIF‐1α stability has been shown to suppress the gliosis, apoptosis, and degeneration of motor neurons and myofibers in an in vivo amyotrophic lateral sclerosis (ALS) model (Nomura et al. 2019). However, whether a causal relationship exists between plasma HIF‐1α levels and neurological diseases remains unclear. Therefore, more evidence is needed to determine whether HIF‐1α can serve as a candidate for the prevention and treatment of the aforementioned neurological diseases.

Confounders and potential reverse causality frequently affect the reported findings. Mendelian randomization (MR) is an advanced method for investigating the causal relationships between patient characteristics and clinical manifestations of diseases using genetic variants as instrumental variables (IVs) (Verduijn et al. 2010; Smith and Ebrahim 2003). The principle of random allocation of alleles is the key advantage of MR, as it minimizes confounding that may lead to bias in observational studies. This study employs a bidirectional MR approach to investigate the causal relationship between the levels of plasma HIF‐1α and the risk of neurological disorders, including IS and its subtypes, SAH, migraines and their subtypes, PD, AD, and ALS.

## 2 Methods

### 2.1 Study Design

**FIGURE 1:**
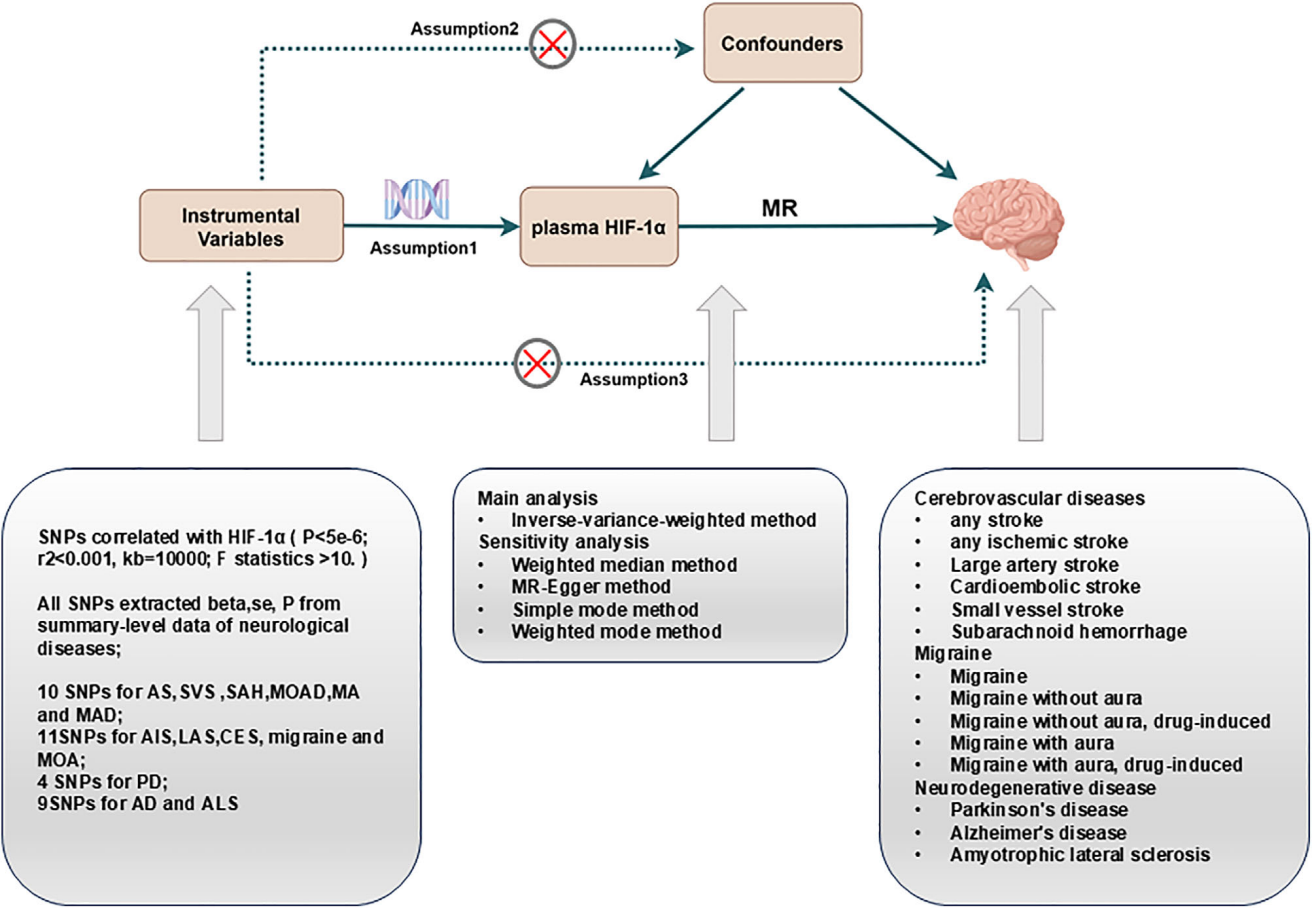
Framework of study design. Assumption 1: Instrumental variables are strongly correlated with exposure. Assumption 2: Instrumental variables are not associated with confounding factors. Assumption 3: Instrumental variables affect outcomes only through exposure. AD, Alzheimer's disease; AIS, any ischemic stroke; ALS, amyotrophic lateral sclerosis; AS, any stroke; CES, cardioembolic stroke; LAS, large artery stroke; MA, migraine with aura; MAD, migraine with aura, drug‐induced; MOA, migraine without aura; MOAD, migraine without aura, drug‐induced; PD, Parkinson's disease; SAH, subarachnoid hemorrhage; SNP, single nucleotide polymorphism; SVS, small vessel stroke.

The general structure of our MR framework is illustrated in Figure 1. As mentioned above, these neurological diseases place a significant physical and financial burden on patients, and it is important to find new and effective biomarkers to predict the risk of diseases. Plasma HIF‐1α was chosen as the potential biomarker due to its potential role in systemic inflammation and its ability to reflect hypoxic and inflammatory conditions, which are known to contribute to the pathogenesis of neurological diseases. IVs representing plasma HIF‐1α levels were selected from genome‐wide association studies (GWAS). To ensure the validity of causal inferences, three key assumptions were made: (1) IVs are strongly associated with exposure; (2) IVs are not associated with confounding factors; and (3) IVs affect outcomes only through exposure. Reverse MR analysis was performed to exclude reverse causal relationships, specifically whether the propensity for these neurological diseases influences plasma HIF‐1α‐related characteristics. This study followed the STROBE‐MR guidelines (Strengthening the Reporting of Observational Studies in Epidemiology using Mendelian randomization) (Skrivankova et al. 2021). The GWAS data used in this research are publicly available, and approval by the corresponding ethics review committee was previously obtained; therefore, additional ethical approval was not required.

### 2.2 GWAS Data Sources

Genetic variants related to plasma HIF‐1α were obtained from comprehensive GWASs in the INTERVAL study, which included 3301 individuals (Sun et al. 2018). The INTERVAL study population consisted of healthy blood donors aged 18–65 years, with an equal distribution of males and females (50% each). The participants were predominantly of European descent, which minimizes population stratification bias in genetic analyses.

The GWAS genetic profiles associated with IS (406,111 controls and 34,217 cases of European ancestry) were obtained from the MEGASTROKE consortium (Malik et al. 2018), which were further divided by subtypes based on the TOAST (Trial of Org 10,172 in Acute Stroke Treatment) criteria, including any stroke (AS, 406,111 controls and 40,585 cases), any IS (AIS, 406,111 controls and 34,217 cases), large‐artery atherosclerotic stroke (LAS, 406,111 controls and 4373 cases), cardioembolic stroke (CES, 406,111 controls and 7193 cases), and small vessel stroke (SVS, 192,662 controls and 5386 cases). The GWAS summary statistics of SAH (201,230 controls and 1338 cases), migraine (176,107 controls and 8547 cases), and its subtypes, including migraine without aura (MOA, 176,107 controls and 3215 cases), migraine without aura, drug‐induced (MOAD, 376,956 controls and 321 cases), migraine with aura (MA, 1176,107 controls and 3541 cases), and migraine with aura, drug‐induced (MAD, 376,970 controls and 307 cases), were obtained from the FinnGen consortium (Kurki et al. 2023). GWAS summary statistics of multiple sclerosis (MS, 68,374 controls and 47,429 cases) were supported by the IMSGC (International Multiple Sclerosis Genetics Consortium) (GBD 2021 Nervous System Disorders Collaborators 2019). The genetic variations of PD (449,056 controls and 33,674 cases) were derived from the IPDGC (International Parkinson's Disease Genomics Consortium) (Nalls et al. 2019). The IGAP (International Genomics of Alzheimer's Project) provided the genetic variations of AD that were utilized in the study, which included 41,944 controls and 21,982 cases (Kunkle et al. 2019). GWAS statistics of ALS (20,806 and 59,804 controls of European descent) were obtained from a study (Nicolas et al. 2018). Table S1 provides the details of the above information.

### 2.3 Selection of Instrumental SNPs

In the study, single‐nucleotide polymorphisms (SNPs) correlated with plasma HIF‐1α levels were selected as the IVs. Initially, no SNPs were identified at the threshold of *p*<5×10^−8^. A higher cutoff (*p*<5×10^−6^) was applied to screen the SNPs and eliminate interference from high linkage disequilibrium (*r* ^2^ = 0.001 within a clumping window of 10,000 kb) according to the 1000 genomes European project (Abecasis et al. 2012; Ference et al. 2015). In addition, the strength of individual SNPs was quantified using *F*‐statistics, and an *F*‐statistics value > 10 was considered sufficiently reliable to prevent bias from poor instruments (Park et al. 2010). The PhenoScanner database (http://www.phenoscanner.medschl.cam.ac.uk/) was used to identify additional features related to the selected SNPs that may impact the mentioned neurological disorders. Finally, 11 SNPs were identified as IVs for plasma HIF‐1α levels in the study (Table S4).

### 2.4 Two‐Sample MR

Based on the above GWAS statistics, the two‐sample MR analysis was conducted between plasma HIF‐1α levels and various neurological diseases to determine whether plasma HIF‐1α levels have a causal relationship with the onset of neurological diseases. The inverse‐variance‐weighted MR (IVW) method was the primary analysis to detect the association, with MR‐Egger regression, weighted median, simple mode, and weighted mode methods conducted to support the IVW estimates.

### 2.5 Statistical Analysis

The MR‐Egger intercept test and the Mendelian Randomization Pleiotropy RESidual Sum and Outlier (MR‐PRESSO) methods were applied to mitigate the bias caused by horizontal pleiotropy, which could invalidate the results (*p* < 0.05). The Cochran's Q‐test analysis was performed to test for heterogeneity among the included SNPs, with *p* > 0.05 indicating no heterogeneity. The leave‐one‐SNP‐out analysis was performed to estimate the contribution of each SNP to the results.

All statistical analyses were completed using the Two‐Sample MR (Hemani et al. 2018) and MR‐PRESSO packages (Verbanck et al. 2018) in *R* software (version 4.2.2). The online power calculator was used to calculate the power (Brion et al. 2013) (https://shiny.cnsgenomics.com/mRnd/).

## 3 Results

### 3.1 Causal Effect of Plasma HIF‐1α on Cerebrovascular Diseases

**FIGURE 2:**
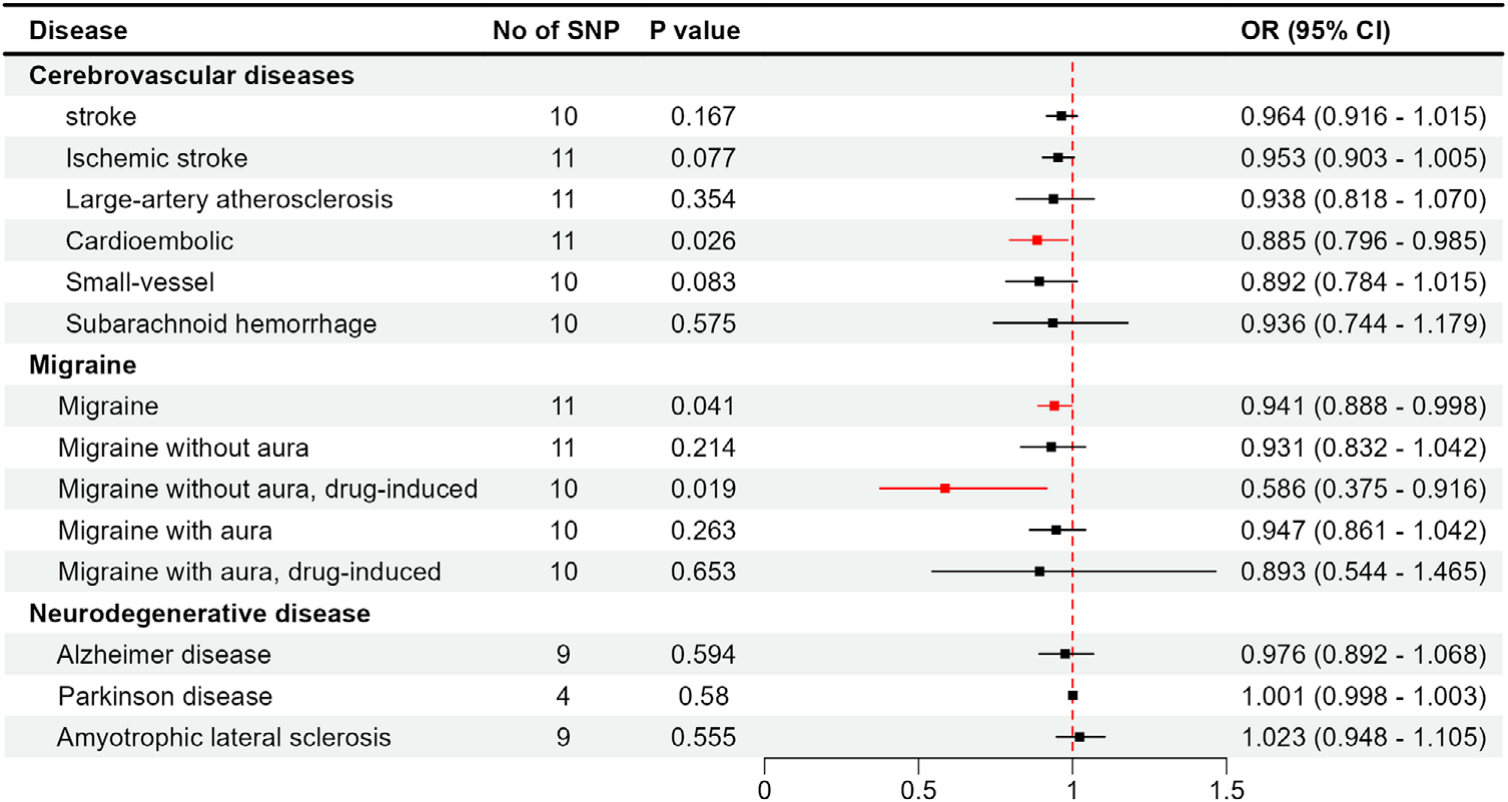
Mendelian randomization analysis estimates of plasma HIF‐1α and the risk of neurological diseases. CI, confidence interval; OR, odds ratio.

For the cerebrovascular diseases, a higher plasma level of HIF‐1α was associated with a decreased incidence of CES (odds ratio [OR] = 0.885; 95% confidence interval [CI] = 0.796−0.985, *p* = 0.026) in the IVW method (Figure 2), with a statistical power of 88% (Table S2). The results were reinforced by the weighted median method (OR = 0.847; 95% CI = 0.731−0.981, *p* = 0.027) (Table 1). However, there was no causal effect of plasma HIF‐1α on AS (OR = 0.964; 95% CI = 0.916−1.015, *p* = 0.167), AIS (OR = 0.953; 95% CI = 0.903−1.005, *p* = 0.077), LAS (OR = 0.938; 95% CI = 0.818−1.070, *p* = 0.354), SVS (OR = 0.892; 95% CI = 0.784−1.015, *p* = 0.083), and SAH (OR = 0.936; 95% CI = 0.744−1.179, *p* = 0.575) (Figure 2). There was no substantial horizontal pleiotropy for the different outcomes, as verified by the MR‐Egger intercept and MR‐PRESSO global test, and no heterogeneity was indicated by Cochran's Q test (Table 2).

**TABLE 1:** Sensitivity analysis of the associations between plasma HIF‐1α levels and neurological diseases.

Outcome	MR‐Egger OR (95% CI) *p* value	Weighted median OR (95% CI) *p* value	Simple mode OR (95% CI) *p* value	Weighted mode OR (95% CI) *p* value
Cerebrovascular diseases				
Any stroke	0.979 (0.883–1.086) 0.700	0.959 (0.896–1.026) 0.223	0.950 (0.849–1.063) 0.398	0.952 (0.855–1.060) 0.394
Any ischemic stroke	1.007 (0.903–1.124) 0.899	0.958 (0.891–1.031) 0.255	0.963 (0.855–1.085) 0.547	0.965 (0.865–1.076 0.533
Large‐artery atherosclerosis	0.951 (0.719–1.259) 0.736	0.893 (0.744–1.071) 0.224	0.851 (0.634–1.144) 0.312	0.852 (0.634–1.145) 0.312
Cardioembolic	0.888 (0.710–1.111) 0.327	0.847 (0.731–0.981) **0.027**	0.784 (0.607–1.013) 0.093	0.781 (0.600–1.017) 0.096
Small‐vessel	0.833 (0.641–1.099) 0.238	0.909 (0.763–1.082) 0.283	0.894 (0.684–1.170) 0.436	0.892 (0.669–1.191) 0.459
Subarachnoid hemorrhage	1.039 (0.623–1.735) 0.887	0.872 (0.642–1.186) 0.384	0.874 (0.535–1.426) 0.602	0.823 (0.537–1.260) 0.393
Migraine				
Migraine	0.883 (0.785–0.994) 0.069	0.947 (0.876–1.023) 0.166	0.979 (0.870–1.101) 0.727	0.967 (0.864–1.080) 0.561
Migraine without aura	0.851 (0.677–1.070) 0.201	0.960 (0.842–1.095) 0.542	0.981 (0.818–1.176) 0.836	0.968 (0.816–1.148) 0.714
Migraine without aura, drug‐induced	0.509 (0.215–1.206) 0.163	0.538 (0.305–0.949) **0.032**	0.464 (0.200–1.077) 0.107	0.493 (0.236–1.029) 0.009
Migraine with aura	0.937 (0.758–1.157) 0.560	1.009 (0.887–1.147) 0.894	1.030 (0.832–1.275) 0.792	1.019 (0.852–1.218) 0.843
Migraine with aura, drug‐induced	0.730 (0.231–2.301) 0.606	0.660 (0.351–1.242) 0.198	0.638 (0.258–1.578) 0.356	0.626 (0.284–1.378) 0.274
Neurodegenerative disease				
Parkinson's disease	1.001 (0.989–1.012) 0.905	1.001 (0.998–1.004) 0.590	1.001 (0.997–1.005) 0.554	1.001 (0.998–1.005) 0.554
Alzheimer's disease	0.939 (0.774–1.137) 0.538	0.980 (0.872–1.102) 0.741	0.889 (0.723–1.094) 0.300	1.063 (0.857–1.317) 0.594
Amyotrophic lateral sclerosis	0.989 (0.846–1.157) 0.898	1.032 (0.938–1.135) 0.519	1.033 (0.905–1.179) 0.642	1.036 (0.897–1.196) 0.644

**TABLE 2:** Heterogeneity and pleiotropy tests for the associations of plasma HIF‐1α levels with neurological diseases.

Outcome	Cochrane's *Q* test	MR‐Egger intercept test			MRPRESSO global test *Q*‐value
	*Q*‐pval	Intercept		*p* intercept	
Cerebrovascular diseases					
Any stroke	0.745	−0.004	0.745		0.741
Any ischemic stroke	0.657	−0.012	0.283		0.666
Large‐artery atherosclerosis	0.867	−0.003	0.9083		0.88
Small‐vessel	0.548	0.013	0.627		0.588
Cardioembolic	0.513	−0.001	0.975		0.523
Subarachnoid hemorrhage	0.503	−0.020	0.667		0.553
Migraine					
Migraine	0.900	0.013	0.256		0.907
Migraine without aura	0.166	0.019	0.398		0.221
Migraine without aura,, drug‐induced	0.835	0.033	0.718		0.861
Migraine with aura	0.631	0.002	0.910		0.621
Migraine with aura, drug‐induced	0.362	0.039	0.710		0.399
Neurodegenerative disease					
Parkinson's disease	0.680	−1.734615e−05	0.985		0.707
Alzheimer's disease	0.303	0.009	0.662		0.303
Amyotrophic lateral sclerosis	0.809	0.008	0.643		0.826
				

### 3.2 Causal Effect of Plasma HIF‐1α on Migraine

Under the IVW model, higher plasma HIF‐1α levels were negatively associated with the risk of migraine (OR = 0.941, 95% CI = 0.888–0.998, *p* = 0.041) and MOAD (OR = 0.586, 95% CI = 0.375–0.916, *p* = 0.019) (Figure 2). Similar results for the association with MOAD were obtained from weighted median analysis (OR = 0.538, 95% CI = 0.305−0.949, *p* = 0.032) (Table 1). Extending the MR analysis to migraine subtypes, there was no evidence to suggest that plasma HIF‐1 levels were associated with the risk of MOA (OR = 0.931, 95% CI = 0.832−1.042, *p =* 0.214), MA (OR = 0.947, 95% CI = 0.861−1.042, *p =* 0.263), and MAD (OR = 0.893, 95% CI = 0.544−1.465, *p =* 0.653) (Figure 2). There was no indication of heterogeneity in the sensitivity analysis (*p* > 0.05) or horizontal pleiotropy (*p* > 0.05) among these SNPs. The MR‐PRESSO global test likewise was unable to detect any discernible pleiotropy (*p* > 0.05) (Table 1). The genetic association between levels of plasma HIF‐1α and neurodegenerative diseases (PD, AD, ALS, and MS) was not detected (*p* > 0.05) (Figure 2). Additional files include scatter plots, funnel plots, leave‐one‐out analyses, and plot forest layouts (Figures S1–S14).

**FIGURE 3:**
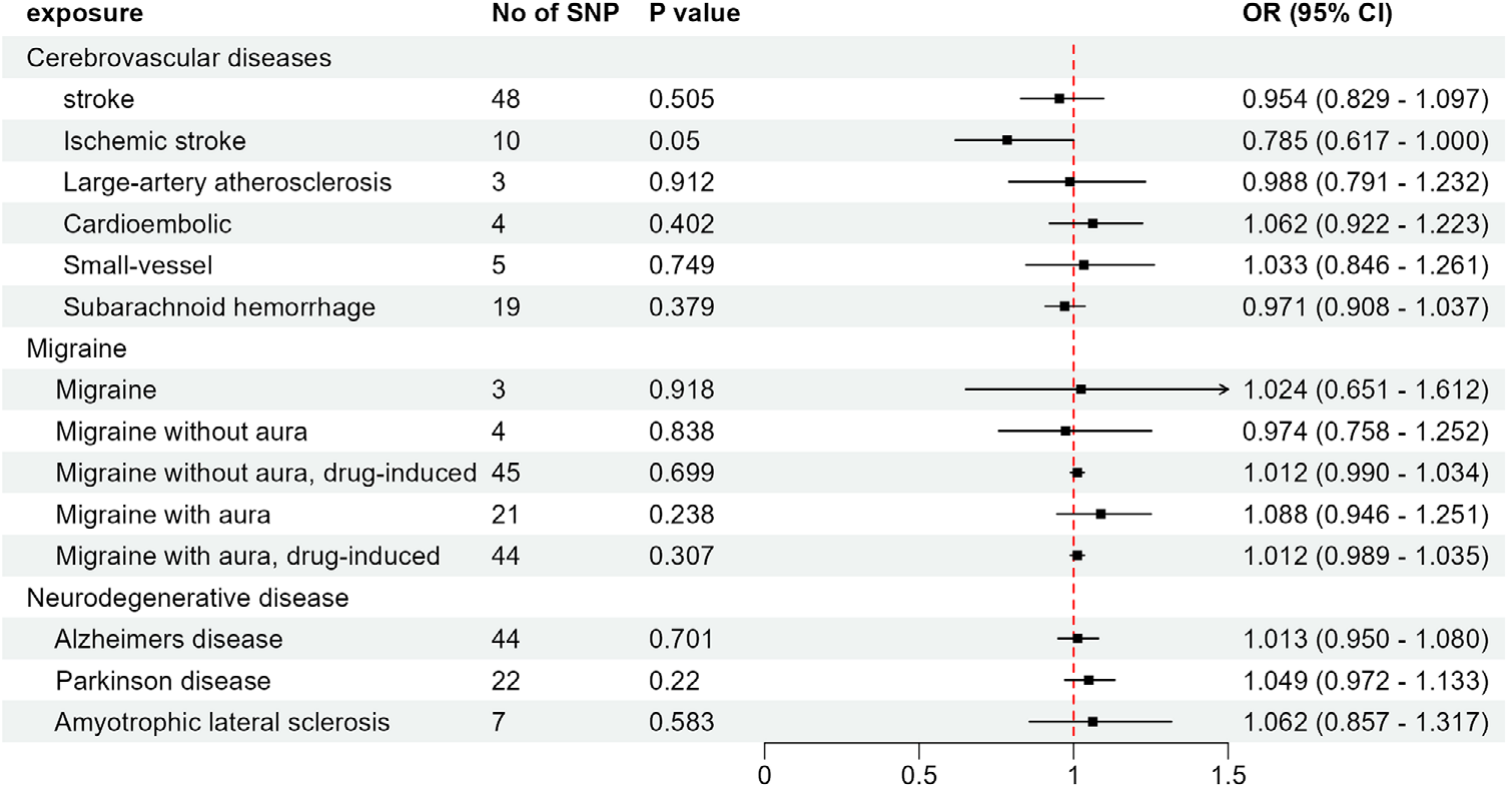
Reverse mendelian randomization analysis estimates of the risk of neurological diseases and the level of plasma HIF‐1α. CI, confidence interval; OR, odds ratio; SNP, single‐nucleotide polymorphism.

The reverse‐MR analysis revealed that the aforementioned neurological diseases did not have a causal effect on plasma HIF‐1α levels (Figure 3) (Table S3). There was no weak bias of IVs because all SNPs of exposure considered in the analysis had an *F*‐statistic larger than 10 (Table S4).

## 4 Discussion

Using a two‐sample MR method, we provide a thorough investigation to determine the causal relationships between plasma HIF‐1α levels and various neurological disorders. Our research indicates a causal inference between plasma HIF‐1α levels and the risk of CES, migraine, and drug‐induced MOA. We found that genetically predicted higher plasma HIF‐1α levels were associated with CES, migraine, and drug‐induced MOA. Meanwhile, there is no causal association between the above neurological diseases and plasma HIF‐1α by the reverse MR analyses.

HIF‐1α has been proven to be an essential regulator of oxygen homeostasis, which is overexpressed in hypoxic tissues and can help the body adapt to low oxygen levels (Haddad 2003). Hypoxia can activate inflammation, and ischemia‐related inflammation affects the prognosis of various organs exposed to ischemia (Eltzschig and Carmeliet 2011). In experimental cerebral ischemia, zinc was found to significantly increase HIF‐1α expression in astrocytes and promote angiogenesis during cerebral ischemic repair through the VEGF pathway (Li et al. 2022). Additionally, HIF‐1α also plays crucial roles in metabolism, glucose transport, angiogenesis, and erythropoiesis (He et al. 2021; Giusti and Fiszer de Plazas 2012). There is growing evidence that HIF‐1α is present in bodily fluids such as plasma and serum, although its origin remains unclear (Maciejewska et al. 2023; Heikal et al. 2018). Evidence shows that plasma HIF‐1α levels in remote ischemic preconditioning mice and patients were significantly higher than that in controls, suggesting that HIF‐1α can enhance hypoxia tolerance in mice (Li et al. 2019; Zeng et al. 2023). The size of the cerebral infarction after an acute IS was found to be associated with serum HIF‐1α concentrations (Xue et al. 2017; Amalia et al. 2020) and closely related to trauma severity in patients with traumatic brain injury (Lv et al. 2021). However, due to the potential for bias caused by possible unmeasured or unknown confounders, only a correlation between HIF‐1α levels and the relevant disorders can be shown by these observational studies.

We discovered that genetically determined plasma HIF‐1α may negatively impact CES but not SVS or LAS in the examination of IS subtypes. It is well‐known that IS subtypes may differ in the genetic pathophysiological mechanisms (Traylor et al. 2012). Compared to other stroke subtypes, CES has a more inflammatory environment, macrophage content, and greater platelet content in thrombus (Maida et al. 2020). Furthermore, several studies have shown that HIF‐1α activates downstream genes to control hypoxia responses, promote blood vessel growth, improve myocardial infarction, and increase the survival rate of patients with acute myocardial ischemia (Zheng et al. 2018; Jianqiang et al. 2015). Therefore, we speculated that increased HIF‐1α levels would protect against myocardial damage and mediate inflammation, thereby reducing the risk of CES stroke. It is necessary to perform additional investigation to decipher the exact mechanism behind the causal relationship between HIF‐1α and CES.

Migraine is a common neurological disease characterized by recurrent headaches, nausea, and/or vomiting related to light and sound sensitivity (Cooper et al. 2020). The risk factors for migraine progression and occurrence include genetic, environmental, age, and hormonal differences (Hranilovich et al. 2021). It is widely accepted that both neuroinflammation and oxidative stress are crucial factors in the development of migraine (Goschorska et al. 2020). A one SD increase in plasma HIF‐1α correlated with a 5.9% and 41.4% reduction in the probability of the condition using migraine and drug‐induced MOA as the outcome, respectively. However, MA and MOA did not reach the significance level, indicating a suggestive causal relationship of plasma HIF‐1α with migraine and drug‐induced MOA, but not with other subtypes. In nitroglycerin‐injected mice, preventing HIF‐1α degradation ameliorated migraine‐like presentation and inhibited central pain sensitization (Yang et al. 2023). Yet, a cross‐sectional study in female pediatric migraine patients found that serum HIF‐1α is significantly higher than the healthy control group (Kilinc et al. 2023). It is not known whether age and sex influenced the results. Additional studies are required to address the multifactorial pathogenesis and therapeutic targets of migraine.

Surprisingly, we did not discover the causal connection between plasma HIF‐1α and SAH, MS, AD, PD, and ALS. Several studies have shown that HIF‐1α restrained the pathogenesis of AD by reducing tau protein phosphorylation, neuron inflammation, and death (Ashok et al. 2017). The production of ROS and autophagy disorders is present in the brain at various stages of multiple sclerosis, which are mediated by HIF‐1α (Asgari et al. 2022). We do not have proof of associations between plasma HIF‐1α and the above diseases due to the analysis's restricted capability.

### 4.1 Strengths and Limitations

This MR study provided population‐based proof for the genetic associations between HIF‐1α levels and neurological diseases. Our research's main advantages are its large‐scale GWAS cases and thorough analysis, which exclude the possibility of bias from some unidentified confounders and allow us to use extensive genetic data on a variety of neurological diseases. Additionally, horizontal pleiotropy and heterogeneity need to be examined to confirm the present assumptions. Consistent results were achieved in the sensitivity analyses, MR‐PRESSO global test, and MR‐Egger intercept test, and the findings were not impacted by pleiotropy. The heterogeneity effect was not demonstrated by using Cochrane's Q test.

There are a few limitations. First, the genetic instruments were developed using patient data of European heritage. The interaction between genotype and environment might impact the results. Therefore, the generalizability to other racial or ethnic populations is limited. Second, given the existence of the blood‐brain barrier, the plasma HIF‐1α level might not precisely reflect the changes in the brain. Further study of HIF‐1α in cerebrospinal fluid is necessary. Lastly, our study lacks multiple testing corrections, which may increase the risk of Type I errors. However, given the exploratory nature of our analysis and the modest effect sizes typically observed in complex diseases, we chose to present uncorrected *p* values to avoid missing potentially meaningful signals. This approach is consistent with other MR studies in the field. Future studies with larger sample sizes and independent cohorts are needed to validate our findings and further explore the causal relationships identified in this study.

## 5 Conclusion

According to our research, elevated plasma levels of HIF‐1α are causally correlated to a decreased incidence of CES, migraine, and drug‐induced MOA. Our research offers a novel understanding of the role of HIF‐1α in neurological illnesses, regardless of the precise mechanism linking it to genetic background.

## Author Contributions

**Jing Liang**: writing–review and editing, writing–original draft, methodology, software, investigation, formal analysis, conceptualization, visualization. **Xiaoyan Du**: writing–review and editing, methodology, writing–original draft. **Mengfei Wang**: writing–original draft, methodology, investigation. **Hongqin Zheng**: writing–review and editing, software. **Yang Sun**: software, methodology. **Yi Lin**: conceptualization, methodology, supervision, project administration, validation, data curation, writing–review and editing, resources.

## Ethics Statement

The ethics committee of the original GWASs authorized the protocol and data. No separate ethical approval was required.

## Conflicts of Interest

The authors declare no conflicts of interest.

### Peer Review

The peer review history for this article is available at https://publons.com/publon/10.1002/brb3.70398.

## Supporting information



Supporting Information

Supporting Information

## Data Availability

The data that support the findings of this study are available from the corresponding author upon reasonable request.
