# Supplementary material for: Association Between Hypoxia‐Inducible Factor‐1α and Neurological Diseases: A Bidirectional Two‐Sample Mendelian Randomization Analysis
Source: Brain Behav. 2025 Feb 28;15(3):e70398. doi: 10.1002/brb3.70398 (PMC11870835; doi:10.1002/brb3.70398)
Supplement: Supplementary file 1 — Supporting Information [file BRB3-15-e70398-s002.docx]

**Supplementary files**

**Supplementary Tables**

Table S1. Detailed information of the GWAS data. …………………………………………………………………………………………………...............…………………3

Table S2.Power calculation for two-sample MR analyses of HIF-1α on neurological diseases ……………………………………………………...... .........…………………5

Table S3. Reverse causal relationships of plasma HIF-1α with neurological diseases performed by MR.…………………………………………………….…………………7

Table S4**.** Characteristics of selected SNPs for plasma HIF-1α. ……………………………………………………………………………………………….…………………13

Table S5. Characteristics of selected SNPs for neurological diseases.…………………………………………………………………………………….………………………14

**Supplementary Figures**

Figure S1. The causal effect of plasma HIF-1α on any stroke risk………………………………………………………………………………………….…………………42

Figure S2. The causal effect of plasma HIF-1α on ischemic stroke risk………………………………………………………………………………..………………………43

Figure S3. The causal effect of plasma HIF-1α on ischemic stroke (large artery atherosclerosis) risk………………………………………………………….………………44

Figure S4. The causal effect of plasma HIF-1α on ischemic stroke (cardioembolic) risk………………………………………………………………………………………45

Figure S5. The causal effect of plasma HIF-1α on ischemic stroke (Small-vessel) risk………………………………………………………… ……………………………46

Figure S6. The causal effect of plasma HIF-1α on subarachnoid haemmorrhage risk…………………………………………………………………………………………47

Figure S7. The causal effect of plasma HIF-1α on migraine risk……………………………………………………………………………………………………………….48

Figure S8. The causal effect of plasma HIF-1α on migraine (without aura) risk……………………………………………………………………….………………………49

Figure S9. The causal effect of plasma HIF-1α on migraine (without aura, drug-induced) risk………………………………………………………….……………………50

Figure S10. The causal effect of plasma HIF-1α on migraine (with aura) risk………………………………………………………….………………………………………51

Figure S11. The causal effect of plasma HIF-1α on migraine (with aura, drug-induced) risk…………………………………………………………….…………………….52

Figure S12. The causal effect of plasma HIF-1α on Parkinson’s disease risk…………………………………………………………………………..………………………53

Figure S13. The causal effect of plasma HIF-1α on Alzheimer’s disease risk……………………………………………………………………………………..……………54

Figure S14 The causal effect of plasma HIF-1α on Amyotrophic lateral sclerosis risk…………………………………………………………………………..……………55

**Table S1** Detailed information of the GWAS data.

| **Phenotype** | **Sample size**  **(cases/controls)** | **Population** | **Consortium** | **Year** | **Journal** |
| --- | --- | --- | --- | --- | --- |
| **plasma HIF-1α** | 3301 | European | **-** | 2018 | Nature |
| **Cerebrovascular diseases** |  |  |  |  |  |
| any stroke | 40,585/406,111 | European | MEGASTROKE | 2018 | Nat Genet |
| any ischemic stroke | 34,217/406,111 | European | MEGASTROKE | 2018 | Nat Genet |
| large artery atherosclerosis | 4373/406,111 | European | MEGASTROKE | 2018 | Nat Genet |
| cardioembolic | 7193/406,111 | European | MEGASTROKE | 2018 | Nat Genet |
| small-vessel | 5386/192,662 | European | MEGASTROKE | 2018 | Nat Genet |
| subarachnoid hemorrhage | 1338/201,230 | European | FinnGen | 2021 | **-** |
| **Migraine** |  |  |  |  |  |
| migraine | 8,547/176,107 | European | FinnGen | 2021 | **-** |
| migraine without aura | 3,215/176,107 | European | FinnGen | 2021 | **-** |
| migraine without aura, drug induced | 321/376,956 | European | FinnGen | 2021 | **-** |
| migraine with aura | 3,541/176,107 | European | FinnGen | 2021 | **-** |
| migraine with aura, drug-induced | 307/376,970 | European | FinnGen | 2021 | **-** |
| **Neurodegenerative disease** |  |  |  |  |  |
| parkinson’s Disease | 33,674/449,056 | European | IPDGC | 2019 | Lancet Neurol |
| alzheimer’s Disease | 21,982/41,944 | European | IGAP | 2019 | Nat Genet |
| amyotrophic lateral sclerosis | 20,806/59,804 | European | AVS | 2018 | Neuron |

**Table S2.** Power calculation for two-sample MR analysis of HIF-1α on neurological diseases.

| **Outcome** | **Sample size** | **Proportion of cases** | **OR** | **R^2^** | **Power** |
| --- | --- | --- | --- | --- | --- |
| **Cerebrovascular diseases** |  |  |  |  |  |
| stroke | 446,696 | 0.09 | 0.964 | 0.071 | 45% |
| Ischemic stroke | 440,328 | 0.08 | 0.988 | 0.078 | 9% |
| -large artery atherosclerosis | 410,484 | 0.01 | 0.966 | 0.085 | 10% |
| -cardioembolic | 413,304 | 0.02 | 0.880 | 0.085 | 88% |
| -small-vessel | 198,048 | 0.03 | 0.879 | 0.085 | 77% |
| Subarachnoid hemorrhage | 202,568 | 0.01 | 0.936 | 0.085 | 13% |
| **Neurodegenerative disease** |  |  |  |  |  |
| Alzheimer’s Disease | 63,926 | 0.34 | 0.976 | 0.062 | 11% |
| Parkinson’s Disease | 482,730 | 0.07 | 0.974 | 0.062 | 21% |
| Amyotrophic lateral sclerosis | 80,610 | 0.26 | 1.023 | 0.085 | 13% |
| **Migraine** |  |  |  |  |  |
| Migraine | 306,314 | 0.060 | 0.950 | 0.085 | 48% |
| Migraine without aura | 295,754 | 0.026 | 0.942 | 0.085 | 31% |
| Migraine without aura，drug-induced | 377,277 | 0.0009 | 0.586 | 0.071 | 53% |
| Migraine with aura | 294,567 | 0.023 | 0.965 | 0.078 | 13% |
| Migraine with aura, drug-induced | 377,277 | 0.0008 | 0.893 | 0.070 | 8% |

**Table S3.** Reverse causal relationships of plasma HIF-1α with neurological diseases performed by MR.

| **Exposure** | **nSNPs** | **Method** | **OR (95%CI)** | **P value** | **Q pval** | **intercept**  ***p* value** | **Global *P*** |
| --- | --- | --- | --- | --- | --- | --- | --- |
| **stroke** | **48** | IVW | **0.954(0.829-1.097)** | **0.505** | **0.578** |  | **0.586** |
|  |  | MR Egger | **0.838(0.538-1.305)** | **0.437** |  | **0.549** |  |
|  |  | Weighted Median | **0.900(0.733-1.106)** | **0.317** |  |  |  |
|  |  | Simple mode | **0.689(0.409-1.158)** | **0.166** |  |  |  |
|  |  | Weighted mode | **0.664(0.389-1.132)** | **0.139** |  |  |  |
| Ischemic stroke | 10 | IVW | 0.785(0.617-1.000) | 0.050 | 0.770 |  | 0.761 |
|  |  | MR Egger | 1.674(0.271-10.344) | 0.594 |  | 0.4355 |  |
|  |  | Weighted Median | 0.772(0.562-1.059) | 0.108 |  |  |  |
|  |  | Simple mode | 0.634(0.366-1.097) | 0.138 |  |  |  |
|  |  | Weighted mode | 0.621(0.359-1.073) | 0.12 |  |  |  |
| Ischemic stroke -LAS | 3 | IVW | 0.988(0.791-1.232) | 0.912 | 0.252 |  |  |
|  |  | MR Egger | 0.544(0.043-6.816) | 0.719 |  | 0.723 |  |
|  |  | Weighted Median | 0.988(0.772-1.264) | 0.921 |  |  |  |
|  |  | Simple mode | 0.967(0.701-1.333) | 0.855 |  |  |  |
|  |  | Weighted mode | 0.985(0.730-1.329) | 0.929 |  |  |  |
| Ischemic stroke-SVS | 5 | IVW | 1.033(0.846-1.261) | 0.749 | 0.261 |  | 0.331 |
|  |  | MR Egger | 0.824(0.423-1.604) | 0.608 |  | 0.532 |  |
|  |  | Weighted Median | 1.015(0.805-1.280) | 0.900 |  |  |  |
|  |  | Simple mode | 0.979(0.674-1.423) | 0.917 |  |  |  |
|  |  | Weighted mode | 0.932(0.658-1.320） | 0.712 |  |  |  |
| Ischemic stroke-CES | 4 | IVW | 1.062(0.922-1.223) | 0.402 | 0.999 |  | 0.999 |
|  |  | MR Egger | 1.053(0.794-1.394) | 0.756 |  | 0.947 |  |
|  |  | Weighted Median | 1.059(0.908-1.234) | 0.467 |  |  |  |
|  |  | Simple mode | 1.060(0.863-1.302) | 0.615 |  |  |  |
|  |  | Weighted mode | 1.057(0.884-1.264) | 0.585 |  |  |  |
| Subarachnoid hemorrhage | 19 | IVW | 0.971(0.908-1.037) | 0.379 | 0.390 |  | 0.493 |
|  |  | MR Egger | 0.968(0.867-1.080) | 0.573 |  | 0.943 |  |
|  |  | WM | 0.981(0.898-1.071) | 0.664 |  |  |  |
|  |  | Simple mode | 0.977(0.856-1.116) | 0.743 |  |  |  |
|  |  | Weighted mode | 0.976(0.888-1.072) | 0.623 |  |  |  |
| Migraine | 3 | IVW | 1.024(0.651-1.612) | 0.918 | 0299 |  |  |
|  |  | MR Egger | 0.857(4.185e-09-1.769e+08) | 0.990 |  | 0.988 |  |
|  |  | WM | 0.881(0.537-1.447) | 0.618 |  |  |  |
|  |  | Simple mode | 0.853(0.442-1.649) | 0.684 |  |  |  |
|  |  | Weighted mode | 0.834(0.446-1.560) | 0.628 |  |  |  |
| Migraine with aura | 21 | IVW | 1.088(0.946-1.251) | 0.238 | 0.853 |  | 0.866 |
|  |  | MR Egger | 1.183(0.724-1.932) | 0.510 |  | 0.731 |  |
|  |  | WM | 0.999(0.832-1.199) | 0.988 |  |  |  |
|  |  | Simple mode | 0.973(0.681-1.393) | 0.885 |  |  |  |
|  |  | Weighted mode | 0.962(0.722-1.284) | 0.797 |  |  |  |
| Migraine without aura | 4 | IVW | 0.974(0.758-1.252) | 0.838 | 0.826 |  | 0.837 |
|  |  | MR Egger | 0.895(0.008-100.219) | 0.967 |  | 0.975 |  |
|  |  | WM | 0.965(0.730-1.275) | 0.801 |  |  |  |
|  |  | Simple mode | 0.944 (0.634-1.404) | 0.793 |  |  |  |
|  |  | Weighted mode | 0.950 (0.636-1.420) | 0.818 |  |  |  |
| Migraine without aura**，**drug-induced | 45 | IVW | 1.012(0.990-1.034) | 0.300 | 0.699 |  | 0.685 |
|  |  | MR Egger | 1.019(0.979-1.061) | 0.362 |  | 0.668 |  |
|  |  | WM | 1.015(0.980-1.050) | 0.402 |  |  |  |
|  |  | Simple mode | 1.010(0.942-1.083) | 0.787 |  |  |  |
|  |  | Weighted mode | 1.023(0.968-1.082) | 0.426 |  |  |  |
| Migraine with aura, drug-induced | 44 | IVW | 1.012(0.989-1.035) | 0.307 |  |  |  |
|  |  | MR Egger | 0.999(0.942-1.055) | 0.905 | 0.941 |  | 0.942 |
|  |  | WM | 1.021(0.989-1.055) | 0.194 |  | 0.566 |  |
|  |  | Simple mode | 1.056(0.979-1.139) | 0.166 |  |  |  |
|  |  | Weighted mode | 1.056(0.983-1.135) | 0.144 |  |  |  |
| Alzheimer’ s Disease | 44 | IVW | 1.013(0.950-1.080) | 0.701 | 0.222 |  | 0.552 |
|  |  | MR Egger | 1.075(0.985-1.173) | 0.112 |  | 0.063 |  |
|  |  | WM | 1.032(0.943-1.130) | 0.488 |  |  |  |
|  |  | Simple mode | 1.011(0.84401.211) | 0.906 |  |  |  |
|  |  | Weighted mode | 1.054(0.968-1.148) | 0.231 |  |  |  |
| Parkinson’s Disease | 22 | IVW | 1.049(0.972-1.133) | 0.220 | 0.910 |  | 0.894 |
|  |  | MR Egger | 0.952(0.793-1.144) | 0.608 |  | 0.269 |  |
|  |  | WM | 1.063(0.953-1.186) | 0.275 |  |  |  |
|  |  | Simple mode | 1.069(0.880-1.298) | 0.510 |  |  |  |
|  |  | Weighted mode | 1.069(0.901-1.267) | 0.453 |  |  |  |
| Amyotrophic lateral sclerosis | 7 | IVW | 1.062(0.857-1.317) | 0.583 | 0.397 |  | 0.416 |
|  |  | MR Egger | 0.801(0.510-1.258) | 0.407 |  | 0.259 |  |
|  |  | WM | 1.033(0.799-1.337) | 0.803 |  |  |  |
|  |  | Simple mode | 1.068(0.715-1.595) | 0.765 |  |  |  |
|  |  | Weighted mode | 0.961(0.722-1.280) | 0.800 |  |  |  |

**Table S4.** Characteristics of selected SNPs for plasma HIF-1α.

| **SNP** | **Trait** | **chr** | **pos** | **Effect**  **allele** | **Other**  **allele** | **eaf** | **beta** | **SE** | ***P* value** | **R2** | **F** |
| --- | --- | --- | --- | --- | --- | --- | --- | --- | --- | --- | --- |
| rs78974779 | HIF-1α | 1 | 1.59E+08 | C | T | 0.08182 | -0.222 | 0.0477 | 3.24E-06 | 0.006519 | 21.64741 |
| rs16831952 | HIF-1α | 1 | 44864216 | G | A | 0.01613 | -0.4775 | 0.1006 | 2.04E-06 | 0.006779 | 22.51581 |
| rs752937 | HIF-1α | 2 | 67311244 | C | T | 0.18114 | 0.1524 | 0.0325 | 2.69E-06 | 0.006617 | 21.97556 |
| rs76050618 | HIF-1α | 2 | 25540280 | T | G | 0.01266 | -0.5099 | 0.111 | 4.37E-06 | 0.006352 | 21.08924 |
| rs9791261 | HIF-1α | 6 | 62284928 | C | T | 0.04212 | 0.3059 | 0.0619 | 7.76E-07 | 0.007344 | 24.407 |
| rs75596147 | HIF-1α | 7 | 48281782 | T | A | 0.02714 | -0.4445 | 0.0822 | 6.46E-08 | 0.008781 | 29.2238 |
| rs10750581 | HIF-1α | 11 | 1.35E+08 | G | A | 0.66241 | 0.1242 | 0.027 | 4.07E-06 | 0.006369 | 21.14718 |
| rs117244611 | HIF-1α | 12 | 1.26E+08 | A | G | 0.01625 | 0.526 | 0.0973 | 6.46E-08 | 0.008776 | 29.20671 |
| rs906310 | HIF-1α | 15 | 98304530 | A | G | 0.42918 | -0.1136 | 0.0247 | 4.27E-06 | 0.006367 | 21.13974 |
| rs11072221 | HIF-1α | 15 | 71132566 | C | G | 0.70913 | -0.1274 | 0.0273 | 3.02E-06 | 0.006554 | 21.76458 |
| rs118190027 | HIF-1α | 16 | 52083815 | T | C | 0.02857 | -0.3757 | 0.0799 | 2.57E-06 | 0.006653 | 22.09661 |

SNP, single-nucleotide polymorphism**;** Chr, chromosome; Pos, position; EAF, Effect allele frequency; SE, standard error; The threshold was set at *P* < 1×10^-6^.

**Table S5.** Characteristics of selected SNPs for neurodegenerative diseases.

| **SNP** | **Trait** | **Chr** | **Pos.** | **Effect allele** | **Other allele** | **EAF** | **Beta** | **SE** | ***P value*** | **R^2^** | **F statistic** |
| --- | --- | --- | --- | --- | --- | --- | --- | --- | --- | --- | --- |
| rs11957829 | stroke | 5 | 121515195 | A | G | 0.8315 | 0.0616 | 0.0128 | 1.51E-06 | 5.18E-05 | 23.16005 |
| rs4942561 | stroke | 13 | 47209347 | T | G | 0.7581 | 0.064 | 0.0107 | 2.05E-09 | 8.01E-05 | 35.77589 |
| rs2107595 | stroke | 7 | 19049388 | A | G | 0.1671 | 0.0803 | 0.0121 | 3.59E-11 | 9.86E-05 | 44.04113 |
| rs79280766 | stroke | 12 | 122633031 | A | G | 0.0362 | 0.1434 | 0.0303 | 2.20E-06 | 5.01E-05 | 22.3981 |
| rs72889922 | stroke | 2 | 173321704 | A | G | 0.015 | 0.2504 | 0.0535 | 2.91E-06 | 4.90E-05 | 21.9058 |
| rs36053597 | stroke | 11 | 124606484 | T | C | 0.1688 | 0.061 | 0.0121 | 4.55E-07 | 5.69E-05 | 25.41482 |
| rs7294375 | stroke | 12 | 90247390 | T | G | 0.7289 | -0.053 | 0.0104 | 3.33E-07 | 5.81E-05 | 25.97067 |
| rs1549758 | stroke | 7 | 150695726 | T | C | 0.3125 | 0.0532 | 0.0104 | 3.11E-07 | 5.86E-05 | 26.16704 |
| rs42039 | stroke | 7 | 92244422 | T | C | 0.2455 | -0.0574 | 0.011 | 1.65E-07 | 6.10E-05 | 27.2293 |
| rs650724 | stroke | 13 | 110804809 | A | G | 0.0938 | -0.0793 | 0.0167 | 2.17E-06 | 5.05E-05 | 22.54818 |
| rs7219031 | stroke | 17 | 56445908 | A | G | 0.1933 | 0.0596 | 0.0116 | 2.59E-07 | 5.91E-05 | 26.39822 |
| rs6872625 | stroke | 5 | 3092254 | T | G | 0.2381 | 0.051 | 0.0111 | 4.21E-06 | 4.73E-05 | 21.1102 |
| rs7124178 | stroke | 11 | 9283278 | T | C | 0.7523 | -0.0524 | 0.0107 | 8.98E-07 | 5.37E-05 | 23.98242 |
| rs28441665 | stroke | 4 | 55083056 | T | C | 0.928 | -0.0852 | 0.0181 | 2.35E-06 | 4.96E-05 | 22.15747 |
| rs75630045 | stroke | 7 | 115508909 | A | G | 0.0144 | 0.2002 | 0.043 | 3.29E-06 | 4.85E-05 | 21.67651 |
| rs1537375 | stroke | 9 | 22116071 | T | C | 0.4979 | -0.0519 | 0.0091 | 1.24E-08 | 7.28E-05 | 32.52745 |
| rs1537407 | stroke | 1 | 3345705 | T | C | 0.7984 | -0.0662 | 0.0131 | 4.48E-07 | 5.72E-05 | 25.53709 |
| rs79332111 | stroke | 5 | 85398495 | T | C | 0.8805 | -0.0661 | 0.0145 | 4.85E-06 | 4.65E-05 | 20.78093 |
| rs34311906 | stroke | 4 | 113732090 | T | C | 0.5989 | -0.0548 | 0.0108 | 3.64E-07 | 5.76E-05 | 25.74611 |
| rs72699046 | stroke | 1 | 113038761 | C | G | 0.0824 | 0.0897 | 0.0171 | 1.53E-07 | 6.16E-05 | 27.51634 |
| rs35276016 | stroke | 6 | 24740789 | T | C | 0.0851 | 0.0984 | 0.0184 | 9.31E-08 | 6.40E-05 | 28.59912 |
| rs4151702 | stroke | 6 | 36645988 | C | G | 0.2 | -0.0551 | 0.0117 | 2.63E-06 | 4.96E-05 | 22.17837 |
| rs4886564 | stroke | 15 | 78530837 | T | C | 0.8701 | -0.0653 | 0.0138 | 2.37E-06 | 5.01E-05 | 22.39063 |
| rs475937 | stroke | 11 | 102687700 | A | C | 0.1318 | 0.0757 | 0.0137 | 2.92E-08 | 6.83E-05 | 30.53154 |
| rs34416434 | stroke | 16 | 73034906 | T | C | 0.0472 | -0.1155 | 0.0239 | 1.39E-06 | 5.23E-05 | 23.35427 |
| rs60102266 | stroke | 11 | 15427364 | T | C | 0.9435 | -0.0954 | 0.0201 | 2.09E-06 | 5.04E-05 | 22.52697 |
| rs2978551 | stroke | 8 | 62148943 | T | C | 0.339 | 0.0446 | 0.0096 | 3.39E-06 | 4.83E-05 | 21.58367 |
| rs57269940 | stroke | 2 | 206607700 | T | C | 0.2773 | 0.0499 | 0.0108 | 3.85E-06 | 4.78E-05 | 21.34773 |
| rs2060213 | stroke | 5 | 137957814 | T | C | 0.7556 | -0.0523 | 0.0108 | 1.28E-06 | 5.25E-05 | 23.4506 |
| rs12562305 | stroke | 1 | 60015280 | A | G | 0.0722 | 0.0882 | 0.0188 | 2.60E-06 | 4.93E-05 | 22.00997 |
| rs12630390 | stroke | 3 | 141030543 | A | G | 0.0407 | 0.1184 | 0.0256 | 3.72E-06 | 4.79E-05 | 21.39053 |
| rs11242678 | stroke | 6 | 1337180 | T | C | 0.2551 | 0.0643 | 0.0105 | 8.71E-10 | 8.39E-05 | 37.50088 |
| rs2585193 | stroke | 5 | 126641770 | A | G | 0.6842 | 0.0504 | 0.0098 | 2.45E-07 | 5.92E-05 | 26.44886 |
| rs2634074 | stroke | 4 | 111677041 | A | T | 0.7885 | -0.084 | 0.0112 | 6.56E-14 | 0.000126 | 56.24975 |
| rs10937513 | stroke | 3 | 191550504 | A | C | 0.061 | 0.0989 | 0.0197 | 5.02E-07 | 5.64E-05 | 25.20335 |
| rs635634 | stroke | 9 | 136155000 | T | C | 0.1907 | 0.0685 | 0.0126 | 6.03E-08 | 6.62E-05 | 29.55549 |
| rs147861947 | stroke | 9 | 112599050 | A | G | 0.9631 | 0.1342 | 0.0293 | 4.51E-06 | 4.70E-05 | 20.97818 |
| rs8103309 | stroke | 19 | 11174935 | T | C | 0.6554 | 0.0522 | 0.0103 | 3.70E-07 | 5.75E-05 | 25.68412 |
| rs9386182 | stroke | 6 | 147691069 | A | T | 0.4968 | -0.0467 | 0.0102 | 4.22E-06 | 4.69E-05 | 20.96194 |
| rs2066864 | stroke | 4 | 155525695 | A | G | 0.2455 | 0.0562 | 0.0106 | 1.29E-07 | 6.29E-05 | 28.10988 |
| rs11587860 | stroke | 1 | 156156951 | C | G | 0.3545 | -0.0689 | 0.0098 | 2.54E-12 | 0.000111 | 49.42929 |
| rs2284665 | stroke | 10 | 124226630 | T | G | 0.2145 | -0.0602 | 0.0111 | 5.99E-08 | 6.58E-05 | 29.41339 |
| rs10774624 | stroke | 12 | 111833788 | A | G | 0.5285 | -0.0654 | 0.0094 | 4.04E-12 | 0.000108 | 48.40585 |
| rs117426542 | stroke | 17 | 39815758 | A | G | 0.0309 | 0.1487 | 0.031 | 1.56E-06 | 5.15E-05 | 23.00894 |
| rs12445022 | stroke | 16 | 87575332 | A | G | 0.3346 | 0.052 | 0.0098 | 1.03E-07 | 6.30E-05 | 28.15481 |
| rs147076266 | stroke | 5 | 62723814 | A | G | 0.9845 | -0.2051 | 0.0443 | 3.56E-06 | 4.80E-05 | 21.43492 |
| rs7549874 | stroke | 1 | 203009634 | T | C | 0.2868 | 0.0495 | 0.0104 | 2.07E-06 | 5.07E-05 | 22.65384 |
| rs17035646 | stroke | 1 | 10796547 | A | G | 0.3514 | 0.0522 | 0.0096 | 6.12E-08 | 6.62E-05 | 29.56627 |
| rs10883926 | stroke | 10 | 105608838 | A | G | 0.6028 | 0.0503 | 0.0094 | 8.15E-08 | 6.41E-05 | 28.63376 |
| rs4942561 | Ischemic stroke | 13 | 47209347 | T | G | 0.759 | 0.0655 | 0.0116 | 1.77E-08 | 7.24E-05 | 31.8834 |
| rs2107595 | Ischemic stroke | 7 | 19049388 | A | G | 0.1673 | 0.0882 | 0.0132 | 2.33E-11 | 0.000101 | 44.64649 |
| rs473238 | Ischemic stroke | 11 | 102700360 | T | C | 0.1326 | 0.0831 | 0.0147 | 1.65E-08 | 7.26E-05 | 31.95696 |
| rs3184504 | Ischemic stroke | 12 | 111884608 | T | C | 0.4722 | 0.0779 | 0.0101 | 1.23E-14 | 0.000135 | 59.48811 |
| rs34311906 | Ischemic stroke | 4 | 113732090 | T | C | 0.5976 | -0.0649 | 0.0113 | 1.07E-08 | 7.49E-05 | 32.98607 |
| rs2758612 | Ischemic stroke | 1 | 156205301 | T | C | 0.6453 | 0.0653 | 0.0111 | 3.68E-09 | 7.86E-05 | 34.60815 |
| rs11242678 | Ischemic stroke | 6 | 1337180 | T | C | 0.255 | 0.0723 | 0.0114 | 2.70E-10 | 9.13E-05 | 40.22212 |
| rs2634074 | Ischemic stroke | 4 | 111677041 | A | T | 0.7877 | -0.0941 | 0.0121 | 5.91E-15 | 0.000137 | 60.47927 |
| rs635634 | Ischemic stroke | 9 | 136155000 | T | C | 0.1921 | 0.0772 | 0.0134 | 9.18E-09 | 7.54E-05 | 33.19121 |
| rs2066864 | Ischemic stroke | 4 | 155525695 | A | G | 0.2452 | 0.0634 | 0.0115 | 3.51E-08 | 6.90E-05 | 30.39351 |
| rs2107595 | Ischemic stroke-LAS | 7 | 19049388 | A | G | 0.1677 | 0.0319 | 0.2358 | 1.44E-13 | 0.000133 | 54.63917 |
| rs10820405 | Ischemic stroke-LAS | 9 | 106010237 | A | G | 0.1847 | 0.0331 | -0.181 | 4.51E-08 | 7.30E-05 | 29.96804 |
| rs476762 | Ischemic stroke-LAS | 11 | 102710707 | A | T | 0.133 | 0.0353 | 0.2010 | 1.22E-08 | 7.90E-05 | 32.42206 |
| rs35818742 | Ischemic stroke-SVS | 10 | 115793989 | T | C | 0.8656 | -0.1674 | 0.0329 | 3.65E-07 | 0.000131 | 25.88897 |
| rs149163995 | Ischemic stroke-SVS | 2 | 203777226 | T | C | 0.1256 | -0.1945 | 0.0368 | 1.22E-07 | 0.000141 | 27.93439 |
| rs7766042 | Ischemic stroke-SVS | 6 | 1366718 | T | C | 0.8984 | -0.2129 | 0.0397 | 7.97E-08 | 0.000145 | 28.75848 |
| rs76576182 | Ischemic stroke-SVS | 7 | 96656620 | A | G | 0.9788 | -0.4436 | 0.0844 | 1.46E-07 | 0.000139 | 27.62446 |
| rs12445022 | Ischemic stroke-SVS | 16 | 87575332 | A | G | 0.3367 | 0.1301 | 0.0244 | 9.26E-08 | 0.000144 | 28.42959 |
| rs146390073 | Ischemic stroke-CES | 1 | 2.41E+08 | T | C | 0.0215 | 0.6688 | 0.1195 | 2.2E-08 | 7.58E-05 | 31.322 |
| rs2466455 | Ischemic stroke-CES | 4 | 1.12E+08 | T | C | 0.7826 | -0.2992 | 0.0222 | 2.75E-41 | 0.000439296 | 181.642 |
| rs6838973 | Ischemic stroke-CES | 4 | 1.12E+08 | T | C | 0.4341 | -0.1079 | 0.0196 | 3.58E-08 | 7.33E-05 | 30.306 |
| rs12932445 | Ischemic stroke-CES | 16 | 73069888 | C | T | 0.8195 | -0.1758 | 0.0245 | 6.88E-13 | 0.000124561 | 51.488 |
| rs16838082 | Subarachnoid hemorrhage | 1 | 1.95E+08 | G | A | 0.3588 | 0.1911 | 0.0413 | 3.70E-06 | 0.000106 | 21.41002 |
| rs792108 | Subarachnoid hemorrhage | 2 | 5532793 | C | T | 0.5901 | 0.2011 | 0.0404 | 6.27E-07 | 0.000122 | 24.77748 |
| rs188736573 | Subarachnoid hemorrhage | 6 | 1.4E+08 | T | C | 0.03705 | 0.5187 | 0.1084 | 1.71E-06 | 0.000113 | 22.89653 |
| rs2673593 | Subarachnoid hemorrhage | 8 | 1.33E+08 | G | T | 0.3706 | 0.1935 | 0.0411 | 2.49E-06 | 0.000109 | 22.16532 |
| rs2505256 | Subarachnoid hemorrhage | 10 | 38369818 | T | A | 0.3319 | 0.2504 | 0.0533 | 2.57E-06 | 0.000109 | 22.07039 |
| rs16930998 | Subarachnoid hemorrhage | 11 | 5462702 | A | G | 0.01178 | 0.9366 | 0.1976 | 2.14E-06 | 0.000111 | 22.46623 |
| rs142664188 | Subarachnoid hemorrhage | 12 | 1.23E+08 | G | C | 0.004516 | 1.7156 | 0.3529 | 1.17E-06 | 0.000117 | 23.63331 |
| rs11609982 | Subarachnoid hemorrhage | 12 | 1.08E+08 | A | G | 0.3344 | 0.1989 | 0.0421 | 2.35E-06 | 0.00011 | 22.32035 |
| rs6602891 | Subarachnoid hemorrhage | 13 | 1.15E+08 | T | G | 0.4052 | 0.1902 | 0.0405 | 2.71E-06 | 0.000109 | 22.05498 |
| rs10163011 | Subarachnoid hemorrhage | 15 | 93760024 | C | T | 0.9792 | 0.6811 | 0.1476 | 3.94E-06 | 0.000105 | 21.29339 |
| rs2738814 | Subarachnoid hemorrhage | 16 | 75516282 | A | C | 0.6363 | 0.2003 | 0.0412 | 1.13E-06 | 0.000117 | 23.63541 |

CES, cardioembolic; Chr, chromosome; EAF, Effect allele frequency; LAS, large artery atherosclerosis; Pos, position; SE, standard error; SNP, single nucleotide polymorphism; SVS, small-vessel. The threshold was set at *P* < 1×10^-6^ for nontraumatic intracranial hemorrhage and subarachnoid hemorrhage.

Characteristics of selected SNPs for neurodegenerative diseases.

| **SNP** | **Trait** | **Chr** | **Pos.** | **Effect allele** | **Other allele** | **EAF** | **Beta** | **SE** | ***P value*** | **R^2^** | **F statistic** |
| --- | --- | --- | --- | --- | --- | --- | --- | --- | --- | --- | --- |
| rs679515 | AD | 1 | 207750568 | C | T | NA | -0.1508 | 0.0183 | 1.55E-16 | 0.001061 | 67.9048 |
| rs7584040 | AD | 2 | 127863224 | T | C | NA | 0.0862 | 0.0172 | 5.34E-07 | 0.000393 | 25.11641 |
| rs10933431 | AD | 2 | 233981912 | C | G | NA | 0.1001 | 0.0194 | 2.55E-07 | 0.000416 | 26.62347 |
| rs6733839 | AD | 2 | 127892810 | T | C | NA | 0.1693 | 0.0154 | 4.02E-28 | 0.001887 | 120.8572 |
| rs35695568 | AD | 2 | 186794162 | T | G | NA | 0.1152 | 0.0247 | 3.20E-06 | 0.00034 | 21.75259 |
| rs7618668 | AD | 3 | 45097509 | A | G | NA | -0.1297 | 0.0258 | 4.95E-07 | 0.000395 | 25.27205 |
| rs28660482 | AD | 4 | 66245059 | A | T | NA | 0.222 | 0.0475 | 2.90E-06 | 0.000342 | 21.84332 |
| rs11168036 | AD | 5 | 139707439 | G | T | NA | -0.0754 | 0.0143 | 1.43E-07 | 0.000435 | 27.80165 |
| rs9381563 | AD | 6 | 47432637 | T | C | NA | -0.0821 | 0.0148 | 2.93E-08 | 0.000481 | 30.77251 |
| rs114812713 | AD | 6 | 41034000 | C | G | NA | 0.298 | 0.0431 | 4.47E-12 | 0.000747 | 47.80551 |
| rs34665982 | AD | 6 | 32560306 | C | T | NA | -0.0967 | 0.0166 | 5.80E-09 | 0.000531 | 33.93413 |
| rs117240937 | AD | 7 | 127426090 | A | G | NA | -0.3122 | 0.0672 | 3.35E-06 | 0.000338 | 21.58377 |
| rs11767557 | AD | 7 | 143109139 | C | T | NA | -0.1028 | 0.0182 | 1.56E-08 | 0.000499 | 31.90388 |
| rs143429938 | AD | 7 | 33721795 | T | C | NA | 0.3535 | 0.0769 | 4.25E-06 | 0.00033 | 21.1313 |
| rs9649710 | AD | 7 | 50322832 | G | A | NA | 0.0676 | 0.0148 | 4.79E-06 | 0.000326 | 20.86267 |
| rs73223431 | AD | 8 | 27219987 | T | C | NA | 0.0936 | 0.0153 | 8.34E-10 | 0.000585 | 37.42561 |
| rs13252043 | AD | 8 | 71551628 | T | C | NA | 0.114 | 0.0237 | 1.57E-06 | 0.000362 | 23.13732 |
| rs867230 | AD | 8 | 27468503 | A | C | NA | 0.1333 | 0.0158 | 3.49E-17 | 0.001112 | 71.17806 |
| rs6559689 | AD | 9 | 85450616 | T | C | NA | 0.1585 | 0.0335 | 2.17E-06 | 0.00035 | 22.38561 |
| rs11257242 | AD | 10 | 11721119 | G | C | NA | 0.0841 | 0.0154 | 4.64E-08 | 0.000466 | 29.82295 |
| rs9787911 | AD | 11 | 131769402 | C | T | NA | 0.0662 | 0.0144 | 4.39E-06 | 0.000331 | 21.13445 |
| rs3740688 | AD | 11 | 47380340 | T | G | NA | 0.0935 | 0.0144 | 9.70E-11 | 0.000659 | 42.15977 |
| rs3851179 | AD | 11 | 85868640 | C | T | NA | 0.1198 | 0.0148 | 5.81E-16 | 0.001024 | 65.52246 |
| rs1582763 | AD | 11 | 60021948 | A | G | NA | -0.1232 | 0.0149 | 1.19E-16 | 0.001068 | 68.36737 |
| rs72993825 | AD | 11 | 112559343 | T | C | NA | -0.1763 | 0.036 | 9.86E-07 | 0.000375 | 23.98279 |
| rs117394726 | AD | 12 | 127222883 | C | A | NA | 0.2193 | 0.0465 | 2.46E-06 | 0.000348 | 22.24187 |
| rs17125924 | AD | 14 | 53391680 | G | A | NA | 0.1222 | 0.0246 | 6.62E-07 | 0.000386 | 24.67585 |
| rs12590654 | AD | 14 | 92938855 | A | G | NA | -0.0906 | 0.0157 | 8.73E-09 | 0.000521 | 33.30099 |
| rs383902 | AD | 15 | 59034174 | T | C | NA | -0.0698 | 0.0151 | 3.81E-06 | 0.000334 | 21.36766 |
| rs34971488 | AD | 16 | 81779775 | A | G | NA | 0.094 | 0.0198 | 2.07E-06 | 0.000352 | 22.53852 |
| rs28588186 | AD | 16 | 19910313 | G | C | NA | -0.088 | 0.0181 | 1.12E-06 | 0.00037 | 23.63786 |
| rs2632516 | AD | 17 | 56409089 | C | G | NA | -0.0748 | 0.0147 | 3.67E-07 | 0.000405 | 25.89217 |
| rs12151021 | AD | 19 | 1050874 | G | A | NA | -0.1071 | 0.0169 | 2.56E-10 | 0.000628 | 40.16109 |
| rs150685845 | AD | 19 | 45675180 | G | A | NA | 0.5561 | 0.0645 | 6.62E-18 | 0.001161 | 74.3338 |
| rs1081105 | AD | 19 | 45412955 | C | A | NA | 0.942 | 0.0436 | ####### | 0.007249 | 466.7978 |
| rs72654445 | AD | 19 | 45417200 | A | G | NA | -0.5425 | 0.0811 | 2.27E-11 | 0.0007 | 44.74637 |
| rs147711004 | AD | 19 | 45337918 | A | G | NA | 1.1354 | 0.0366 | ####### | 0.014831 | 962.3557 |
| rs141739979 | AD | 19 | 45374983 | T | G | NA | -0.4544 | 0.0855 | 1.07E-07 | 0.000442 | 28.24518 |
| rs7412 | AD | 19 | 45412079 | T | C | NA | -0.4673 | 0.0305 | 6.40E-53 | 0.003659 | 234.7426 |
| rs111278137 | AD | 19 | 45215081 | A | G | NA | -0.4735 | 0.0713 | 3.20E-11 | 0.000689 | 44.10227 |
| rs118004808 | AD | 19 | 45439498 | T | C | NA | -0.5523 | 0.1015 | 5.27E-08 | 0.000463 | 29.60861 |
| rs8111708 | AD | 19 | 18558876 | G | A | NA | 0.0696 | 0.0151 | 3.95E-06 | 0.000332 | 21.24538 |
| rs6014724 | AD | 20 | 54998544 | G | A | NA | -0.1319 | 0.0259 | 3.65E-07 | 0.000406 | 25.93523 |
| rs2830489 | AD | 21 | 28148191 | T | C | NA | -0.0837 | 0.0162 | 2.42E-07 | 0.000417 | 26.69444 |
| rs35749011 | PD | 1 | 155135036 | A | G | 0.0191 | 0.7508 | 0.0659 | 5.02E-30 | 0.000269 | 129.8004 |
| rs823106 | PD | 1 | 205656453 | C | G | 0.8488 | -0.1492 | 0.0239 | 4.10E-10 | 8.07E-05 | 38.97086 |
| rs4613239 | PD | 2 | 169119609 | G | C | 0.1326 | 0.1784 | 0.0248 | 6.21E-13 | 0.000107 | 51.74692 |
| rs6741007 | PD | 2 | 135537119 | G | T | 0.4507 | -0.1233 | 0.0175 | 2.09E-12 | 0.000103 | 49.64188 |
| rs4488803 | PD | 3 | 58218352 | A | G | 0.3746 | -0.1136 | 0.0199 | 1.08E-08 | 6.75E-05 | 32.58732 |
| rs10513789 | PD | 3 | 182760073 | G | T | 0.1826 | -0.1596 | 0.0219 | 3.18E-13 | 0.00011 | 53.10993 |
| rs34311866 | PD | 4 | 951947 | C | T | 0.1958 | 0.2272 | 0.0231 | 7.97E-23 | 0.0002 | 96.73662 |
| rs4698412 | PD | 4 | 15737348 | A | G | 0.553 | 0.1258 | 0.0168 | 7.05E-14 | 0.000116 | 56.07134 |
| rs356203 | PD | 4 | 90666041 | T | C | 0.6169 | -0.2398 | 0.0178 | 3.01E-41 | 0.000376 | 181.4916 |
| rs75646569 | PD | 5 | 60345424 | G | T | 0.1117 | 0.1916 | 0.0266 | 5.62E-13 | 0.000107 | 51.8831 |
| rs35265698 | PD | 6 | 32561334 | G | C | 0.1547 | -0.2 | 0.0303 | 3.93E-11 | 9.06E-05 | 43.56853 |
| rs858295 | PD | 7 | 23245569 | G | A | 0.3947 | -0.1039 | 0.0176 | 3.83E-09 | 7.22E-05 | 34.85009 |
| rs620490 | PD | 8 | 16697579 | G | T | 0.2762 | -0.1174 | 0.019 | 6.46E-10 | 7.91E-05 | 38.17923 |
| rs144814361 | PD | 10 | 121410917 | T | C | 0.0174 | 0.4411 | 0.068 | 9.07E-11 | 8.72E-05 | 42.07794 |
| rs329647 | PD | 11 | 133764666 | C | G | 0.6662 | -0.1133 | 0.0178 | 1.94E-10 | 8.39E-05 | 40.5152 |
| rs75505347 | PD | 12 | 40885549 | T | C | 0.0195 | 0.3917 | 0.0674 | 6.12E-09 | 7.00E-05 | 33.77424 |
| rs10847864 | PD | 12 | 123326598 | T | G | 0.3625 | 0.1274 | 0.0179 | 9.81E-13 | 0.000105 | 50.65601 |
| rs4774417 | PD | 15 | 61993702 | A | G | 0.7397 | 0.1052 | 0.0192 | 4.63E-08 | 6.22E-05 | 30.02114 |
| rs12934900 | PD | 16 | 30923602 | T | A | 0.6571 | 0.1215 | 0.0184 | 4.33E-11 | 9.03E-05 | 43.60287 |
| rs58879558 | PD | 17 | 44095467 | C | T | 0.2229 | -0.2383 | 0.025 | 1.36E-21 | 0.000188 | 90.85865 |
| rs10451230 | PD | 17 | 16035225 | T | A | 0.565 | -0.096 | 0.0175 | 4.42E-08 | 6.23E-05 | 30.09294 |
| rs4588066 | PD | 18 | 40672964 | A | G | 0.326 | 0.1046 | 0.0178 | 4.45E-09 | 7.15E-05 | 34.53199 |
| rs10463311 | ALS | 5 | 150410835 | T | C | NA | -0.0897 | 0.0149 | 1.61E-09 | 0.000428 | 36.24202 |
| rs3849943 | ALS | 9 | 27543382 | T | C | NA | -0.1718 | 0.0153 | 3.91E-29 | 0.001487 | 126.085 |
| rs58854276 | ALS | 10 | 114145044 | G | A | NA | -0.0796 | 0.0138 | 8.26E-09 | 0.000393 | 33.27116 |
| rs74654358 | ALS | 12 | 64881967 | A | G | NA | 0.1976 | 0.0337 | 4.53E-09 | 0.000406 | 34.38065 |
| rs142321490 | ALS | 12 | 58676132 | C | G | NA | 0.3172 | 0.0513 | 6.28E-10 | 0.000451 | 38.23241 |
| rs12973192 | ALS | 19 | 17753239 | G | C | NA | 0.1205 | 0.0153 | 3.39E-15 | 0.000732 | 62.02849 |
| rs75087725 | ALS | 21 | 45753117 | A | C | NA | 0.5145 | 0.0672 | 1.91E-14 | 0.000692 | 58.61816 |

Characteristics of selected SNPs for migraine.

| **SNP** | **Trait** | **Chr** | **Pos.** | **Effect allele** | **Other allele** | **EAF** | **Beta** | **SE** | ***P value*** | **R^2^** | **F statistic** |
| --- | --- | --- | --- | --- | --- | --- | --- | --- | --- | --- | --- |
| rs9349379 | Migraine | 6 | 12903725 | G | A | 0.45246 | -0.0786553 | 0.0110271 | 9.83E-13 | 0.000166071 | 50.87805753 |
| rs9266329 | Migraine | 6 | 31363011 | A | G | 0.254138 | -0.0771558 | 0.0126798 | 1.17E-09 | 0.000120863 | 37.02623173 |
| rs7398375 | Migraine | 12 | 57147065 | G | C | 0.331229 | 0.0726386 | 0.0117012 | 5.37E-10 | 0.000125792 | 38.53641319 |
| rs62145958 | Migraine with aura | 2 | 68199770 | C | A | 0.525965 | 0.0759184 | 0.0163408 | 3.39E-06 | 7.41E-05 | 21.58462377 |
| rs207882 | Migraine with aura | 2 | 216141991 | T | G | 0.406783 | -0.0793563 | 0.0166931 | 2.00E-06 | 7.76E-05 | 22.59882453 |
| rs17622145 | Migraine with aura | 3 | 30241357 | A | T | 0.122996 | -0.130184 | 0.0256998 | 4.07E-07 | 8.81E-05 | 25.65976973 |
| rs4683465 | Migraine with aura | 3 | 139982653 | C | T | 0.323965 | 0.0849676 | 0.0172248 | 8.10E-07 | 8.35E-05 | 24.33298261 |
| rs3845979 | Migraine with aura | 3 | 157793859 | G | A | 0.477434 | 0.0745592 | 0.0163176 | 4.89E-06 | 7.16E-05 | 20.87790573 |
| rs6850727 | Migraine with aura | 4 | 1329485 | C | G | 0.155461 | -0.120548 | 0.0230207 | 1.64E-07 | 9.41E-05 | 27.42079155 |
| rs2517653 | Migraine with aura | 6 | 30153408 | A | G | 0.343077 | -0.101953 | 0.0173038 | 3.82E-09 | 0.000119127 | 34.7147518 |
| rs2442752 | Migraine with aura | 6 | 31383987 | C | T | 0.345271 | 0.0869531 | 0.0170079 | 3.18E-07 | 8.97E-05 | 26.13760384 |
| rs79003543 | Migraine with aura | 7 | 2959834 | C | G | 0.0626686 | 0.153187 | 0.0328569 | 3.13E-06 | 7.46E-05 | 21.73640259 |
| rs117263825 | Migraine with aura | 7 | 71441628 | T | C | 0.0957137 | 0.12389 | 0.0266507 | 3.34E-06 | 7.42E-05 | 21.60987644 |
| rs4879993 | Migraine with aura | 9 | 36473562 | T | C | 0.663735 | 0.0821498 | 0.0173793 | 2.28E-06 | 7.67E-05 | 22.3432055 |
| rs7096340 | Migraine with aura | 10 | 15232214 | C | G | 0.929203 | 0.17876 | 0.0335355 | 9.80E-08 | 9.75E-05 | 28.4137228 |
| rs7894090 | Migraine with aura | 10 | 18002359 | A | G | 0.876954 | 0.119544 | 0.0255552 | 2.90E-06 | 7.51E-05 | 21.88235289 |
| rs113849028 | Migraine with aura | 10 | 58528292 | A | T | 0.0268851 | -0.263289 | 0.0537968 | 9.87E-07 | 8.22E-05 | 23.95242906 |
| rs8002734 | Migraine with aura | 13 | 99842713 | A | T | 0.228805 | 0.0964395 | 0.0193801 | 6.48E-07 | 8.50E-05 | 24.762523 |
| rs12430402 | Migraine with aura | 13 | 107537435 | G | A | 0.116132 | 0.115046 | 0.0245683 | 2.83E-06 | 7.52E-05 | 21.92753701 |
| rs117208805 | Migraine with aura | 15 | 48693929 | A | T | 0.0691097 | -0.15771 | 0.0338157 | 3.10E-06 | 7.46E-05 | 21.75097447 |
| rs16035 | Migraine with aura | 19 | 13245336 | G | T | 0.503033 | -0.0918824 | 0.0163146 | 1.78E-08 | 0.000108845 | 31.71822092 |
| rs34897924 | Migraine with aura | 19 | 13739021 | G | A | 0.201164 | 0.0918628 | 0.0199643 | 4.20E-06 | 7.27E-05 | 21.17230793 |
| rs12461522 | Migraine with aura | 19 | 52496138 | T | C | 0.0704368 | 0.148559 | 0.0307769 | 1.39E-06 | 8.00E-05 | 23.29942519 |
| rs117750534 | Migraine with aura | 22 | 28416716 | T | A | 0.0276498 | -0.250798 | 0.0543166 | 3.89E-06 | 7.32E-05 | 21.31964666 |
| rs9349379 | Migraine without aura | 6 | 12903725 | G | A | 0.453151 | -0.0981548 | 0.0178181 | 3.61E-08 | 0.000102595 | 30.34571426 |
| rs9486725 | Migraine without aura | 6 | 96613283 | T | C | 0.298277 | 0.110915 | 0.0190268 | 5.56E-09 | 0.000114886 | 33.98178 |
| rs10774231 | Migraine without aura | 12 | 4406208 | C | T | 0.551576 | -0.097881 | 0.0177544 | 3.53E-08 | 0.000102757 | 30.39358003 |
| rs7398375 | Migraine without aura | 12 | 57147065 | G | C | 0.330836 | 0.105129 | 0.0188123 | 2.29E-08 | 0.000105581 | 31.22901873 |
| rs10968014 | Migraine without aura，drug-induced | 9 | 27621486 | C | G | 0.247351 | -0.39289 | 0.093618 | 2.71E-05 | 4.67E-05 | 17.61286 |
| rs11155500 | Migraine without aura，drug-induced | 6 | 1.48E+08 | C | G | 0.000344 | 3.51751 | 0.849632 | 3.47E-05 | 4.54E-05 | 17.13984 |
| rs112106670 | Migraine without aura，drug-induced | 17 | 65475436 | A | G | 0.001585 | 2.36164 | 0.520088 | 5.60E-06 | 5.46E-05 | 20.61918 |
| rs112527609 | Migraine without aura，drug-induced | 10 | 99951238 | A | T | 0.066497 | 0.521972 | 0.127353 | 4.16E-05 | 4.45E-05 | 16.79863 |
| rs113321921 | Migraine without aura，drug-induced | 18 | 38506985 | A | G | 0.001667 | 2.26689 | 0.499722 | 5.73E-06 | 5.45E-05 | 20.57793 |
| rs114316363 | Migraine without aura，drug-induced | 5 | 1.26E+08 | A | G | 0.080896 | -0.70808 | 0.173963 | 4.69E-05 | 4.39E-05 | 16.56737 |
| rs114982480 | Migraine without aura，drug-induced | 3 | 1.46E+08 | A | C | 0.006375 | 1.36616 | 0.31184 | 1.18E-05 | 5.09E-05 | 19.19275 |
| rs115640110 | Migraine without aura，drug-induced | 2 | 181840 | T | G | 0.004564 | 1.42899 | 0.349945 | 4.44E-05 | 4.42E-05 | 16.67464 |
| rs117283049 | Migraine without aura，drug-induced | 11 | 7669396 | G | T | 0.055837 | 0.56079 | 0.138031 | 4.85E-05 | 4.37E-05 | 16.50612 |
| rs117843878 | Migraine without aura，drug-induced | 8 | 1.09E+08 | T | C | 0.012818 | 1.03809 | 0.241268 | 1.69E-05 | 4.91E-05 | 18.51264 |
| rs11901669 | Migraine without aura，drug-induced | 2 | 34433096 | A | C | 0.306297 | 0.322083 | 0.079378 | 4.96E-05 | 4.36E-05 | 16.46374 |
| rs12199099 | Migraine without aura，drug-induced | 6 | 98397109 | G | C | 0.024324 | 0.858181 | 0.187911 | 4.95E-06 | 5.53E-05 | 20.85696 |
| rs13047841 | Migraine without aura，drug-induced | 21 | 38504635 | T | C | 0.022942 | 0.822854 | 0.192664 | 1.95E-05 | 4.83E-05 | 18.24073 |
| rs143274540 | Migraine without aura，drug-induced | 5 | 23615103 | T | G | 0.035065 | 0.713645 | 0.163429 | 1.26E-05 | 5.05E-05 | 19.06795 |
| rs144229664 | Migraine without aura，drug-induced | 16 | 82960643 | C | T | 0.001568 | 1.96204 | 0.482466 | 4.77E-05 | 4.38E-05 | 16.53789 |
| rs144498959 | Migraine without aura，drug-induced | 6 | 3710667 | A | G | 0.01357 | 1.04734 | 0.233176 | 7.07E-06 | 5.35E-05 | 20.17462 |
| rs145744292 | Migraine without aura，drug-induced | 6 | 1.34E+08 | G | T | 0.044236 | 0.640563 | 0.156526 | 4.27E-05 | 4.44E-05 | 16.74744 |
| rs146980396 | Migraine without aura，drug-induced | 4 | 66358809 | T | C | 0.029035 | -1.49553 | 0.365551 | 4.29E-05 | 4.44E-05 | 16.73755 |
| rs149723129 | Migraine without aura，drug-induced | 11 | 26847213 | C | G | 0.015484 | 0.990092 | 0.236562 | 2.85E-05 | 4.64E-05 | 17.51696 |
| rs1564897 | Migraine without aura，drug-induced | 4 | 95510517 | T | C | 0.090595 | 0.484307 | 0.119057 | 4.74E-05 | 4.39E-05 | 16.54738 |
| rs185602011 | Migraine without aura，drug-induced | 12 | 1.22E+08 | A | G | 0.026897 | 0.818329 | 0.188675 | 1.44E-05 | 4.99E-05 | 18.81156 |
| rs1896374 | Migraine without aura，drug-induced | 10 | 1.24E+08 | T | C | 0.951333 | 0.960772 | 0.234242 | 4.10E-05 | 4.46E-05 | 16.82321 |
| rs2439657 | Migraine without aura，drug-induced | 5 | 3216651 | T | C | 0.896047 | -0.47186 | 0.10931 | 1.58E-05 | 4.94E-05 | 18.63368 |
| rs2827043 | Migraine without aura，drug-induced | 21 | 21799032 | C | T | 0.643143 | -0.36709 | 0.076205 | 1.46E-06 | 6.15E-05 | 23.20419 |
| rs34852471 | Migraine without aura，drug-induced | 4 | 1.48E+08 | A | G | 0.054607 | 0.608959 | 0.145776 | 2.95E-05 | 4.63E-05 | 17.45025 |
| rs373520465 | Migraine without aura，drug-induced | 15 | 45268505 | T | A | 0.031493 | -1.29247 | 0.312619 | 3.56E-05 | 4.53E-05 | 17.09259 |
| rs10501644 | Migraine with aura, drug-induced | 11 | 87736320 | C | A | 0.13987 | 0.421472 | 0.101342 | 3.20E-05 | 4.58E-05 | 17.29642 |
| rs10770131 | Migraine with aura, drug-induced | 11 | 10660701 | C | T | 0.5171 | 0.339544 | 0.078074 | 1.37E-05 | 5.01E-05 | 18.91372 |
| rs10908491 | Migraine with aura, drug-induced | 1 | 1.56E+08 | G | A | 0.461925 | 0.33667 | 0.0777 | 1.47E-05 | 4.98E-05 | 18.77414 |
| rs11660253 | Migraine with aura, drug-induced | 18 | 69800396 | C | T | 0.056696 | -0.90972 | 0.217587 | 2.90E-05 | 4.63E-05 | 17.48027 |
| rs116928228 | Migraine with aura, drug-induced | 13 | 25324282 | C | T | 0.018065 | 0.904442 | 0.21312 | 2.20E-05 | 4.77E-05 | 18.00988 |
| rs117984195 | Migraine with aura, drug-induced | 8 | 1.25E+08 | A | G | 0.012445 | 1.17423 | 0.271739 | 1.55E-05 | 4.95E-05 | 18.6724 |
| rs12440761 | Migraine with aura, drug-induced | 15 | 97239200 | C | T | 0.58122 | 0.330016 | 0.080872 | 4.49E-05 | 4.41E-05 | 16.65231 |
| rs12517527 | Migraine with aura, drug-induced | 5 | 1.06E+08 | C | A | 0.649507 | 0.368157 | 0.08431 | 1.26E-05 | 5.05E-05 | 19.06807 |
| rs140518488 | Migraine with aura, drug-induced | 3 | 1.72E+08 | T | C | 0.078333 | -0.75201 | 0.180614 | 3.13E-05 | 4.59E-05 | 17.33582 |
| rs16925679 | Migraine with aura, drug-induced | 9 | 10277602 | T | C | 0.240776 | 0.356609 | 0.085358 | 2.94E-05 | 4.63E-05 | 17.45412 |
| rs17651697 | Migraine with aura, drug-induced | 5 | 76311794 | T | C | 0.056519 | 0.582693 | 0.143409 | 4.84E-05 | 4.38E-05 | 16.50914 |
| rs188128204 | Migraine with aura, drug-induced | 7 | 6334020 | A | G | 0.007116 | 1.33641 | 0.323108 | 3.53E-05 | 4.53E-05 | 17.10731 |
| rs1893373 | Migraine with aura, drug-induced | 18 | 37541882 | T | A | 0.471618 | -0.31951 | 0.078263 | 4.46E-05 | 4.42E-05 | 16.66665 |
| rs2133108 | Migraine with aura, drug-induced | 1 | 81724426 | C | A | 0.079381 | 0.554243 | 0.126657 | 1.21E-05 | 5.08E-05 | 19.14872 |
| rs255171 | Migraine with aura, drug-induced | 7 | 30735038 | T | C | 0.545741 | -0.38934 | 0.077112 | 4.44E-07 | 6.76E-05 | 25.49246 |
| rs2593955 | Migraine with aura, drug-induced | 3 | 1.09E+08 | G | A | 0.122348 | -0.57802 | 0.13701 | 2.46E-05 | 4.72E-05 | 17.79813 |
| rs2644671 | Migraine with aura, drug-induced | 5 | 1.76E+08 | C | T | 0.277394 | 0.358285 | 0.08229 | 1.34E-05 | 5.02E-05 | 18.95662 |
| rs3095695 | Migraine with aura, drug-induced | 5 | 1.58E+08 | C | T | 0.268797 | 0.338107 | 0.082903 | 4.54E-05 | 4.41E-05 | 16.63301 |
| rs35286716 | Migraine with aura, drug-induced | 16 | 25224265 | T | C | 0.186506 | 0.435577 | 0.091083 | 1.73E-06 | 6.06E-05 | 22.86925 |
| rs4721648 | Migraine with aura, drug-induced | 7 | 17683486 | A | G | 0.682623 | 0.374586 | 0.08737 | 1.81E-05 | 4.87E-05 | 18.38125 |
| rs4842862 | Migraine with aura, drug-induced | 15 | 84950972 | G | A | 0.826249 | -0.39725 | 0.095456 | 3.16E-05 | 4.59E-05 | 17.31869 |
| rs534008681 | Migraine with aura, drug-induced | 5 | 1.51E+08 | G | C | 0.007132 | 1.28743 | 0.307833 | 2.89E-05 | 4.64E-05 | 17.491 |
| rs551994 | Migraine with aura, drug-induced | 7 | 81170852 | T | A | 0.967864 | 1.29187 | 0.312372 | 3.54E-05 | 4.53E-05 | 17.10374 |
| rs60008671 | Migraine with aura, drug-induced | 8 | 20240827 | T | C | 0.349329 | 0.329907 | 0.079554 | 3.37E-05 | 4.56E-05 | 17.19729 |
| rs62087003 | Migraine with aura, drug-induced | 18 | 29444425 | G | A | 0.004564 | 1.41755 | 0.335509 | 2.39E-05 | 4.73E-05 | 17.85115 |
| rs62475282 | Migraine with aura, drug-induced | 7 | 1.57E+08 | T | C | 0.277265 | -0.41047 | 0.094045 | 1.27E-05 | 5.05E-05 | 19.04936 |
| rs62545470 | Migraine with aura, drug-induced | 9 | 70576762 | T | C | 0.230077 | -0.44051 | 0.101206 | 1.35E-05 | 5.02E-05 | 18.94483 |
| rs71563798 | Migraine with aura, drug-induced | 6 | 1.58E+08 | A | G | 0.087397 | 0.500867 | 0.120584 | 3.27E-05 | 4.57E-05 | 17.25294 |
| rs72673103 | Migraine with aura, drug-induced | 14 | 26686411 | A | G | 0.042612 | 0.68866 | 0.166221 | 3.43E-05 | 4.55E-05 | 17.16468 |
| rs7276625 | Migraine with aura, drug-induced | 21 | 24005594 | G | T | 0.447112 | -0.3531 | 0.079026 | 7.89E-06 | 5.29E-05 | 19.96432 |
| rs72792970 | Migraine with aura, drug-induced | 2 | 28525971 | C | T | 0.045756 | -1.13315 | 0.272056 | 3.11E-05 | 4.60E-05 | 17.34826 |
| rs72951395 | Migraine with aura, drug-induced | 11 | 37198445 | G | A | 0.025719 | 0.834317 | 0.204203 | 4.39E-05 | 4.42E-05 | 16.69305 |
| rs73102652 | Migraine with aura, drug-induced | 20 | 31961536 | A | G | 0.03123 | 0.741657 | 0.175924 | 2.49E-05 | 4.71E-05 | 17.77271 |
| rs75236701 | Migraine with aura, drug-induced | 12 | 1.03E+08 | A | G | 0.009405 | 1.1683 | 0.277406 | 2.54E-05 | 4.70E-05 | 17.73678 |
| rs7613349 | Migraine with aura, drug-induced | 3 | 18819722 | G | A | 0.021933 | 0.826297 | 0.199547 | 3.46E-05 | 4.54E-05 | 17.14666 |
| rs76476131 | Migraine with aura, drug-induced | 19 | 6304401 | C | T | 0.032566 | -1.44397 | 0.329422 | 1.17E-05 | 5.09E-05 | 19.2136 |
| rs77076221 | Migraine with aura, drug-induced | 6 | 94369198 | T | C | 0.020669 | 0.844884 | 0.205447 | 3.92E-05 | 4.48E-05 | 16.91189 |
| rs77154317 | Migraine with aura, drug-induced | 4 | 1.25E+08 | A | G | 0.031783 | 0.773937 | 0.177753 | 1.34E-05 | 5.02E-05 | 18.95724 |
| rs7763653 | Migraine with aura, drug-induced | 6 | 1505945 | A | G | 0.355937 | 0.34969 | 0.078872 | 9.27E-06 | 5.21E-05 | 19.657 |
| rs7895693 | Migraine with aura, drug-induced | 10 | 60859069 | C | T | 0.645181 | -0.34108 | 0.079128 | 1.63E-05 | 4.92E-05 | 18.57954 |
| rs936730 | Migraine with aura, drug-induced | 17 | 6304404 | C | T | 0.659937 | -0.32523 | 0.079249 | 4.06E-05 | 4.46E-05 | 16.84122 |
| rs953471 | Migraine with aura, drug-induced | 9 | 1.24E+08 | A | G | 0.047234 | 0.621129 | 0.15215 | 4.46E-05 | 4.42E-05 | 16.66546 |
| rs9574711 | Migraine with aura, drug-induced | 13 | 80674795 | C | T | 0.226318 | 0.37086 | 0.086198 | 1.69E-05 | 4.91E-05 | 18.51089 |
| rs986320 | Migraine with aura, drug-induced | 10 | 9532590 | T | C | 0.000694 | 2.40934 | 0.568318 | 2.24E-05 | 4.76E-05 | 17.97261 |
| rs10501644 | Migraine with aura, drug-induced | 11 | 87736320 | C | A | 0.13987 | 0.421472 | 0.101342 | 3.20E-05 | 4.58E-05 | 17.29642 |
| rs10770131 | Migraine with aura, drug-induced | 11 | 10660701 | C | T | 0.5171 | 0.339544 | 0.078074 | 1.37E-05 | 5.01E-05 | 18.91372 |

**Supplementary Figures**

**Figure S1.** The causal effect of plasma HIF-1α on AS risk. (A) Scatter plot, (B) Funnel plot, (C) Forest plot, and (D) Leave one out plot. AS, Any Stroke.


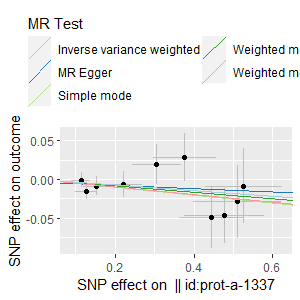

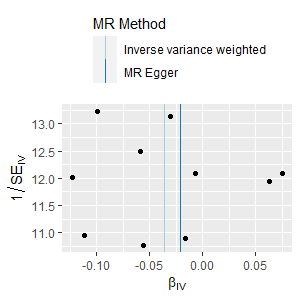
 **A. B.**


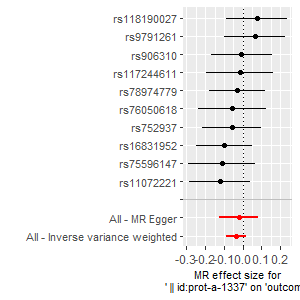
**C. D.**


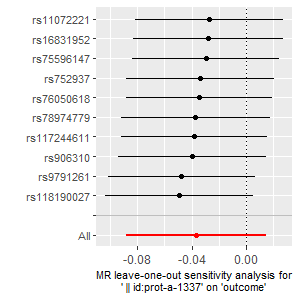


**Figure S2.** The causal effect of plasma HIF-1α on AIS risk. (A) Scatter plot, (B) Funnel plot, (C) Forest plot, and (D) Leave one out plot. AIS, Any Ischemic Stroke.

1. **
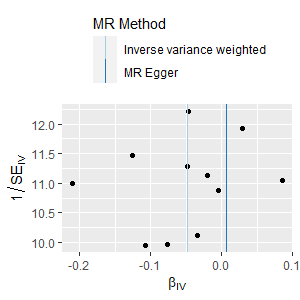

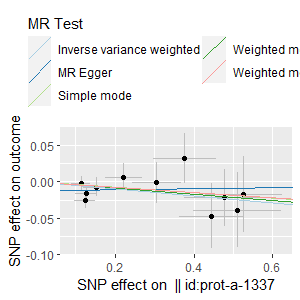
 B.**

**
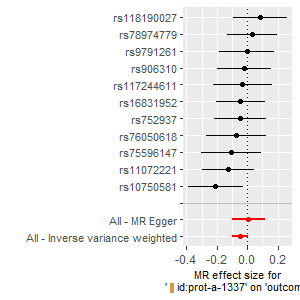

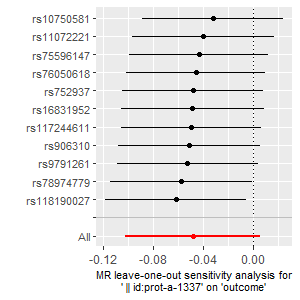
**

**C. D.**

**
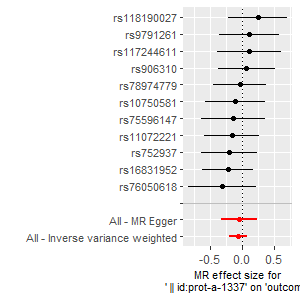

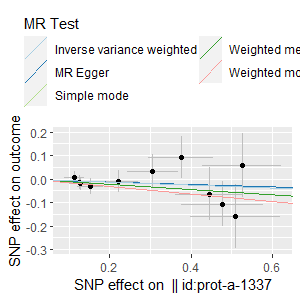

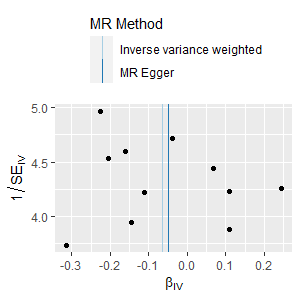

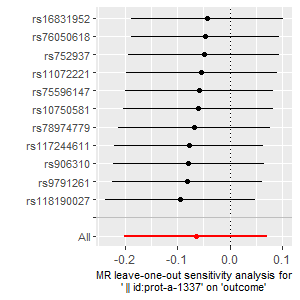
Figure S3.** The causal effect of plasma HIF-1α on ischemic stroke (large artery atherosclerosis) risk. (A) Scatter plot, (B) Funnel plot, (C) Forest plot, and (D) Leave one out plot.

**A.**   **B.**

**C. D.**

**Figure S4.** The causal effect of plasma HIF-1α on ischemic stroke (cardioembolic) risk. (A) Scatter plot, (B) Funnel plot, (C) Forest plot, and (D) Leave one out plot.

**
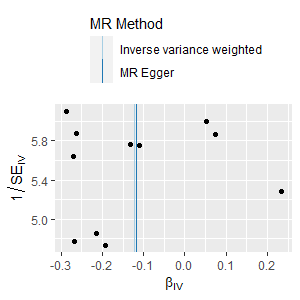

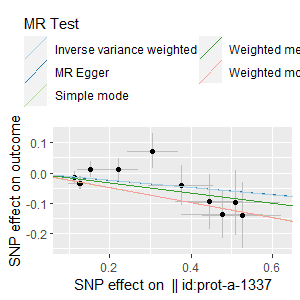
A.**   **B.**

**C. D.**

**
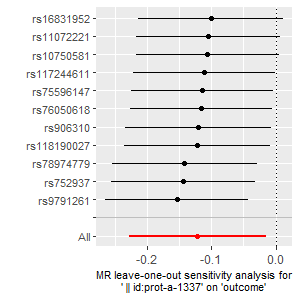

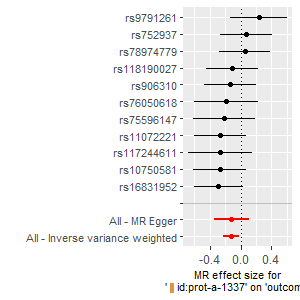
**

**Figure S5.** The causal effect of plasma HIF-1α on ischemic stroke (Small-vessel) risk. (A) Scatter plot, (B) Funnel plot, (C) Forest plot, and (D) Leave one out plot.

1.
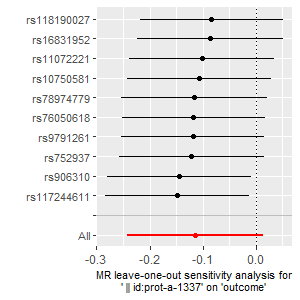

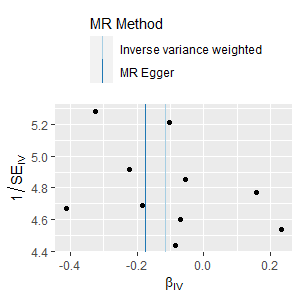

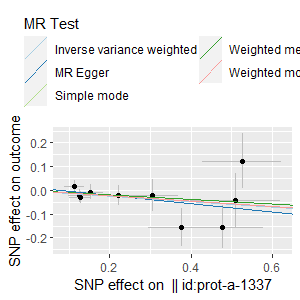
 **B**


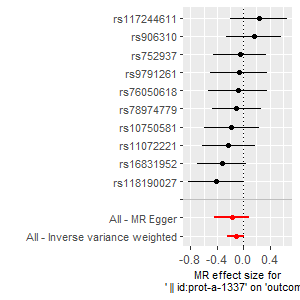
**C. D.**

**Figure S6.** The causal effect of plasma HIF-1α on subarachnoid haemmorrhage risk. (A) Scatter plot, (B) Funnel plot, (C) Forest plot, and (D) Leave one out plot.

1.
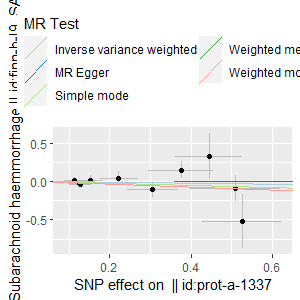

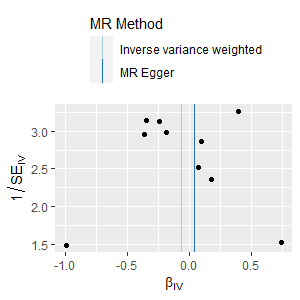
 **B.**


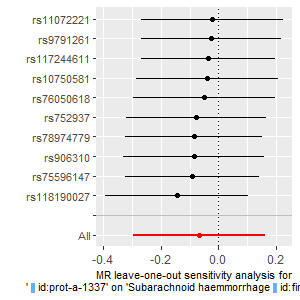

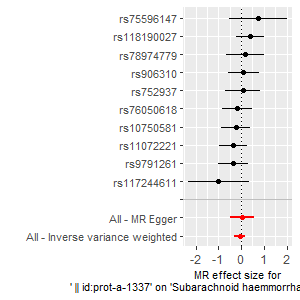
**C. D.**

**Figure S7.** The causal effect of plasma HIF-1α on migraine risk. (A) Scatter plot, (B) Funnel plot, (C) Forest plot, and (D) Leave one out plot.**
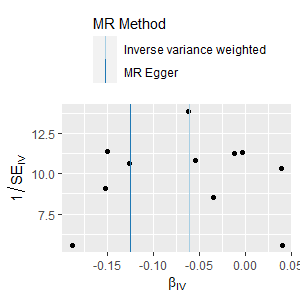

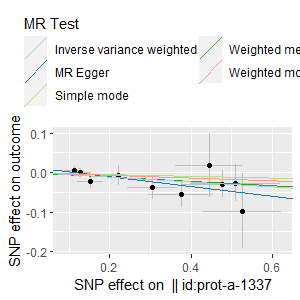
**

**A. B.**

**
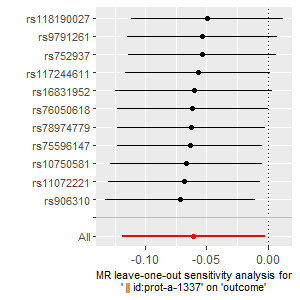
**
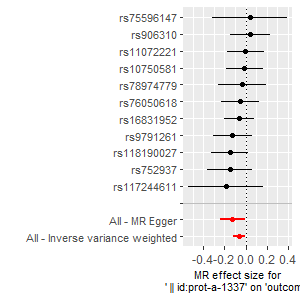
**C. D.**

**Figure S8.** The causal effect of plasma HIF-1α on migraine (without aura) risk. (A) Scatter plot, (B) Funnel plot, (C) Forest plot, and (D) Leave one out plot.


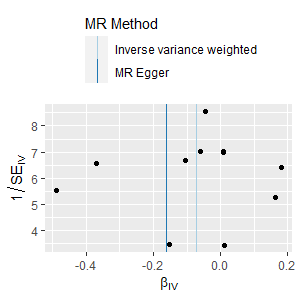

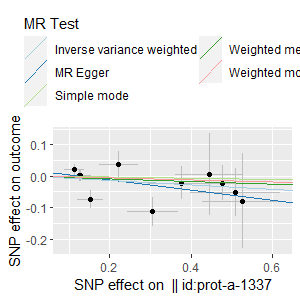
**A. B.**


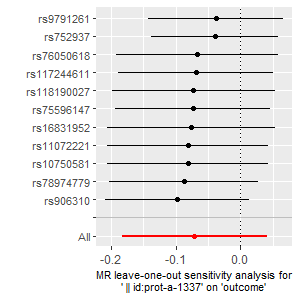

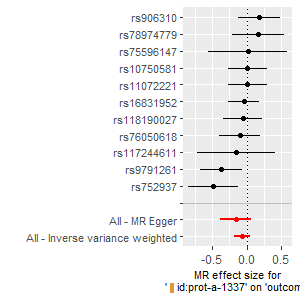
**C. D.**

**Figure S9.** The causal effect of plasma HIF-1α on migraine (without aura, drug-induced) risk. (A) Scatter plot, (B) Funnel plot, (C) Forest plot, and (D) Leave one out plot.

1.
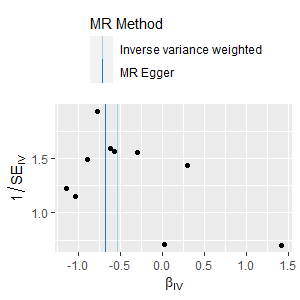

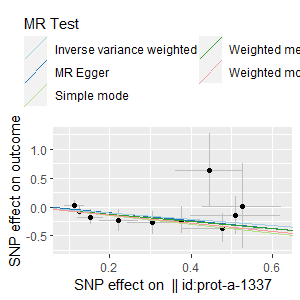
 **B.**

**
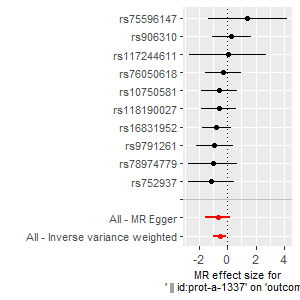

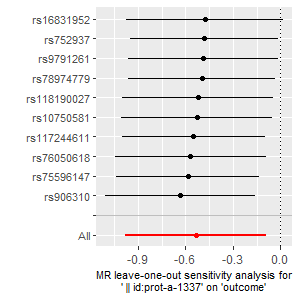
C. D.**


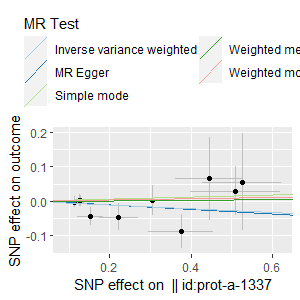
**Figure S10.** The causal effect of plasma HIF-1α on migraine (with aura) risk. (A) Scatter plot, (B) Funnel plot, (C) Forest plot, and (D) Leave one out plot.

1.
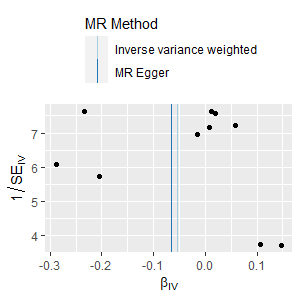
 **B.**


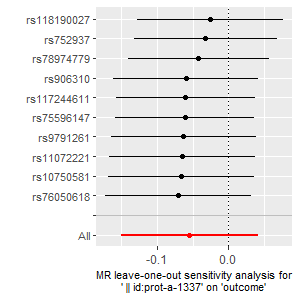

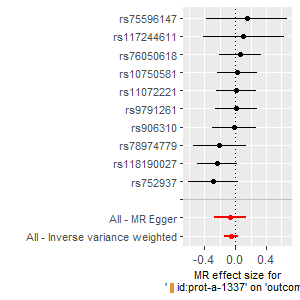
**C. D.**

**Figure S11.** The causal effect of plasma HIF-1α on migraine (with aura, drug-induced) risk. (A) Scatter plot, (B) Funnel plot, (C) Forest plot, and (D) Leave one out plot.

1.
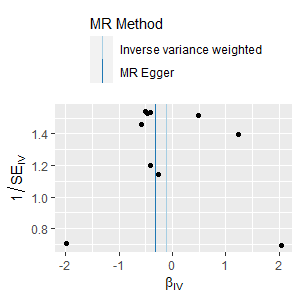

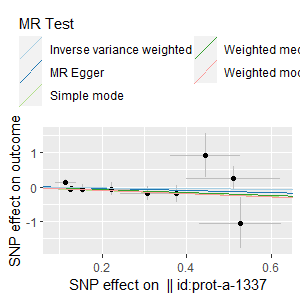
 **B.**

**
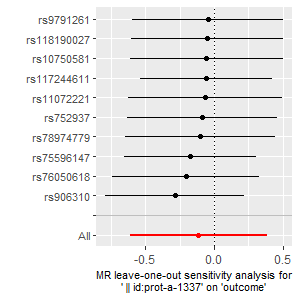

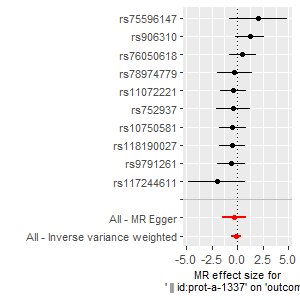
C. D.**

**Figure S12.** The causal effect of plasma HIF-1α on PD risk. (A) Scatter plot, (B) Funnel plot, (C) Forest plot, and (D) Leave one out plot. PD, Parkinson’s disease.

1.
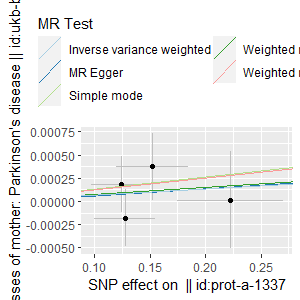

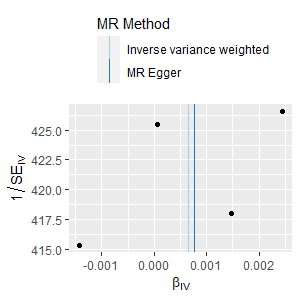
 **B.**


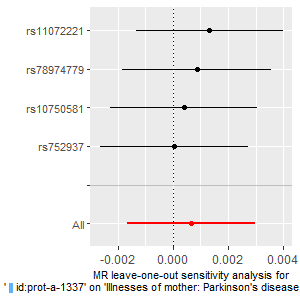

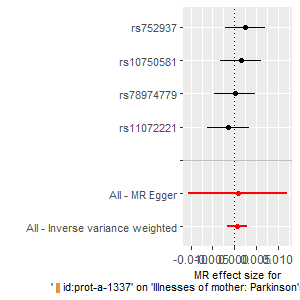
**C. D.**

**Figure S13.** The causal effect of plasma HIF-1α on AD risk. (A) Scatter plot, (B) Funnel plot, (C) Forest plot, and (D) Leave one out plot. AD, Alzheimer’s disease

1.
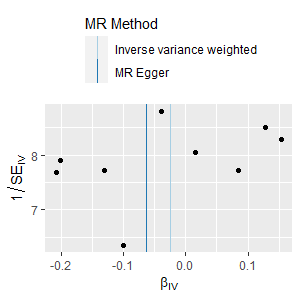

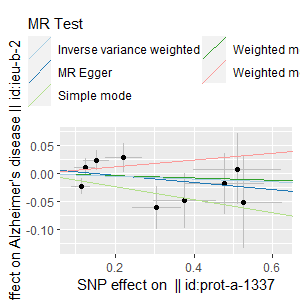
 **B.**

**C. D.**


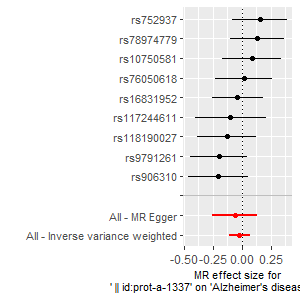

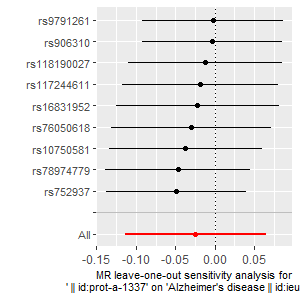


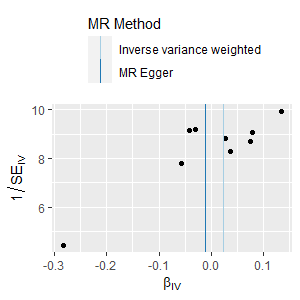

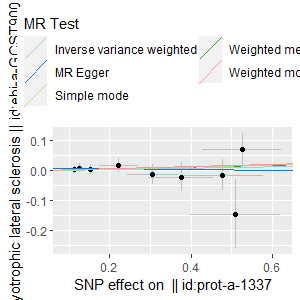
**Figure S14.** The causal effect of plasma HIF-1α on ALS risk. (A) Scatter plot, (B) Funnel plot, (C) Forest plot, and (D) Leave one out plot. ALS, Amyotrophic lateral sclerosis.

1. **B.**


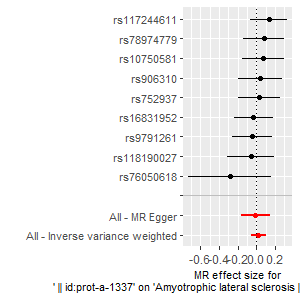


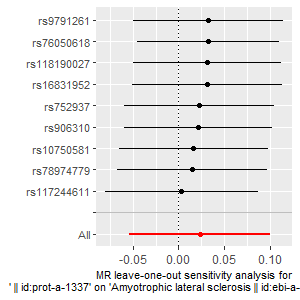
**C. D.**
