# Supplementary material for: Association Between Hypoxia‐Inducible Factor‐1α and Neurological Diseases: A Bidirectional Two‐Sample Mendelian Randomization Analysis
Source: Brain Behav. 2025 Feb 28;15(3):e70398. doi: 10.1002/brb3.70398 (PMC11870835; doi:10.1002/brb3.70398)
Supplement: Supplementary file 2 — Supporting Information [file BRB3-15-e70398-s001.docx]

**Additional File:**

**Supplemental TEXT. Information of investigators of each GWAS dataset in the MR study.**

**1. Genomic atlas of the human plasma proteome**

Benjamin B Sun^1^, Joseph C Maranville^2,3^, James E Peters^1,4^, David Stacey^1^, James R Staley^1^, James Blackshaw^1^, Stephen Burgess^1,5^, Tao Jiang^1^, Ellie Paige^1,6^, Praveen Surendran^1^, Clare Oliver-Williams^1,7^, Mihir A Kamat^1^, Bram P Prins^1^, Sheri K Wilcox^8^, Erik S Zimmerman^8^, An Chi^2^, Narinder Bansal^1,9^, Sarah L Spain^10^, Angela M Wood^1^, Nicholas W Morrell^4,11^, John R Bradley^12^, Nebojsa Janjic^8^, David J Roberts^13,14^, Willem H Ouwehand^4,15,16,17,18^, John A Todd^19^, Nicole Soranzo^4,15,17,18^, Karsten Suhre^20^, Dirk S Paul^1^, Caroline S Fox^2^, Robert M Plenge^2,3^, John Danesh^21,22,23,24^, Heiko Runz^2,25^, Adam S Butterworth^26,27^

^1^MRC/BHF Cardiovascular Epidemiology Unit, Department of Public Health and Primary Care, University of Cambridge, Cambridge, UK.

^2^MRL, Merck & Co., Inc., Kenilworth, NJ, USA.

^3^Celgene Inc., Cambridge, MA, USA.

^4^British Heart Foundation Cambridge Centre of Excellence, Division of Cardiovascular Medicine, Addenbrooke's Hospital, Cambridge, UK.

^5^MRC Biostatistics Unit, University of Cambridge, Cambridge, UK.

^6^National Centre for Epidemiology and Population Health, The Australian National University, Canberra, Australian Capital Territory, Australia.

^7^Homerton College, Cambridge, UK.

^8^SomaLogic Inc, Boulder, CO, USA.

^9^Population Health Sciences, Bristol Medical School, University of Bristol, Bristol, UK.

^10^Wellcome Trust Sanger Institute, Wellcome Trust Genome Campus, Hinxton, Cambridge, UK.

^11^Division of Respiratory Medicine, Department of Medicine, University of Cambridge, Cambridge, UK.

^12^NIHR Cambridge Biomedical Research Centre/BioResource, Cambridge University Hospitals, Cambridge, UK.

^13^National Health Service (NHS) Blood and Transplant and Radcliffe Department of Medicine, NIHR Oxford Biomedical Research Centre, University of Oxford, John Radcliffe Hospital, Oxford, UK.

^14^BRC Haematology Theme and Department of Haematology, Churchill Hospital, Oxford, UK.

^15^Department of Haematology, University of Cambridge, Cambridge Biomedical Campus, Cambridge, UK.

^16^National Health Service (NHS) Blood and Transplant, Cambridge Biomedical Campus, Cambridge, UK.

^17^Department of Human Genetics, Wellcome Trust Sanger Institute, Wellcome Trust Genome Campus, Hinxton, Cambridge, UK.

^18^NIHR Blood and Transplant Research Unit in Donor Health and Genomics, Department of Public Health and Primary Care, University of Cambridge, Cambridge, UK.

^19^JDRF/Wellcome Trust Diabetes and Inflammation Laboratory, Wellcome Trust Centre for Human Genetics, Nuffield Department of Medicine, NIHR Oxford Biomedical Research Centre, University of Oxford, Oxford, UK.

^20^Department of Physiology and Biophysics, Weill Cornell Medicine-Qatar, Doha, Qatar.

^21^MRC/BHF Cardiovascular Epidemiology Unit, Department of Public Health and Primary Care, University of Cambridge, Cambridge, UK. jd292@medschl.cam.ac.uk.

^22^British Heart Foundation Cambridge Centre of Excellence, Division of Cardiovascular Medicine, Addenbrooke's Hospital, Cambridge, UK. jd292@medschl.cam.ac.uk.

^23^Department of Human Genetics, Wellcome Trust Sanger Institute, Wellcome Trust Genome Campus, Hinxton, Cambridge, UK. jd292@medschl.cam.ac.uk.

^24^NIHR Blood and Transplant Research Unit in Donor Health and Genomics, Department of Public Health and Primary Care, University of Cambridge, Cambridge, UK. jd292@medschl.cam.ac.uk.

^25^Biogen Inc., Cambridge, MA, USA.

^26^MRC/BHF Cardiovascular Epidemiology Unit, Department of Public Health and Primary Care, University of Cambridge, Cambridge, UK. asb38@medschl.cam.ac.uk.

^27^NIHR Blood and Transplant Research Unit in Donor Health and Genomics, Department of Public Health and Primary Care, University of Cambridge, Cambridge, UK. asb38@medschl.cam.ac.uk.

**2. Genetic meta-analysis of diagnosed Alzheimer's disease identifies new risk loci and implicates Aβ, tau, immunity and lipid processing**

Brian W Kunkle^1^, Benjamin Grenier-Boley^2,3,4^, Rebecca Sims^5,6^, Joshua C Bis^7^, Vincent Damotte^2,3,4^, Adam C Naj^8^, Anne Boland^9^, Maria Vronskaya^5^, Sven J van der Lee^10^, Alexandre Amlie-Wolf^11^, Céline Bellenguez^2 3 4^, Aura Frizatti^5^, Vincent Chouraki^2,3,4,12,13^, Eden R Martin ^14^, Kristel Sleegers^15,16^, Nandini Badarinarayan^5^, Johanna Jakobsdottir^17^, Kara L Hamilton-Nelson^14^, Sonia Moreno-Grau^18,19^, Robert Olaso^9^, Rachel Raybould^5,6^, Yuning Chen^20^, Amanda B Kuzma^11^, Mikko Hiltunen^21,22^, Taniesha Morgan^5^, Shahzad Ahmad^10^, Badri N Vardarajan^23,24,25^, Jacques Epelbaum^26^, Per Hoffmann^27,28,29^, Merce Boada^18,19^, Gary W Beecham^14^, Jean-Guillaume Garnier^9^, Denise Harold^30^, Annette L Fitzpatrick^31,32^, Otto Valladares^11^, Marie-Laure Moutet^9^, Amy Gerrish^33^, Albert V Smith^34,35^, Liming Qu^11^, Delphine Bacq^9^, Nicola Denning^5,6^, Xueqiu Jian^36^, Yi Zhao^11^, Maria Del Zompo^37^, Nick C Fox^33,38^, Seung-Hoan Choi^18^, Ignacio Mateo^39^, Joseph T Hughes^40^, Hieab H Adams^10^, John Malamon^11^, Florentino Sanchez-Garcia^41^, Yogen Patel^40^, Jennifer A Brody^7^, Beth A Dombroski^11^, Maria Candida Deniz Naranjo^41^, Makrina Daniilidou^42^, Gudny Eiriksdottir^17^, Shubhabrata Mukherjee^43^, David Wallon^44^, James Uphill^45^, Thor Aspelund^17,46^, Laura B Cantwell^11^, Fabienne Garzia^9^, Daniela Galimberti^47,48^, Edith Hofer^49,50^, Mariusz Butkiewicz^51^, Bertrand Fin^9^, Elio Scarpini^47,48^, Chloe Sarnowski^20^, Will S Bush^51^, Stéphane Meslage^9^, Johannes Kornhuber^52^, Charles C White^53^, Yuenjoo Song^51^, Robert C Barber^54^, Sebastiaan Engelborghs ^55,56^, Sabrina Sordon^57^, Dina Voijnovic^10^, Perrie M Adams^58^, Rik Vandenberghe^59^, Manuel Mayhaus^57^, L Adrienne Cupples^12,20^, Marilyn S Albert^60^, Peter P De Deyn^55,56^, Wei Gu^57^, Jayanadra J Himali^12,13,20^, Duane Beekly^61^, Alessio Squassina^37^, Annette M Hartmann^62^, Adelina Orellana^18^, Deborah Blacker^63,64^, Eloy Rodriguez-Rodriguez^39^, Simon Lovestone^65^, Melissa E Garcia^66^, Rachelle S Doody^67^, Carmen Munoz-Fernadez^41^, Rebecca Sussams^68^, Honghuang Lin^69^, Thomas J Fairchild^70^, Yolanda A Benito^41^, Clive Holmes^68^, Hata Karamujić-Čomić^10^, Matthew P Frosch^71^, Hakan Thonberg^72,73^, Wolfgang Maier^74,75^, Gennady Roshchupkin^10^, Bernardino Ghetti^76^, Vilmantas Giedraitis^77^, Amit Kawalia^78^, Shuo Li^20^, Ryan M Huebinger^79^, Lena Kilander^77^, Susanne Moebus^80^, Isabel Hernández^18,19^, M Ilyas Kamboh^81,82,83^, RoseMarie Brundin^77^, James Turto^84^, Qiong Yang^20^, Mindy J Katz^85^, Letizia Concari^86,87^, Jenny Lord^84^, Alexa S Beiser^12,13,20^, C Dirk Keene^88^, Seppo Helisalmi^21,22^, Iwona Kloszewska^89^, Walter A Kukull^32^, Anne Maria Koivisto^21,22^, Aoibhinn Lynch^90,91^, Lluís Tarraga ^18,19^, Eric B Larson^92^, Annakaisa Haapasalo^93^, Brian Lawlor^90,91^, Thomas H Mosley^94^, Richard B Lipton^85^, Vincenzo Solfrizzi^95^, Michael Gill^90,91^, W T Longstreth Jr^32,96^, Thomas J Montine^88^, Vincenza Frisardi^97^, Monica Diez-Fairen^98,99^, Fernando Rivadeneira^10,100,101^, Ronald C Petersen^102^, Vincent Deramecourt^103^, Ignacio Alvarez^98,99^, Francesca Salani^104^, Antonio Ciaramella^104^, Eric Boerwinkle^105,106^, Eric M Reiman^107,108,109,110^, Nathalie Fievet^2,3,4^, Jerome I Rotter^111^, Joan S Reisch^112^, Olivier Hanon^113^, Chiara Cupidi^114^, A G Andre Uitterlinden^10,100,101^, Donald R Royall^115^, Carole Dufouil^116,117^, Raffaele Giovanni Maletta^114^, Itziar de Rojas^18,19^, Mary Sano^118^, Alexis Brice^119,120^, Roberta Cecchetti^121^, Peter St George-Hyslop^122,123^, Karen Ritchie^124,125,126^, Magda Tsolaki^42^, Debby W Tsuang^127,128^, Bruno Dubois^129,130,131,132^, David Craig^133^, Chuang-Kuo Wu^134^, Hilkka Soininen^21,22^, Despoina Avramidou^42^, Roger L Albin^135,136,137^, Laura Fratiglioni^138^, Antonia Germanou^42^, Liana G Apostolova^139,140,141,142^, Lina Keller^138^, Maria Koutroumani^42^, Steven E Arnold^143^, Francesco Panza^97^, Olymbia Gkatzima^42^, Sanjay Asthana^144,145,146^, Didier Hannequin^40^, Patrice Whitehead^14^, Craig S Atwood^140,141,142^, Paolo Caffarra^83,84^, Harald Hampel^147,148,149,150^, Inés Quintela^151^, Ángel Carracedo^151^, Lars Lannfelt^77^, David C Rubinsztein^122,152^, Lisa L Barnes^153,154,155^, Florence Pasquier^103^, Lutz Frölich^156^, Sandra Barral^23,24,25^, Bernadette McGuinness^133^, Thomas G Beach^157^, Janet A Johnston^133^, James T Becker^81,158,159^, Peter Passmore^133^, Eileen H Bigio^160,161^, Jonathan M Schott ^33^, Thomas D Bird^96,127^, Jason D Warren^33^, Bradley F Boeve^102^, Michelle K Lupton^40,162^, James D Bowen^163^, Petra Proitsi^40^, Adam Boxer^164^, John F Powell^40^, James R Burke^165^, John S K Kauwe^166^, Jeffrey M Burns^167^, Michelangelo Mancuso^168^, Joseph D Buxbaum^118,169,170^, Ubaldo Bonuccelli^168^, Nigel J Cairns^171^, Andrew McQuillin^172^, Chuanhai Cao^173^, Gill Livingston^172^, Chris S Carlson^145,146^, Nicholas J Bass^172^, Cynthia M Carlsson^174^, John Hardy^175^, Regina M Carney^176^, Jose Bras^38,177^, Minerva M Carrasquillo^178^, Rita Guerreiro^38,177^, Mariet Allen^178^, Helena C Chui^179^, Elizabeth Fisher^177^, Carlo Masullo^180^, Elizabeth A Crocco^181^, Charles DeCarli ^182^, Gina Bisceglio^178^, Malcolm Dick^183^, Li Ma^178^, Ranjan Duara^184^, Neill R Graff-Radford^178^, Denis A Evans^185^, Angela Hodges^186^, Kelley M Faber^139^, Martin Scherer^187^, Kenneth B Fallon^188^, Matthias Riemenschneider^57^, David W Fardo^189^, Reinhard Heun^75^, Martin R Farlow^141^, Heike Kölsch^75^, Steven Ferris^190^, Markus Leber^191^, Tatiana M Foroud^139^, Isabella Heuser^192^, Douglas R Galasko^193^, Ina Giegling^62^, Marla Gearing^194,195^, Michael Hüll^196^, Daniel H Geschwind^197^, John R Gilbert^14^, John Morris^198,199^, Robert C Green^200^, Kevin Mayo^198,201,202^, John H Growdon^203^, Thomas Feulner^57^, Ronald L Hamilton^204^, Lindy E Harrell^205^, Dmitriy Drichel^206^, Lawrence S Honig^23^, Thomas D Cushion^5,6^, Matthew J Huentelman^107^, Paul Hollingworth^5^, Christine M Hulette^207^, Bradley T Hyman^203^, Rachel Marshall^5^, Gail P Jarvik^208,209^, Alun Meggy^5^, Erin Abner^210^, Georgina E Menzies^5,6^, Lee-Way Jin^211^, Ganna Leonenko^5^, Luis M Real^211^, Gyungah R Jun^212^, Clinton T Baldwin^212^, Detelina Grozeva^5^, Anna Karydas^163^, Giancarlo Russo^213^, Jeffrey A Kaye^214,215^, Ronald Kim^216^, Frank Jessen^74,75,191^, Neil W Kowall^13,217^, Bruno Vellas^218^, Joel H Kramer^219^, Emma Vardy^220^, Frank M LaFerla^221^, Karl-Heinz Jöckel^80^, James J Lah^222^, Martin Dichgans^223,224^, James B Leverenz^225^, David Mann^226^, Allan I Levey^222^, Stuart Pickering-Brown^226^, Andrew P Lieberman^227^, Norman Klopp^228^, Kathryn L Lunetta^20^, H-Erich Wichmann^229,230,231^, Constantine G Lyketsos^232^, Kevin Morgan^233^, Daniel C Marson^205^, Kristelle Brown^84^, Frank Martiniuk^234^, Christopher Medway^84^, Deborah C Mash^235^, Markus M Nöthen^27,28^, Eliezer Masliah^193,236^, Nigel M Hooper^226^, Wayne C McCormick^43^, Antonio Daniele^237^, Susan M McCurry^238^, Anthony Bayer^239^, Andrew N McDavid^173^, John Gallacher^65^, Ann C McKee^13,217^, Hendrik van den Bussche^187^, Marsel Mesulam^161, 240^, Carol Brayne^241^, Bruce L Miller^242^, Steffi Riedel-Heller^243^, Carol A Miller^244^, Joshua W Miller^245^, Ammar Al-Chalabi^246^, John C Morris^171,201^, Christopher E Shaw^246,247^, Amanda J Myers^181^, Jens Wiltfang^248,249,250^, Sid O'Bryant^54^, John M Olichney^182^, Victoria Alvarez^251^, Joseph E Parisi^252^, Andrew B Singleton^253^, Henry L Paulson^135,137^, John Collinge^45^, William R Perry^14^, Simon Mead^45^, Elaine Peskind^128^, David H Cribbs^254^, Martin Rossor^33^, Aimee Pierce^254^, Natalie S Ryan^45^, Wayne W Poon^183^, Benedetta Nacmias^255,256^, Huntington Potter^257^, Sandro Sorbi^255,258^, Joseph F Quinn^187,188^, Eleonora Sacchinelli^104^, Ashok Raj^173^, Gianfranco Spalletta^259,260^, Murray Raskind^128^, Carlo Caltagirone^259^, Paola Bossù^104^, Maria Donata Orfei^259^, Barry Reisberg^190,261^, Robert Clarke^262^, Christiane Reitz^23,24,263^, A David Smith^264^, John M Ringman^265^, Donald Warden^264^, Erik D Roberson^205^, Gordon Wilcock ^264^, Ekaterina Rogaeva^123^, Amalia Cecilia Bruni^114^, Howard J Rosen^164^, Maura Gallo^114^, Roger N Rosenberg^266^, Yoav Ben-Shlomo^267^, Mark A Sager^145^, Patrizia Mecocci^121^, Andrew J Saykin^139,141^, Pau Pastor^98,99^, Michael L Cuccaro^14^, Jeffery M Vance^14^, Julie A Schneider^153,155,268^, Lori S Schneider^179,269^, Susan Slifer^14^, William W Seeley^164^, Amanda G Smith^173^, Joshua A Sonnen^88^, Salvatore Spina^76^, Robert A Stern^13^, Russell H Swerdlow^167^, Mitchell Tang^11^, Rudolph E Tanzi^203^, John Q Trojanowski^270^, Juan C Troncoso^271^, Vivianna M Van Deerlin^270^, Linda J Van Eldik^272^, Harry V Vinters^273,274^, Jean Paul Vonsattel^275^, Sandra Weintraub^161,276^, Kathleen A Welsh-Bohmer^165,277^, Kirk C Wilhelmsen^278^, Jennifer Williamson^23^, Thomas S Wingo^222,279^, Randall L Woltjer^280^, Clinton B Wright^281^, Chang-En Yu ^43^, Lei Yu^153,155^, Yasaman Saba^282^, Alberto Pilotto^283,284^, Maria J Bullido^19,285,286^, Oliver Peters ^192,287^, Paul K Crane^43^, David Bennett^153,155^, Paola Bosco^288^, Eliecer Coto^251^, Virginia Boccardi^121^, Phil L De Jager^289^, Alberto Lleo^19,290^, Nick Warner^291^, Oscar L Lopez^81,83,158^, Martin Ingelsson^77^, Panagiotis Deloukas^292^, Carlos Cruchaga^198,199^, Caroline Graff^72,73^, Rhian Gwilliam^292^, Myriam Fornage^36^, Alison M Goate^169,293^, Pascual Sanchez-Juan^39^, Patrick G Kehoe^294^, Najaf Amin^10^, Nilifur Ertekin-Taner^178,295^, Claudine Berr^124,125^, Stéphanie Debette^119,120^, Seth Love^294^, Lenore J Launer^66^, Steven G Younkin^178,295^, Jean-Francois Dartigues^296^, Chris Corcoran^297^, M Arfan Ikram^10,298,299^, Dennis W Dickson^178^, Gael Nicolas^44^, Dominique Campion^44,300^, JoAnn Tschanz^297^, Helena Schmidt^282,301^, Hakon Hakonarson^302,303^, Jordi Clarimon^19,290^, Ron Munger^297^, Reinhold Schmidt^49^, Lindsay A Farrer^13,20,212,304,305^, Christine Van Broeckhoven^15,16^, Michael C O'Donovan^5^, Anita L DeStefano^13,20^, Lesley Jones^5,6^, Jonathan L Haines^51^, Jean-Francois Deleuze^9^, Michael J Owen^5^, Vilmundur Gudnason^17,35^, Richard Mayeux^23,24,25^, Valentina Escott-Price^5,6^, Bruce M Psaty^7,32,306,307^, Alfredo Ramirez^78,191^, Li-San Wang^11^, Agustin Ruiz^18,19^, Cornelia M van Duijn^10^, Peter A Holmans^5^, Sudha Seshadri^12,13,308^, Julie Williams^5,6^, Phillippe Amouyel^2,3,4,309^, Gerard D Schellenberg^11^, Jean-Charles Lambert^310,311,312^, Margaret A Pericak-Vance^313^, Alzheimer Disease Genetics Consortium (ADGC),; European Alzheimer’s Disease Initiative (EADI),; Cohorts for Heart and Aging Research in Genomic Epidemiology Consortium (CHARGE),; Genetic and Environmental Risk in AD/Defining Genetic, Polygenic and Environmental Risk for Alzheimer’s Disease Consortium (GERAD/PERADES),

^1^John P. Hussman Institute for Human Genomics, University of Miami Miller School of Medicine, Miami, FL, USA. bkunkle@miami.edu.

^2^Inserm, U1167, RID-AGE-Risk Factors and Molecular Determinants of Aging-Related Diseases, Lille, France.

^3^Institut Pasteur de Lille, Lille, France.

^4^Univ. Lille, U1167-Excellence Laboratory LabEx DISTALZ, Lille, France.

^5^Division of Psychological Medicine and Clinical Neurosciences, MRC Centre for Neuropsychiatric Genetics and Genomics, Cardiff University, Cardiff, UK.

^6^UK Dementia Research Institute at Cardiff, Cardiff University, Cardiff, UK.

^7^Cardiovascular Health Research Unit, Department of Medicine, University of Washington, Seattle, WA, USA.

^8^Department of Biostatistics and Epidemiology/Center for Clinical Epidemiology and Biostatistics, University of Pennsylvania Perelman School of Medicine, Philadelphia, PA, USA.

^9^Centre National de Recherche en Génomique Humaine, Institut de Biologie François Jacob, CEA, Université Paris-Saclay, and LabEx GENMED, Evry, France.

^10^Department of Epidemiology, Erasmus Medical Center, Rotterdam, the Netherlands.

^11^Penn Neurodegeneration Genomics Center, Department of Pathology and Laboratory Medicine, University of Pennsylvania Perelman School of Medicine, Philadelphia, PA, USA.

^12^Framingham Heart Study, Framingham, MA, USA.

^13^Department of Neurology, Boston University School of Medicine, Boston, MA, USA.

^14^John P. Hussman Institute for Human Genomics, University of Miami Miller School of Medicine, Miami, FL, USA.

^15^Neurodegenerative Brain Diseases Group, Center for Molecular Neurology, VIB, Antwerp, Belgium.

^16^Laboratory for Neurogenetics, Institute Born-Bunge, University of Antwerp, Antwerp, Belgium.

^17^Icelandic Heart Association, Kopavogur, Iceland.

^18^Research Center and Memory Clinic of Fundació ACE, Institut Català de Neurociències Aplicades-Universitat Internacional de Catalunya, Barcelona, Spain.

^19^Centro de Investigación Biomédica en Red de Enfermedades Neurodegenerativas, Instituto de Salud Carlos III, Madrid, Spain.

^20^Department of Biostatistics, Boston University School of Public Health, Boston, MA, USA.

^21^Institute of Biomedicine, University of Eastern Finland, Kuopio, Finland.

^22^Department of Neurology, Kuopio University Hospital, Kuopio, Finland.

^23^Taub Institute on Alzheimer's Disease and the Aging Brain, Department of Neurology, Columbia University, New York, NY, USA.

^24^Gertrude H. Sergievsky Center, Columbia University, New York, NY, USA.

^25^Department of Neurology, Columbia University, New York, NY, USA.

^26^UMR 894, Center for Psychiatry and Neuroscience, Inserm, Université Paris Descartes, Paris, France.

^27^Institute of Human Genetics, University of Bonn, Bonn, Germany.

^28^Department of Genomics, Life & Brain Center, University of Bonn, Bonn, Germany.

^29^Division of Medical Genetics, University Hospital and Department of Biomedicine, University of Basel, Basel, Switzerland.

^30^School of Biotechnology, Dublin City University, Dublin, Ireland.

^31^Department of Family Medicine, University of Washington, Seattle, WA, USA.

^32^Department of Epidemiology, University of Washington, Seattle, WA, USA.

^33^Dementia Research Centre, Department of Neurodegenerative Disease, UCL Institute of Neurology, London, UK.

^34^Department of Biostatistics, University of Michigan, Ann Arbor, MI, USA.

^35^Faculty of Medicine, University of Iceland, Reykjavik, Iceland.

^36^Brown Foundation Institute of Molecular Medicine, University of Texas Health Sciences Center at Houston, Houston, TX, USA.

^37^Section of Neuroscience and Clinical Pharmacology, Department of Biomedical Sciences, University of Cagliari, Cagliari, Italy.

^38^UK Dementia Research Institute at UCL, Department of Neurodegenerative Disease, UCL Institute of Neurology, London, UK.

^39^Neurology Service and CIBERNED, 'Marqués de Valdecilla' University Hospital (University of Cantabria and IDIVAL), Santander, Spain.

^40^Department of Basic and Clinical Neuroscience, Institute of Psychiatry, Psychology and Neuroscience, King's College London, London, UK.

^41^Department of Immunology, Hospital Universitario Doctor Negrín, Las Palmas de Gran Canaria, Spain.

^42^Department of Neurology, Medical School, Aristotle University of Thessaloniki, Thessaloniki, Greece.

^43^Department of Medicine, University of Washington, Seattle, WA, USA.

^44^Normandie University, UNIROUEN, Inserm U1245, and Rouen University Hospital, Department of Neurology, Department of Genetics and CNR-MAJ, Normandy Center for Genomic and Personalized Medicine, Rouen, France.

^45^Department of Neurodegenerative Disease, MRC Prion Unit at UCL, Institute of Prion Diseases, London, UK.

^46^Centre for Public Health, University of Iceland, Reykjavik, Iceland.

^47^Fondazione IRCCS Ca' Granda, Ospedale Maggiore Policlinico, Neurodegenerative Diseases Unit, Milan, Italy.

^48^University of Milan, Centro Dino Ferrari, Milan, Italy.

^49^Clinical Division of Neurogeriatrics, Department of Neurology, Medical University Graz, Graz, Austria.

^50^Institute for Medical Informatics, Statistics and Documentation, Medical University of Graz, Graz, Austria.

^51^Institute for Computational Biology, Department of Population & Quantitative Health Sciences, Case Western Reserve University, Cleveland, OH, USA.

^52^Department of Psychiatry and Psychotherapy, University of Erlangen-Nuremberg, Erlangen, Germany.

^53^Program in Medical and Population Genetics, Broad Institute, Cambridge, MA, USA.

^54^Department of Pharmacology and Neuroscience, University of North Texas Health Science Center, Fort Worth, TX, USA.

^55^Laboratory for Neurochemistry and Behavior, Institute Born-Bunge, University of Antwerp, Antwerp, Belgium.

^56^Department of Neurology and Memory Clinic, Hospital Network Antwerp, Antwerp, Belgium.

^57^Department of Psychiatry and Psychotherapy, University Hospital, Saarland, Germany.

^58^Department of Psychiatry, University of Texas Southwestern Medical Center, Dallas, TX, USA.

^59^Laboratory for Cognitive Neurology, Department of Neurology, University Hospital and University of Leuven, Leuven, Belgium.

^60^Department of Neurology, Johns Hopkins University, Baltimore, MD, USA.

^61^National Alzheimer's Coordinating Center, University of Washington, Seattle, WA, USA.

^62^Department of Psychiatry, Martin Luther University Halle-Wittenberg, Halle, Germany.

^63^Department of Epidemiology, Harvard T.H. Chan School of Public Health, Harvard University, Boston, MA, USA.

^64^Department of Psychiatry, Massachusetts General Hospital/Harvard Medical School, Boston, MA, USA.

^65^Department of Psychiatry, University of Oxford, Oxford, UK.

^66^Laboratory of Epidemiology and Population Sciences, National Institute on Aging, Bethesda, MD, USA.

^67^Alzheimer's Disease and Memory Disorders Center, Baylor College of Medicine, Houston, TX, USA.

^68^Division of Clinical Neurosciences, School of Medicine, University of Southampton, Southampton, UK.

^69^Section of Computational Biomedicine, Department of Medicine, Boston University School of Medicine, Boston, MA, USA.

^70^Office of Strategy and Measurement, University of North Texas Health Science Center, Fort Worth, TX, USA.

^71^C.S. Kubik Laboratory for Neuropathology, Massachusetts General Hospital, Charlestown, MA, USA.

^72^Theme Aging, Unit for Hereditary Dementias, Karolinska University Hospital-Solna, Stockholm, Sweden.

^73^Karolinska Institutet, Department of Neurobiology, Care Sciences and Society, Alzheimer Research Center, Division of Neurogeriatrics, Solna, Sweden.

^74^German Center for Neurodegenerative Diseases, Bonn, Germany.

^75^Department of Psychiatry and Psychotherapy, University of Bonn, Bonn, Germany.

^76^Department of Pathology and Laboratory Medicine, Indiana University, Indianapolis, IN, USA.

^77^Department of Public Health and Caring Sciences/Geriatrics, Uppsala University, Uppsala, Sweden.

^78^Department for Neurodegenerative Diseases and Geriatric Psychiatry, University Hospital Bonn, Bonn, Germany.

^79^Department of Surgery, University of Texas Southwestern Medical Center, Dallas, TX, USA.

80Institute for Medical Informatics, Biometry and Epidemiology, University Hospital of Essen, University Duisburg-Essen, Essen, Germany.

^81^Department of Psychiatry, University of Pittsburgh, Pittsburgh, PA, USA.

^82^Department of Human Genetics, University of Pittsburgh, Pittsburgh, PA, USA.

^83^Alzheimer's Disease Research Center, University of Pittsburgh, Pittsburgh, PA, USA.

^84^Institute of Genetics, Queen's Medical Centre, University of Nottingham, Nottingham, UK.

^85^Department of Neurology, Albert Einstein College of Medicine, Bronx, NY, USA.

^86^Section of Neuroscience, DIMEC-University of Parma, Parma, Italy.

^87^FERB-Alzheimer Center, Gazzaniga (Bergamo), Italy.

^88^Department of Pathology, University of Washington, Seattle, WA, USA.

^89^Elderly and Psychiatric Disorders Department, Medical University of Lodz, Lodz, Poland.

^90^Mercer's Institute for Research on Aging, St. James's Hospital and Trinity College, Dublin, Ireland.

^91^St. James's Hospital and Trinity College, Dublin, Ireland.

^92^Kaiser Permanente Washington Health Research Institute, Seattle, WA, USA.

^93^A.I. Virtanen Institute for Molecular Sciences, University of Eastern Finland, Kuopio, Finland.

^94^Departments of Medicine, Geriatrics, Gerontology and Neurology, University of Mississippi Medical Center, Jackson, MS, USA.

^95^Interdisciplinary Department of Medicine, Geriatric Medicine and Memory Unity, University of Bari, Bari, Italy.

^96^Department of Neurology, University of Washington, Seattle, WA, USA.

^97^Department of Geriatrics, Center for Aging Brain, University of Bari, Bari, Italy.

^98^Fundació per la Recerca Biomèdica i Social Mútua Terrassa, Terrassa, Barcelona, Spain.

^99^Memory Disorders Unit, Department of Neurology, Hospital Universitari Mutua de Terrassa, Terrassa, Barcelona, Spain.

^100^Department of Internal Medicine, Erasmus University Medical Center, Rotterdamt, the Netherlands.

^101^Netherlands Consortium on Health Aging and National Genomics Initiative, Leiden, the Netherlands.

^102^Department of Neurology, Mayo Clinic, Rochester, MN, USA.

^103^CHU Lille, Memory Center of Lille (Centre Mémoire de Ressources et de Recherche), Lille, France.

^104^Department of Clinical and Behavioral Neurology, Experimental Neuropsychobiology Laboratory, IRCCS Santa Lucia Foundation, Rome, Italy.

^105^School of Public Health, Human Genetics Center, University of Texas Health Science Center at Houston, Houston, TX, USA.

^106^Human Genome Sequencing Center, Baylor College of Medicine, Houston, TX, USA.

^107^Neurogenomics Division, Translational Genomics Research Institute, Phoenix, AZ, USA.

^108^Arizona Alzheimer's Consortium, Phoenix, AZ, USA.

^109^Banner Alzheimer's Institute, Phoenix, AZ, USA.

^110^Department of Psychiatry, University of Arizona, Phoenix, AZ, USA.

^111^Institute for Translational Genomics and Population Sciences, Departments of Pediatrics and Medicine, Los Angeles BioMedical Research Institute at Harbor-UCLA Medical Center, Torrance, CA, USA.

^112^Department of Clinical Sciences, University of Texas Southwestern Medical Center, Dallas, TX, USA.

^113^University Paris Descartes, EA 4468, AP-HP, Geriatrics Department, Hôpital Broca, Paris, France.

^114^Regional Neurogenetic Centre (CRN), ASP Catanzaro, Lamezia Terme, Italy.

^115^Departments of Psychiatry, Medicine, Family & Community Medicine, South Texas Veterans Health Administration Geriatric Research Education & Clinical Center (GRECC), UT Health Science Center at San Antonio, San Antonio, TX, USA.

^116^University of Bordeaux, Inserm 1219, Bordeaux, France.

^117^Department of Neurology, Bordeaux University Hospital / CHU de Bordeaux, Bordeaux, France.

^118^Department of Psychiatry, Icahn School of Medicine at Mount Sinai, New York, NY, USA.

^119^Inserm U1127, CNRS UMR 7225, Sorbonne Universités, UPMC Université Paris 06, UMRS 1127, Institut du Cerveau et de la Moelle Épinière, Paris, France.

^120^AP-HP, Department of Genetics, Pitié-Salpêtrière Hospital, Paris, France.

^121^Section of Gerontology and Geriatrics, Department of Medicine, University of Perugia, Perugia, Italy.

^122^Cambridge Institute for Medical Research, University of Cambridge, Cambridge, UK.

^123^Tanz Centre for Research in Neurodegenerative Disease, University of Toronto, Toronto, Ontario, Canada.

^124^Inserm U1061 Neuropsychiatry, La Colombière Hospital, Montpellier, France.

^125^Montpellier University, Montpellier, France.

^126^Department of Clinical Brain Sciences, University of Edinburgh, Edinburgh, UK.

^127^VA Puget Sound Health Care System/>GRECC, Seattle, WA, USA.

^128^Department of Psychiatry and Behavioral Sciences, University of Washington School of Medicine, Seattle, WA, USA.

^129^Institut de la Mémoire et de la Maladie d'Alzheimer and Institut du Cerveau et de la Moelle Épinière, Département de Neurologie, Hôpital de la Pitié-Salpêtrière, Paris, France.

^130^Institut des Neurosciences Translationnelles de Paris, Institut du Cerveau et de la Moelle Épinière, Paris, France.

^131^Inserm, CNRS, UMR-S975, Institut du Cerveau et de la Moelle Epinière, Paris, France.

^132^Sorbonne Universités, Université Pierre et Marie Curie, Hôpital de la Pitié-Salpêtrière, AP-HP, Paris, France.

^133^Ageing Group, Centre for Public Health, School of Medicine, Dentistry and Biomedical Sciences, Queen's University Belfast, Belfast, UK.

^134^Departments of Neurology, Pharmacology & Neuroscience, Texas Tech University Health Science Center, Lubbock, TX, USA.

^135^Department of Neurology, University of Michigan, Ann Arbor, MI, USA.

^136^Geriatric Research, Education and Clinical Center (GRECC), VA Ann Arbor Healthcare System (VAAAHS), Ann Arbor, MI, USA.

^137^Michigan Alzheimer Disease Center, Ann Arbor, MI, USA.

^138^Aging Research Center, Department of Neurobiology, Care Sciences and Society, Karolinska Institutet and Stockholm University, Stockholm, Sweden.

^139^Indiana Alzheimer's Disease Center, Indiana University School of Medicine, Indianapolis, IN, USA.

^140^Department of Medical and Molecular Genetics, Indiana University, Indianapolis, IN, USA.

^141^Department of Neurology, Indiana University, Indianapolis, IN, USA.

^142^Department of Radiology and Imaging Sciences, Indiana University, Indianapolis, IN, USA.

^143^Department of Psychiatry, University of Pennsylvania Perelman School of Medicine, Philadelphia, PA, USA.

^144^Geriatric Research, Education and Clinical Center (GRECC), University of Wisconsin, Madison, WI, USA.

^145^Department of Medicine, University of Wisconsin, Madison, WI, USA.

^146^Wisconsin Alzheimer's Disease Research Center, Madison, WI, USA.

^147^AXA Research Fund & Sorbonne University Chair, Paris, France.

^148^Sorbonne University, GRC n° 21, Alzheimer Precision Medicine (APM), AP-HP, Pitié-Salpêtrière Hospital, Paris, France.

^149^Brain & Spine Institute, Inserm U 1127, CNRS UMR 7225, Paris, France.

^150^Institute of Memory and Alzheimer's Disease, Department of Neurology, Pitié-Salpêtrière Hospital, AP-HP, Paris, France.

^151^Grupo de Medicina Xenomica, Universidade de Santiago de Compostela, Centro Nacional de Genotipado, Centro de Investigación Biomédica en Red de Enfermedades Raras, Santiago de Compostela, Spain.

^152^UK Dementia Research Institute, University of Cambridge, Cambridge, UK.

^153^Department of Neurological Sciences, Rush University Medical Center, Chicago, IL, USA.

^154^Department of Behavioral Sciences, Rush University Medical Center, Chicago, IL, USA.

^155^Rush Alzheimer's Disease Center, Rush University Medical Center, Chicago, IL, USA.

^156^Central Institute of Mental Health, Medical Faculty Mannheim, University of Heidelberg, Heidelberg, Germany.

^157^Civin Laboratory for Neuropathology, Banner Sun Health Research Institute, Phoenix, AZ, USA.

^158^Department of Neurology, University of Pittsburgh, Pittsburgh, PA, USA.

^159^Department of Psychology, University of Pittsburgh School of Medicine, Pittsburgh, PA, USA.

^160^Department of Pathology, Northwestern University Feinberg School of Medicine, Chicago, IL, USA.

^161^Mesulam Center for Cognitive Neurology and Alzheimer's Disease, Northwestern University Feinberg School of Medicine, Chicago, IL, USA.

^162^Genetic Epidemiology, QIMR Berghofer Medical Research Institute, Herston, Queensland, Australia.

^163^Swedish Medical Center, Seattle, WA, USA.

^164^Department of Neurology, University of California, San Francisco, San Francisco, CA, USA.

^165^Department of Neurology, Duke University, Durham, NC, USA.

^166^Departments of Biology, Brigham Young University, Provo, UT, USA.

^167^University of Kansas Alzheimer's Disease Center, University of Kansas Medical Center, Kansas City, KS, USA.

^168^Department of Experimental and Clinical Medicine, Neurological Institute, University of Pisa, Pisa, Italy.

^169^Department of Genetics and Genomic Sciences, Icahn School of Medicine at Mount Sinai, New York, NY, USA.

^170^Department of Neuroscience, Icahn School of Medicine at Mount Sinai, New York, NY, USA.

^171^Department of Pathology and Immunology, Washington University, St. Louis, MO, USA.

^172^Division of Psychiatry, University College London, London, UK.

^173^USF Health Byrd Alzheimer's Institute, University of South Florida, Tampa, FL, USA.

^174^Fred Hutchinson Cancer Research Center, Seattle, WA, USA.

^175^Department of Molecular Neuroscience, UCL, Institute of Neurology, London, UK.

^176^Mental Health & Behavioral Science Service, Bruce W. Carter VA Medical Center, Miami, FL, USA.

^177^Department of Neurodegenerative Disease, UCL Institute of Neurology, London, UK.

^178^Department of Neuroscience, Mayo Clinic, Jacksonville, FL, USA.

^179^Department of Neurology, University of Southern California, Los Angeles, CA, USA.

^180^Department of Neurology, Catholic University of Rome, Rome, Italy.

^181^Department of Psychiatry and Behavioral Sciences, Miller School of Medicine, University of Miami, Miami, FL, USA.

^182^Department of Neurology, University of California, Davis, Sacramento, CA, USA.

^183^Institute for Memory Impairments and Neurological Disorders, University of California, Irvine, Irvine, CA, USA.

^184^Wien Center for Alzheimer's Disease and Memory Disorders, Mount Sinai Medical Center, Miami Beach, FL, USA.

^185^Rush Institute for Healthy Aging, Department of Internal Medicine, Rush University Medical Center, Chicago, IL, USA.

^186^Department of Old Age Psychiatry, Institute of Psychiatry, Psychology and Neuroscience, King's College London, London, UK.

^187^Department of Primary Medical Care, University Medical Centre Hamburg-Eppendorf, Hamburg, Germany.

^188^Department of Pathology, University of Alabama at Birmingham, Birmingham, AL, USA.

^189^Sanders-Brown Center on Aging, Department of Biostatistics, University of Kentucky, Lexington, KY, USA.

^190^Department of Psychiatry, New York University, New York, NY, USA.

^191^Department of Psychiatry and Psychotherapy, University of Cologne, Cologne, Germany.

^192^Department of Psychiatry and Psychotherapy, Charité University Medicine, Berlin, Germany.

^193^Department of Neurosciences, University of California, San Diego, La Jolla, CA, USA.

^194^Department of Pathology and Laboratory Medicine, Emory University, Atlanta, GA, USA.

^195^Emory Alzheimer's Disease Center, Emory University, Atlanta, GA, USA.

1^96^Department of Psychiatry, University of Freiburg, Freiburg, Germany.

^197^Neurogenetics Program, University of California, Los Angeles, Los Angeles, CA, USA.

^198^Department of Psychiatry, Washington University School of Medicine, St. Louis, MO, USA.

^199^Hope Center Program on Protein Aggregation and Neurodegeneration, Washington University School of Medicine, St. Louis, MO, USA.

^200^Division of Genetics, Department of Medicine and Partners Center for Personalized Genetic Medicine, Brigham and Women's Hospital and Harvard Medical School, Boston, MA, USA.

^201^Department of Neurology, Washington University, St. Louis, MO, USA.

^202^Department of Genetics, Washington University, St. Louis, MO, USA.

^203^Department of Neurology, Massachusetts General Hospital/Harvard Medical School, Boston, MA, USA.

^204^Department of Pathology (Neuropathology), University of Pittsburgh, Pittsburgh, PA, USA.

^205^Department of Neurology, University of Alabama at Birmingham, Birmingham, AL, USA.

^206^Cologne Center for Genomics, University of Cologne, Cologne, Germany.

^207^Department of Pathology, Duke University, Durham, NC, USA.

^208^Department of Genome Sciences, University of Washington, Seattle, WA, USA.

^209^Department of Medicine (Medical Genetics), University of Washington, Seattle, WA, USA.

^210^Sanders-Brown Center on Aging, College of Public Health, Department of Epidemiology, University of Kentucky, Lexington, KY, USA.

^211^Unidad Clínica de Enfermedades Infecciosas y Microbiología, Hospital Universitario de Valme, Sevilla, Spain.

^212^Department of Medicine (Biomedical Genetics), Boston University School of Medicine, Boston, MA, USA.

^213^Functional Genomics Center Zurich, ETH/University of Zurich, Zurich, Switzerland.

^214^Department of Neurology, Oregon Health &Science University, Portland, OR, USA.

^215^Department of Neurology, Portland Veterans Affairs Medical Center, Portland, OR, USA.

^216^Department of Pathology and Laboratory Medicine, University of California, Irvine, Irvine, CA, USA.

^217^Department of Pathology, Boston University School of Medicine, Boston University, Boston, MA, USA.

^218^Inserm U558, University of Toulouse, Toulouse, France.

^219^Department of Neuropsychology, University of California San Francisco, San Francisco, CA, USA.

^220^Institute for Ageing and Health, Newcastle University, Newcastle upon Tyne, UK.

^221^Department of Neurobiology and Behavior, University of California, Irvine, Irvine, CA, USA.

^222^Department of Neurology, Emory University, Atlanta, GA, USA.

^223^Institute for Stroke and Dementia Research, Klinikum der Universität München, Munich, Germany.

^224^German Center for Neurodegenerative Diseases, Munich, Germany.

^225^Cleveland Clinic Lou Ruvo Center for Brain Health, Cleveland Clinic, Cleveland, OH, USA.

^226^Division of Neuroscience and Experimental Psychology, School of Biological Sciences, Faculty of Biology, Medicine and Health, University of Manchester, Manchester Academic Health Science Centre, Manchester, UK.

^227^Department of Pathology, University of Michigan, Ann Arbor, MI, USA.

^228^Institute of Epidemiology, Helmholtz Zentrum München, German Research Center for Environmental Health, Neuherberg, Munich, Germany.

^229^Helmholtz Center Munich, Institute of Epidemiology, Neuherberg, Munich, Germany.

^230^Ludwig-Maximilians University Chair of Epidemiology, Munich, Germany.

^231^Joint Biobank Munich and KORA Biobank, Baltimore, MD, USA.

^232^Department of Psychiatry, Johns Hopkins University, Baltimore, MD, USA.

^233^Human Genetics, Schools of Life Sciences and Medicine, University of Nottingham, Nottingham, UK.

^234^Department of Medicine-Pulmonary, New York University, New York, NY, USA.

^235^Department of Neurology, University of Miami, Miami, FL, USA.

^236^Department of Pathology, University of California, San Diego, La Jolla, CA, USA.

^237^Institute of Neurology, Catholic University of Sacred Hearth, Rome, Italy.

^238^School of Nursing Northwest Research Group on Aging, University of Washington, Seattle, WA, USA.

^239^Institute of Primary Care and Public Health, Cardiff University, University Hospital of Wales, Cardiff, UK.

^240^Department of Neurology, Northwestern University Feinberg School of Medicine, Chicago, IL, USA.

^241^Cambridge Institute of Public Health, University of Cambridge School of Clinical Medicine, Cambridge, UK.

^242^Weill Institute for Neurosciences, Memory and Aging Center, University of California, San Francisco, San Francisco, CA, USA.

^243^Institute of Social Medicine, Occupational Health and Public Health, University of Leipzig, Leipzig, Germany.

^244^Department of Pathology, University of Southern California, Los Angeles, CA, USA.

^245^Department of Pathology and Laboratory Medicine, University of California, Davis, Sacramento, CA, USA.

^246^Institute of Psychiatry, Psychology and Neuroscienceó, King's College London, London, UK.

^247^UK Dementia Research Institute, King's College London, London, UK.

^248^Department of Psychiatry and Psychotherapy, University Medical Center Goettingen, Goettingen, Germany.

^249^German Center for Neurodegenerative Diseases, Goettingen, Germany.

^250^IBiMED, Medical Sciences Department, University of Aveiro, Aveiro, Portugal.

^251^Molecular Genetics Laboratory-Hospital, University of Central Asturias, Oviedo, Spain.

^252^Department of Laboratory Medicine and Pathology, Mayo Clinic, Rochester, MN, USA.

^253^Molecular Genetics Section, Laboratory of Neurogenetics, National Institute on Aging, National Institutes of Health, Bethesda, MD, USA.

^254^Department of Neurology, University of California, Irvine, Irvine, CA, USA.

^255^Department of Neuroscience, Psychology, Drug Research and Child Health, University of Florence, Florence, Italy.

^256^Centro di Ricerca, Trasferimento e Alta Formazione DENOTHE, University of Florence, Florence, Italy.

^257^Department of Neurology, University of Colorado School of Medicine, Aurora, CO, USA.

^258^IRCCS Fondazione Don Carlo Gnocchi, Florence, Italy.

^259^Laboratory of Neuropsychiatry, IRCCS Santa Lucia Foundation, Rome, Italy.

^260^Division of Neuropsychiatry, Department of Psychiatry and Behavioral Sciences, Baylor College of Medicine, Houston, TX, USA.

^261^Alzheimer's Disease Center, New York University, New York, NY, USA.

^262^Oxford Healthy Aging Project, Clinical Trial Service Unit, University of Oxford, Oxford, UK.

^263^Department of Epidemiology, Columbia University, New York, NY, USA.

^264^Oxford Project to Investigate Memory and Ageing, University of Oxford, Nuffield Department of Clinical Neurosciences, John Radcliffe Hospital, Oxford, UK.

^265^Department of Neurology, Keck School of Medicine at the University of Southern California, Los Angeles, Los Angeles, CA, USA.

^266^Department of Neurology, University of Texas Southwestern Medical Center, Dallas, TX, USA.

^267^Population Health Sciences, Bristol Medical School, University of Bristol, Bristol, UK.

^268^Department of Pathology (Neuropathology), Rush University Medical Center, Chicago, IL, USA.

^269^Department of Psychiatry, University of Southern California, Los Angeles, CA, USA.

^270^Department of Pathology and Laboratory Medicine, University of Pennsylvania Perelman School of Medicine, Philadelphia, PA, USA.

^271^Department of Pathology, Johns Hopkins University, Baltimore, MD, USA.

^272^Sanders-Brown Center on Aging, Department of Neuroscience, University of Kentucky, Lexington, KY, USA.

^273^Department of Neurology, University of California, Los Angeles, Los Angeles, CA, USA.

^274^Department of Pathology and Laboratory Medicine, University of California, Los Angeles, Los Angeles, CA, USA.

^275^Taub Institute on Alzheimer's Disease and the Aging Brain, Department of Pathology, Columbia University, New York, NY, USA.

^276^Department of Psychiatry and Behavioral Sciences, Northwestern University Feinberg School of Medicine, Chicago, IL, USA.

^277^Department of Psychiatry and Behavioral Sciences, Duke University, Durham, NC, USA.

^278^Department of Genetics, University of North Carolina at Chapel Hill, Chapel Hill, NC, USA.

^279^Department of Human Genetics, Emory University, Atlanta, GA, USA.

^280^Department of Pathology, Oregon Health & Science University, Portland, OR, USA.

^281^National Institute of Neurological Disorders and Stroke, Bethesda, MD, USA.

^282^Gottfried Schatz Research Center for Cell Signaling, Metabolism and Aging, Division of Molecular Biology and Biochemistry, Medical University Graz, Graz, Austria.

^283^Gerontology and Geriatrics Research Laboratory, IRCCS Casa Sollievo della Sofferenza, San Giovanni Rotondo, Italy.

^284^Department Geriatric Care, Orthogeriatrics and Rehabilitation, Galliera Hospital, Genova, Italy.

^285^IdiPAZ, Instituto de Investigación Sanitaria la Paz, Madrid, Spain.

^286^Centro de Biologia Molecular Severo Ochoa (CSIC-UAM), Madrid, Spain.

^287^German Center for Neurodegenerative Diseases, Berlin, Germany.

^288^Instituto di Ricovero e Cura a Carattere Scientifico, Associazione Oasi Maria Santissima Srl, Troina, Italy.

^289^Center for Translational and Computational Neuroimmunology, Department of Neurology, Columbia University Medical Center, New York, NY, USA.

^290^Memory Unit, Neurology Department and Sant Pau Biomedical Research Institute, Hospital de la Santa Creu i Sant Pau, Autonomous University Barcelona, Barcelona, Spain.

^291^Somerset Partnership NHS Trust, Somerset, UK.

^292^The Wellcome Trust Sanger Institute, Hinxton, Cambridge, UK.

^293^Ronald M. Loeb Center for Alzheimer's Disease, Department of Neuroscience, Icahn School of Medicine at Mount Sinai, New York, NY, USA.

^294^University of Bristol Medical School, Learning & Research level 2, Southmead Hospital, Bristol, UK.

^295^Department of Neurology, Mayo Clinic, Jacksonville, FL, USA.

^296^Memory Research and Resources Center, CMRR de Bordeaux, Bordeaux, France.

^297^Utah State University, Logan, UT, USA.

^298^Department of Neurology, Erasmus MC University Medical Center, Rotterdam, the Netherlands.

^299^Departments of Radiology, Erasmus MC University Medical Center, Rotterdam, the Netherlands.

^300^Department of Research Rouvray Psychiatric Hospital, Sotteville-lès-Rouen, France.

^301^Department of Neurology, Medical University Graz, Graz, Austria.

^302^Center for Applied Genomics, Children's Hospital of Philadelphia, The Perelman School of Medicine, University of Pennsylvania, Philadelphia, PA, USA.

^303^Division of Human Genetics, Department of Pediatrics, The Perelman School of Medicine, University of Pennsylvania, Philadelphia, PA, USA.

^304^Department of Ophthalmology, Boston University School of Medicine, Boston University, Boston, MA, USA.

^305^Department of Epidemiology, Boston University School of Public Health, Boston, MA, USA.

^306^Department of Health Services, University of Washington, Seattle, WA, USA.

^307^Kaiser Permanente, Washington Health Research Institute, Seattle, WA, USA.

^308^Glenn Biggs Institute for Alzheimer's and Neurodegenerative Diseases, San Antonio, TX, USA.

^309^Centre Hospitalier Universitaire de Lille, Lille, France.

^310^Inserm, U1167, RID-AGE-Risk Factors and Molecular Determinants of Aging-Related Diseases, Lille, France. jean-charles.lambert@pasteur-lille.fr.

^311^Institut Pasteur de Lille, Lille, France. jean-charles.lambert@pasteur-lille.fr.

^312^Univ. Lille, U1167-Excellence Laboratory LabEx DISTALZ, Lille, France. jean-charles.lambert@pasteur-lille.fr.

^313^John P. Hussman Institute for Human Genomics, University of Miami Miller School of Medicine, Miami, FL, USA. mpericak@miami.edu.

**3. Identification of novel risk loci, causal insights, and heritable risk for Parkinson's disease: a meta-analysis of genome-wide association studies.**

Mike A Nalls^1^, Cornelis Blauwendraat^2^, Costanza L Vallerga^3^, Karl Heilbron^4^, Sara Bandres-Ciga^2^, Diana Chang^5^, Manuela Tan^6^, Demis A Kia^6^, Alastair J Noyce ^7^, Angli Xue^8^, Jose Bras ^9^, Emily Young^10^, Rainer von Coelln^11^, Javier Simón-Sánchez^12^, Claudia Schulte^12^, Manu Sharma^13^, Lynne Krohn^14^, Lasse Pihlstrøm^15^, Ari Siitonen^16^, Hirotaka Iwaki^17^, Hampton Leonard^18^, Faraz Faghri^19^, J Raphael Gibbs^2^, Dena G Hernandez^2^, Sonja W Scholz^20^, Juan A Botia^21^, Maria Martinez^22^, Jean-Christophe Corvol^23^, Suzanne Lesage^23^, Joseph Jankovic^10^, Lisa M Shulman^11^, Margaret Sutherland^24^, Pentti Tienari^25^, Kari Majamaa^16^, Mathias Toft^26^, Ole A Andreassen^27^, Tushar Bangale^5^, Alexis Brice^23^, Jian Yang^8^, Ziv Gan-Or^28^, Thomas Gasser^12^, Peter Heutink^12^, Joshua M Shulman^29^, Nicholas W Wood^6^, David A Hinds^4^, John A Hardy^30^, Huw R Morris^31^, Jacob Gratten^32^, Peter M Visscher^8^, Robert R Graham^5^, Andrew B Singleton^2^, 23andMe Research Team; System Genomics of Parkinson's Disease Consortium; International Parkinson's Disease Genomics Consortium

^1^Laboratory of Neurogenetics, National Institute on Aging, National Institutes of Health, Bethesda, MD, USA; Data Tecnica International, Glen Echo, MD, USA. Electronic address: mike@datatecnica.com.

^2^Laboratory of Neurogenetics, National Institute on Aging, National Institutes of Health, Bethesda, MD, USA.

^3^Institute for Molecular Bioscience, The University of Queensland, Brisbane, QLD, Australia.

^4^23andMe, Sunnyvale, CA, USA.

^5^Department of Human Genetics, Genentech, South San Francisco, CA, USA.

^6^Department of Molecular Neuroscience, UCL Queen Square Institute of Neurology, London, UK; Department of Clinical and Movement Neuroscience and UCL Movement Disorders Centre, UCL Queen Square Institute of Neurology, London, UK.

^7^Department of Molecular Neuroscience, UCL Queen Square Institute of Neurology, London, UK; Preventive Neurology Unit, Wolfson Institute of Preventive Medicine, Queen Mary University of London, London, UK.

^8^Queensland Brain Institute, The University of Queensland, Brisbane, QLD, Australia; Institute for Molecular Bioscience, The University of Queensland, Brisbane, QLD, Australia.

^9^Department of Neurodegenerative Diseases, UCL Queen Square Institute of Neurology, London, UK; Center for Neurodegenerative Science, Van Andel Research Institute, Grand Rapids, MI, USA.

^10^Department of Neurology, Baylor College of Medicine, Houston, TX, USA.

^11^Department of Neurology, University of Maryland School of Medicine, Baltimore, MD, USA.

^12^Department for Neurodegenerative Diseases, Hertie Institute for Clinical Brain Research, University of Tübingen, Tübingen, Germany; German Center for Neurodegenerative Diseases, Tübingen, Germany.

^13^Centre for Genetic Epidemiology, Institute for Clinical Epidemiology and Applied Biometry, University of Tübingen, Tübingen, Germany.

^14^Department of Human Genetics, McGill University, Montreal, QC, Canada; Montreal Neurological Institute, McGill University, Montreal, QC, Canada.

^15^Department of Neurology, Oslo University Hospital, Oslo, Norway.

^16^Institute of Clinical Medicine, Department of Neurology, University of Oulu, Oulu, Finland; Department of Neurology and Medical Research Center, Oulu University Hospital, Oulu, Finland.

^17^Laboratory of Neurogenetics, National Institute on Aging, National Institutes of Health, Bethesda, MD, USA; Data Tecnica International, Glen Echo, MD, USA; The Michael J Fox Foundation, New York, NY, USA.

^18^Laboratory of Neurogenetics, National Institute on Aging, National Institutes of Health, Bethesda, MD, USA; Data Tecnica International, Glen Echo, MD, USA.

^19^Laboratory of Neurogenetics, National Institute on Aging, National Institutes of Health, Bethesda, MD, USA; Department of Computer Science, University of Illinois Urbana-Champaign, Champaign, IL, USA.

^20^National Institute of Neurological Disorders and Stroke, National Institutes of Health, Bethesda, MD, USA; Department of Neurology, Johns Hopkins University Medical Center, Baltimore, MD, USA.

^21^Department of Molecular Neuroscience, UCL Queen Square Institute of Neurology, London, UK; Departamento de Ingeniería de la Información y las Comunicaciones, Universidad de Murcia, Spain.

^22^Institut national de la santé et de la recherche médicale Unité mixte de recherche 1220, Toulouse, France; Paul Sabatier University, Toulouse, France.

^23^Institut national de la santé et de la recherche médicale U1127, CNRS UMR 7225, Paris, France; Sorbonne Université centre national de la recherche médicale, unité mixte de recherche 1127, Paris, France; Assistance Publique Hôpitaux de Paris, Paris, France; Institut du Cerveau et de la Moelle épinière, Paris, France.

^24^National Institute of Neurological Disorders and Stroke, National Institutes of Health, Bethesda, MD, USA.

^25^Clinical Neurosciences, Neurology, University of Helsinki, Helsinki, Finland; Helsinki University Hospital, Helsinki, Finland.

^26^Department of Neurology, Oslo University Hospital, Oslo, Norway; Institute of Clinical Medicine, University of Oslo, Oslo, Norway.

^27^Division of Mental Health and Addiction, Oslo University Hospital, Oslo, Norway; Jebsen Centre for Psychosis Research, University of Oslo, Oslo, Norway.

^28^Department of Human Genetics, McGill University, Montreal, QC, Canada; Montreal Neurological Institute, McGill University, Montreal, QC, Canada; Department of Neurology and Neurosurgery, McGill University, Montreal, QC, Canada.

^29^Department of Neurology, Baylor College of Medicine, Houston, TX, USA; Department of Molecular and Human Genetics, Baylor College of Medicine, Houston, TX, USA; Department of Neuroscience, Baylor College of Medicine, Houston, TX, USA; Jan and Dan Duncan Neurological Research Institute, Texas Children's Hospital, Houston, TX, USA.

^30^Department of Molecular Neuroscience, UCL Queen Square Institute of Neurology, London, UK.

^31^Department of Clinical and Movement Neuroscience and UCL Movement Disorders Centre, UCL Queen Square Institute of Neurology, London, UK.

^32^Institute for Molecular Bioscience, The University of Queensland, Brisbane, QLD, Australia; Mater Research Institute, The University of Queensland, Brisbane, QLD, Australia.

**4. Genome-wide Analyses Identify KIF5A as a Novel ALS Gene**

Aude Nicolas ^1^, Kevin P Kenna ^2^, Alan E Renton ^3^, Nicola Ticozzi ^4^, Faraz Faghri ^5^, Ruth Chia ^1^, Janice A Dominov ^2^, Brendan J Kenna ^2^, Mike A Nalls ^6^, Pamela Keagle ^2^, Alberto M Rivera 1, Wouter van Rheenen 7, Natalie A Murphy 1, Joke J F A van Vugt 7, Joshua T Geiger ^8^, Rick A Van der Spek ^7^, Hannah A Pliner ^1^, Shankaracharya ^2^, Bradley N Smith ^9^, Giuseppe Marangi ^10^, Simon D Topp ^9^, Yevgeniya Abramzon ^11^, Athina Soragia Gkazi ^9^, John D Eicher ^12^, Aoife Kenna ^2^, ITALSGEN Consortium; Gabriele Mora ^13^, Andrea Calvo ^14^, Letizia Mazzini ^15^, Nilo Riva ^16^, Jessica Mandrioli ^17^, Claudia Caponnetto ^18^, Stefania Battistini ^19^, Paolo Volanti ^13^, Vincenzo La Bella ^20^, Francesca L Conforti ^21^, Giuseppe Borghero ^22^, Sonia Messina ^23^, Isabella L Simone ^24^, Francesca Trojsi ^25^, Fabrizio Salvi ^26^, Francesco O Logullo ^27^, Sandra D'Alfonso ^28^, Lucia Corrado ^28^, Margherita Capasso ^29^, Luigi Ferrucci ^30^, Genomic Translation for ALS Care (GTAC) Consortium; Cristiane de Araujo Martins Moreno ^31^, Sitharthan Kamalakaran ^32^, David B Goldstein ^32^, ALS Sequencing Consortium; Aaron D Gitler ^33^, Tim Harris ^34^, Richard M Myers ^35^, NYGC ALS Consortium; Hemali Phatnani ^36^, Rajeeva Lochan Musunuri ^37^, Uday Shankar Evani ^37^, Avinash Abhyankar ^37^, Michael C Zody ^37^, Answer ALS Foundation; Julia Kaye ^38^, Steven Finkbeiner ^39^, Stacia K Wyman ^38^, Alex LeNail ^40^, Leandro Lima ^38^, Ernest Fraenkel ^41^, Clive N Svendsen ^42^, Leslie M Thompson ^43^, Jennifer E Van Eyk ^44^, James D Berry ^45^, Timothy M Miller ^46^, Stephen J Kolb ^47^, Merit Cudkowicz ^45^, Emily Baxi ^48^, Clinical Research in ALS and Related Disorders for Therapeutic Development (CReATe) Consortium; Michael Benatar ^49^, J Paul Taylor ^50^, Evadnie Rampersaud ^51^, Gang Wu ^51^, Joanne Wuu ^49^, SLAGEN Consortium; Giuseppe Lauria 52, Federico Verde 53, Isabella Fogh 54, Cinzia Tiloca ^53^, Giacomo P Comi ^55^, Gianni Sorarù ^56^, Cristina Cereda ^57^, French ALS Consortium; Philippe Corcia ^58^, Hannu Laaksovirta ^59^, Liisa Myllykangas ^60^, Lilja Jansson ^59^, Miko Valori ^59^, John Ealing ^61^, Hisham Hamdalla ^61^, Sara Rollinson ^62^, Stuart Pickering-Brown ^62^, Richard W Orrell ^63^, Katie C Sidle ^64^, Andrea Malaspina ^65^, John Hardy ^64^, Andrew B Singleton ^66^, Janel O Johnson ^1^, Sampath Arepalli ^67^, Peter C Sapp ^2^, Diane McKenna-Yasek ^2^, Meraida Polak ^68^, Seneshaw Asress ^68^, Safa Al-Sarraj ^9^, Andrew King ^9^, Claire Troakes ^9^, Caroline Vance ^9^, Jacqueline de Belleroche ^69^, Frank Baas ^70^, Anneloor L M A Ten Asbroek ^71^, José Luis Muñoz-Blanco ^72^, Dena G Hernandez ^67^, Jinhui Ding ^73^, J Raphael Gibbs ^73^, Sonja W Scholz 74, Mary Kay Floeter ^75^, Roy H Campbell ^76^, Francesco Landi ^77^, Robert Bowser ^78^, Stefan M Pulst ^79^, John M Ravits ^80^, Daniel J L MacGowan ^81^, Janine Kirby ^82^, Erik P Pioro ^83^, Roger Pamphlett ^84^, James Broach ^85^, Glenn Gerhard ^86^, Travis L Dunckley ^87^, Christopher B Brady ^88^, Neil W Kowall ^89^, Juan C Troncoso ^90^, Isabelle Le Ber ^91^, Kevin Mouzat ^92^, Serge Lumbroso ^92^, Terry D Heiman-Patterson ^93^, Freya Kamel ^94^, Ludo Van Den Bosch ^95^, Robert H Baloh ^96^, Tim M Strom ^97^, Thomas Meitinger ^98^, Aleksey Shatunov ^9^, Kristel R Van Eijk ^7^, Mamede de Carvalho ^99^, Maarten Kooyman ^100^, Bas Middelkoop ^7^, Matthieu Moisse ^95^, Russell L McLaughlin ^101^, Michael A Van Es ^7^, Markus Weber ^102^, Kevin B Boylan ^103^, Marka Van Blitterswijk ^104^, Rosa Rademakers ^104^, Karen E Morrison ^105^, A Nazli Basak ^106^, Jesús S Mora ^107^, Vivian E Drory ^108^, Pamela J Shaw ^82^, Martin R Turner ^109^, Kevin Talbot ^109^, Orla Hardiman ^110^, Kelly L Williams ^111^, Jennifer A Fifita ^111^, Garth A Nicholson ^112^, Ian P Blair ^111^, Guy A Rouleau ^113^, Jesús Esteban-Pérez ^114^, Alberto García-Redondo ^114^, Ammar Al-Chalabi ^9^, Project MinE ALS Sequencing Consortium; Ekaterina Rogaeva ^115^, Lorne Zinman ^116^, Lyle W Ostrow ^48^, Nicholas J Maragakis ^48^, Jeffrey D Rothstein ^48^, Zachary Simmons ^117^, Johnathan Cooper-Knock ^82^, Alexis Brice ^91^, Stephen A Goutman ^118^, Eva L Feldman ^118^, Summer B Gibson ^79^, Franco Taroni ^119^, Antonia Ratti ^4^, Cinzia Gellera ^119^, Philip Van Damme ^12^0, Wim Robberecht ^120^, Pietro Fratta ^121^, Mario Sabatelli ^122^, Christian Lunetta ^123^, Albert C Ludolph ^124^, Peter M Andersen ^125^, Jochen H Weishaupt ^124^, William Camu ^126^, John Q Trojanowski ^127^, Vivianna M Van Deerlin ^127^, Robert H Brown Jr ^2^, Leonard H van den Berg ^7^, Jan H Veldink ^7^, Matthew B Harms ^31^, Jonathan D Glass ^68^, David J Stone ^128^, Pentti Tienari ^59^, Vincenzo Silani ^4^, Adriano Chiò ^129^, Christopher E Shaw ^9^, Bryan J Traynor ^130^, John E Landers ^131^

^1^Neuromuscular Diseases Research Section, Laboratory of Neurogenetics, National Institute on Aging, NIH, Porter Neuroscience Research Center, Bethesda, MD 20892, USA.

^2^Department of Neurology, University of Massachusetts Medical School, Worcester, MA 01605, USA.

^3^Neuromuscular Diseases Research Section, Laboratory of Neurogenetics, National Institute on Aging, NIH, Porter Neuroscience Research Center, Bethesda, MD 20892, USA; Department of Neuroscience, Icahn School of Medicine at Mount Sinai, New York, NY 10029, USA; Ronald M. Loeb Center for Alzheimer's Disease, Icahn School of Medicine at Mount Sinai, New York, NY 10029, USA.

^4^Department of Neurology and Laboratory of Neuroscience, IRCCS Istituto Auxologico Italiano, Milan, Italy; Department of Pathophysiology and Transplantation, "Dino Ferrari" Center - Università degli Studi di Milano, Milan 20122, Italy.

^5^Molecular Genetics Section, Laboratory of Neurogenetics, National Institute on Aging, NIH, Porter Neuroscience Research Center, Bethesda, MD 20892, USA; Department of Computer Science, University of Illinois at Urbana-Champaign, Urbana, IL, USA.

^6^Molecular Genetics Section, Laboratory of Neurogenetics, National Institute on Aging, NIH, Porter Neuroscience Research Center, Bethesda, MD 20892, USA; Data Tecnica International, Glen Echo, MD, USA.

^7^Department of Neurology, Brain Center Rudolf Magnus, University Medical Center Utrecht, Utrecht, the Netherlands.

^8^Neurodegenerative Diseases Research Unit, National Institute of Neurological Disorders and Stroke, NIH, Bethesda, MD 20892, USA.

^9^Maurice Wohl Clinical Neuroscience Institute, Department of Basic and Clinical Neuroscience, King's College London, London SE5 9RS, UK.

^10^Neuromuscular Diseases Research Section, Laboratory of Neurogenetics, National Institute on Aging, NIH, Porter Neuroscience Research Center, Bethesda, MD 20892, USA; Institute of Genomic Medicine, Catholic University, Roma, Italy.

^11^Neuromuscular Diseases Research Section, Laboratory of Neurogenetics, National Institute on Aging, NIH, Porter Neuroscience Research Center, Bethesda, MD 20892, USA; Sobell Department of Motor Neuroscience and Movement Disorders, University College London, Institute of Neurology, London, UK.

^12^Genetics and Pharmacogenomics, MRL, Merck & Co., Inc., Boston, MA 02115, USA.

^13^ALS Center, Salvatore Maugeri Foundation, IRCCS, Mistretta, Messina, Italy.

^14^"Rita Levi Montalcini" Department of Neuroscience, University of Turin, Turin, Italy.

^15^"Maggiore della Carità" University Hospital, Novara, Italy.

^16^Department of Neurology, Institute of Experimental Neurology, Division of Neuroscience, San Raffaele Scientific Institute, Milan, Italy.

^17^Department of Neuroscience, St. Agostino Estense Hospital, Azienda Ospedaliero Universitaria di Modena, Modena, Italy.

^18^Department of Neurosciences, Ophthalmology, Genetics, Rehabilitation, Maternal and Child Health, Ospedale Policlinico San Martino, Genoa, Italy.

^19^Department of Medical, Surgical and Neurological Sciences, University of Siena, Siena, Italy.

20ALS Clinical Research Center, University of Palermo, Palermo, Italy.

^21^Institute of Neurological Sciences, National Research Council, Mangone, Cosenza, Italy.

^22^Department of Neurology, Azienda Universitario Ospedaliera di Cagliari and University of Cagliari, Cagliari, Italy.

^23^Department of Clinical and Experimental Medicine, University of Messina and Nemo Sud Clinical Center for Neuromuscular Diseases, Aurora Foundation, Messina, Italy.

^24^Department of Basic Medical Sciences, Neurosciences and Sense Organs, University of Bari, Bari, Italy.

^25^Department of Medical, Surgical, Neurological, Metabolic and Aging Sciences, University of Campania "Luigi Vanvitelli," Naples, Italy.

^26^"Il Bene" Center for Immunological and Rare Neurological Diseases at Bellaria Hospital, IRCCS, Istituto delle Scienze Neurologiche, Bologna, Italy.

^27^Neurological Clinic, Marche Polytechnic University, Ancona, Italy.

^28^Department of Health Sciences, University of Eastern Piedmont, Novara, Italy.

^29^Department of Neurology, University of Chieti, Chieti, Italy.

^30^Longitudinal Studies Section, Clinical Research Branch, National Institute on Aging, NIH, Baltimore, MD 21224, USA.

^31^Department of Neurology, Columbia University, New York, NY 10032, USA.

^32^Institute for Genomic Medicine, Columbia University, New York, NY 10032, USA.

^33^Department of Genetics, Stanford University School of Medicine, Stanford, CA 94305, USA.

^34^Bioverativ, 225 2nd Avenue, Waltham, MA 02145, USA.

^35^HudsonAlpha Institute for Biotechnology, Huntsville, AL 35806, USA.

^36^Center for Genomics of Neurodegenerative Diseases (CGND), New York Genome Center, New York, NY, USA.

^37^Computational Biology, New York Genome Center, New York, NY, USA.

^38^Gladstone Institute of Neurological Disease, San Francisco, CA, USA.

^39^Gladstone Institute of Neurological Disease, San Francisco, CA, USA; Departments of Neurology and Physiology, University of California, San Francisco, San Francisco, CA, USA.

^40^Department of Biological Engineering, Massachusetts Institute of Technology, 77 Massachusetts Avenue, Cambridge, MA 02139, USA.

^41^Department of Biological Engineering, Massachusetts Institute of Technology, 77 Massachusetts Avenue, Cambridge, MA 02139, USA; Broad Institute, 415 Main Street, Cambridge, MA 02142, USA.

^42^Board of Governors Regenerative Medicine Institute, Cedars-Sinai Medical Center, Los Angeles, CA 90048, USA; Department of Biomedical Sciences, Cedars-Sinai Medical Center, Los Angeles, CA 90048, USA.

^43^Department of Neurobiology and Behavior, Institute of Memory Impairment and Neurological Disorders, University of California, Irvine, Irvine, CA 92697, USA; Department of Psychiatry and Human Behavior, Institute of Memory Impairment and Neurological Disorders, University of California, Irvine, Irvine, CA 92697, USA.

^44^The Heart Institute and Department of Medicine, Cedars-Sinai Medical Center, Los Angeles, CA, USA.

^45^Harvard Medical School, Department of Neurology, Massachusetts General Hospital (MGH), Boston, MA, USA; Neurological Clinical Research Institute (NCRI), Massachusetts General Hospital, Boston, MA, USA.

^46^Department of Neurology, Washington University School of Medicine, St. Louis, MO, USA.

^47^Department of Neurology, The Ohio State University Wexner Medical Center, Columbus, OH, USA.

^48^Department of Neurology, Johns Hopkins University, Baltimore, MD 21287, USA.

^49^Department of Neurology, University of Miami, Miami, FL 33136, USA.

^50^Howard Hughes Medical Institute, Chevy Chase, MD 20815, USA; Department of Cell and Molecular Biology, St. Jude Children's Research Hospital, Memphis, TN 38105, USA.

^51^Department of Computational Biology, St. Jude Children's Research Hospital, Memphis, TN 38105, USA.

^52^3rd Neurology Unit, Motor Neuron Diseases Center, Fondazione IRCCS Istituto Neurologico "Carlo Besta," and Department of Biomedical and Clinical Sciences "Luigi Sacco," University of Milan, Milan, Italy.

^53^Department of Neurology and Laboratory of Neuroscience, IRCCS Istituto Auxologico Italiano, Milan, Italy.

^54^Department of Neurology and Laboratory of Neuroscience, IRCCS Istituto Auxologico Italiano, Milan, Italy; Maurice Wohl Clinical Neuroscience Institute, Department of Basic and Clinical Neuroscience, King's College London, London SE5 9RS, UK.

^55^Neurology Unit, IRCCS Foundation Ca' Granda Ospedale Maggiore Policlinico, Milan, Italy.

^56^Department of Neurosciences, University of Padova, Padova, Italy.

^57^Genomic and Post-Genomic Center, IRCCS Mondino Foundation, Pavia, Italy.

^58^ALS Center, CHU Bretonneau, Tours University, Tours, France.

^59^Department of Neurology, Helsinki University Hospital and Molecular Neurology Programme, Biomedicum, University of Helsinki, Helsinki FIN-02900, Finland.

^60^Department of Pathology, University of Helsinki and Helsinki University Hospital, Helsinki, Finland.

^61^Greater Manchester Neurosciences Centre, Salford Royal NHS Foundation Trust, Salford M6 8HD, UK.

^62^Faculty of Human and Medical Sciences, University of Manchester, Manchester M13 9PT, UK.

^63^Department of Clinical Neuroscience, Institute of Neurology, University College London, London NW3 2PG, UK.

^64^Department of Molecular Neuroscience and Reta Lila Weston Laboratories, Institute of Neurology, University College London, Queen Square House, London WC1N 3BG, UK.

^65^Centre for Neuroscience and Trauma, Blizard Institute, Queen Mary University of London, NorthEast London and Essex Regional Motor Neuron Disease Care Centre, London E1 2AT, UK.

^66^Molecular Genetics Section, Laboratory of Neurogenetics, National Institute on Aging, NIH, Porter Neuroscience Research Center, Bethesda, MD 20892, USA.

^67^Genomics Technology Group, Laboratory of Neurogenetics, National Institute on Aging, NIH, Porter Neuroscience Research Center, Bethesda, MD 20892, USA.

^68^Department of Neurology, Emory University School of Medicine, Atlanta, GA 30322, USA.

^69^Division of Brain Sciences, Department of Medicine, Imperial College London, London W120NN, UK.

^70^Department of Clinical Genetics, Leiden University Medical Center, Leiden, the Netherlands.

^71^Department of Neurogenetics and Neurology, Academic Medical Centre, Amsterdam, the Netherlands.

^72^ALS-Neuromuscular Unit, Hospital General Universitario Gregorio Marañón, IISGM, Madrid, Spain.

^73^Computational Biology Group, Laboratory of Neurogenetics, National Institute on Aging, NIH, Porter Neuroscience Research Center, Bethesda, MD 20892, USA.

^74^Neurodegenerative Diseases Research Unit, National Institute of Neurological Disorders and Stroke, NIH, Bethesda, MD 20892, USA; Department of Neurology, Johns Hopkins University, Baltimore, MD 21287, USA.

^75^Motor Neuron Disorders Unit, National Institute of Neurological Disorders and Stroke, NIH, Bethesda, MD 20892, USA.

^76^Department of Computer Science, University of Illinois at Urbana-Champaign, Urbana, IL, USA.

^77^Center for Geriatric Medicine, Department of Geriatrics, Neurosciences and Orthopedics, Catholic University of Sacred Heart, Rome 00168, Italy.

^78^Division of Neurology, Barrow Neurological Institute, Phoenix, AZ, USA.

^79^Department of Neurology, University of Utah School of Medicine, Salt Lake City, UT, USA.

^80^Department of Neuroscience, University of California, San Diego, La Jolla, CA, USA.

^81^Mount Sinai Beth Israel Hospital, Mount Sinai School of Medicine, New York, NY, USA.

^82^Sheffield Institute for Translational Neuroscience (SITraN), University of Sheffield, Sheffield, UK.

^83^Department of Neurology, Neuromuscular Center, Neurological Institute, Cleveland Clinic, Cleveland, OH, USA.

^84^Discipline of Pathology, Brain and Mind Centre, The University of Sydney, 94 Mallett Street, Camperdown, NSW 2050, Australia.

^85^Department of Biochemistry, Penn State College of Medicine, Hershey, PA, USA.

^86^Department of Pathology, Penn State College of Medicine, Hershey, PA, USA.

^87^Neurogenomics Division, Translational Genomics Research Institute, Phoenix, AZ, USA.

^88^Research and Development Service, Veterans Affairs Boston Healthcare System, Boston, MA, USA; Department of Neurology, Program in Behavioral Neuroscience, Boston University School of Medicine, Boston, MA, USA.

^89^Neurology Service, VA Boston Healthcare System and Boston University Alzheimer's Disease Center, Boston, MA 02130, USA.

^90^Departments of Pathology and Neurology, Johns Hopkins University School of Medicine, Baltimore, MD 21205, USA.

^91^Sorbonne Universités, UPMC Univ Paris 06, Inserm, CNRS, Institut du Cerveau et la Moelle (ICM), Assistance Publique Hôpitaux de Paris (AP-HP) - Hôpital Pitié-Salpêtrière, Paris, France.

^92^INM, University Montpellier, Montpellier, France; Department of Biochemistry, CHU Nîmes, Nîmes, France.

^93^Department of Neurology, Drexel University College of Medicine, Philadelphia, PA, USA; Department of Neurology, Lewis Katz School of Medicine, Temple University, Philadelphia, PA, USA.

^94^Epidemiology Branch, National Institute of Environmental Health Sciences, Durham, NC 27709, USA.

^95^KU Leuven - University of Leuven, Department of Neurosciences, Experimental Neurology and Leuven Research Institute for Neuroscience and Disease (LIND), B-3000 Leuven, Belgium; VIB, Center for Brain and Disease Research, Laboratory of Neurobiology, Leuven, Belgium.

^96^Department of Neurology, Cedars-Sinai Medical Center, Los Angeles, CA, USA.

^97^Institute of Human Genetics, Technische Universität München, Munich, Germany; Institute of Human Genetics, Helmholtz Zentrum München, German Research Center for Environmental Health, Neuherberg, Germany.

^98^Institute of Human Genetics, Technische Universität München, Munich, Germany; Institute of Human Genetics, Helmholtz Zentrum München, German Research Center for Environmental Health, Neuherberg, Germany; Munich Cluster for Systems Neurology (SyNergy), Munich, Germany.

^99^Institute of Physiology, Institute of Molecular Medicine, Faculty of Medicine, University of Lisbon, Lisbon, Portugal; Department of Neurosciences, Hospital de Santa Maria-CHLN, Lisbon, Portugal.

^100^SURFsara, Amsterdam, the Netherlands.

^101^Population Genetics Laboratory, Smurfit Institute of Genetics, Trinity College Dublin, Dublin, Republic of Ireland.

^102^Neuromuscular Diseases Center/ALS Clinic, Kantonsspital St. Gallen, St. Gallen, Switzerland.

^103^Department of Neurology, Mayo Clinic Florida, Jacksonville, FL 32224, USA.

^104^Department of Neuroscience, Mayo Clinic, Jacksonville, FL, USA.

^105^Faculty of Medicine, University of Southampton, Southampton, UK.

^106^Suna and Inan Kırac Foundation, Neurodegeneration Research Laboratory, Bogazici University, Istanbul, Turkey.

^107^ALS Unit/Neurology, Hospital San Rafael, Madrid, Spain.

^108^Department of Neurology, Tel-Aviv Sourasky Medical Centre, Tel-Aviv, Israel.

^109^Nuffield Department of Clinical Neurosciences, University of Oxford, Oxford, UK.

^110^Academic Unit of Neurology, Trinity Biomedical Sciences Institute, Trinity College Dublin, Dublin, Republic of Ireland.

^111^Centre for MND Research, Faculty of Medicine and Health Sciences, Macquarie University, Sydney, NSW 2109, Australia.

^112^Centre for MND Research, Faculty of Medicine and Health Sciences, Macquarie University, Sydney, NSW 2109, Australia; ANZAC Research Institute, Concord Hospital, University of Sydney, Sydney, NSW 2139, Australia.

^113^Montreal Neurological Institute, Department of Neurology and Neurosurgery, McGill University, Montreal, QC, Canada.

^114^Unidad de ELA, Instituto de Investigación Hospital 12 de Octubre de Madrid, SERMAS, and Centro de Investigación Biomédica en Red de Enfermedades Raras (CIBERER U-723), Madrid, Spain.

^115^Tanz Centre for Research of Neurodegenerative Diseases, Division of Neurology, Department of Medicine, University of Toronto, Toronto, ON M5S 3H2, Canada.

^116^Division of Neurology, Department of Internal Medicine, Sunnybrook Health Sciences Centre, University of Toronto, Toronto, ON M4N 3M5, Canada.

^117^Department of Neurology, Penn State Hershey Medical Center, Hershey, PA, USA.

^118^Department of Neurology, University of Michigan, Ann Arbor, MI, USA.

^119^Unit of Genetics of Neurodegenerative and Metabolic Diseases, Fondazione IRCCS Istituto Neurologico "Carlo Besta," Milan 20133, Italy.

^120^KU Leuven - University of Leuven, Department of Neurosciences, Experimental Neurology and Leuven Research Institute for Neuroscience and Disease (LIND), B-3000 Leuven, Belgium; VIB, Center for Brain and Disease Research, Laboratory of Neurobiology, Leuven, Belgium; University Hospitals Leuven, Department of Neurology, Leuven, Belgium.

^121^Sobell Department of Motor Neuroscience and Movement Disorders, University College London, Institute of Neurology, London, UK.

^122^Centro Clinico NeMO, Institute of Neurology, Catholic University, Largo F. Vito 1, 00168 Rome, Italy.

^123^NEuroMuscular Omnicenter (NEMO), Serena Onlus Foundation, Milan, Italy.

^124^Neurology Department, Ulm University, Albert-Einstein-Allee 11, 89081 Ulm, Germany.

^125^Department of Pharmacology and Clinical Neuroscience, Umeå University, Umeå SE-90185, Sweden.

^126^ALS Center, CHU Gui de Chauliac, University of Montpellier, Montpellier, France.

^127^Department of Pathology and Laboratory Medicine, University of Pennsylvania, Philadelphia, PA, USA.

^128^Genetics and Pharmacogenomics, MRL, Merck & Co., Inc., West Point, PA 19486, USA.

^129^"Rita Levi Montalcini" Department of Neuroscience, University of Turin, Turin, Italy; Neuroscience Institute of Torino, Turin 10124, Italy.

^130^Neuromuscular Diseases Research Section, Laboratory of Neurogenetics, National Institute on Aging, NIH, Porter Neuroscience Research Center, Bethesda, MD 20892, USA; Department of Neurology, Johns Hopkins University, Baltimore, MD 21287, USA. Electronic address: bryan.traynor@nih.gov.

^131^Department of Neurology, University of Massachusetts Medical School, Worcester, MA 01605, USA. Electronic address: [john.landers@umassmed.edu](mailto:john.landers@umassmed.edu).

## 5. Members of the MEGASTROKE Consortium

Rainer Malik^1^, Ganesh Chauhan^2^, Matthew Traylor^3^, Muralidharan Sargurupremraj^4,5^, Yukinori Okada^6,7,8^, Aniket Mishra^4,5^, Loes Rutten-Jacobs ^3^, Anne-Katrin Giese ^9^, Sander W van der Laan ^10^, Solveig Gretarsdottir ^11^, Christopher D Anderson ^12,13,14,14^, Michael Chong ^15^, Hieab HH Adams ^16,17^, Tetsuro Ago ^18^, Peter Almgren ^19^, Philippe Amouyel ^20,21^, Hakan Ay ^22,13^, Traci M Bartz ^23^, Oscar R Benavente ^24^, Steve Bevan ^25^, Giorgio B Boncoraglio ^26^, Robert D Brown, Jr. ^27^, Adam S Butterworth ^28,29^, Caty Carrera ^30,31^, Cara L Carty ^32,33^, Daniel I Chasman ^34,35^, Wei-Min Chen ^36^, John W Cole ^37^, Adolfo Correa ^38^, Ioana Cotlarciuc ^39^, Carlos Cruchaga ^40,41^, John Danesh ^28,42,43,44^, Paul IW de Bakker ^45,46^, Anita L DeStefano ^47,48^, Marcel den Hoed ^49^, Qing Duan ^50^, Stefan T Engelter ^51,52^, Guido J Falcone ^53,54^, Rebecca F Gottesman ^55^, Raji P Grewal ^56^, Vilmundur Gudnason ^57,58^, Stefan Gustafsson ^59^, Jeffrey Haessler ^60^, Tamara B Harris ^61^, Ahamad Hassan ^62^, Aki S Havulinna ^63,64^, Susan R Heckbert ^65^, Elizabeth G Holliday ^66,67^, George Howard ^68^, Fang-Chi Hsu ^69^, Hyacinth I Hyacinth ^70^, M Arfan Ikram ^16^, Erik Ingelsson ^71,72^, Marguerite R Irvin ^73^, Xueqiu Jian ^74^, Jordi Jiménez-Conde ^75^, Julie A Johnson ^76,77^, J Wouter Jukema ^78^, Masahiro Kanai ^6,7,79^, Keith L Keene ^80,81^, Brett M Kissela ^82^, Dawn O Kleindorfer ^82^, Charles Kooperberg ^60^, Michiaki Kubo ^83^, Leslie A Lange ^84^, Carl D Langefeld ^85^, Claudia Langenberg ^86^, Lenore J Launer ^87^, Jin-Moo Lee ^88^, Robin Lemmens ^89,90^, Didier Leys ^91^, Cathryn M Lewis ^92,93^, Wei-Yu Lin ^28,94^, Arne G Lindgren ^95,96^, Erik Lorentzen ^97^, Patrik K Magnusson ^98^, Jane Maguire ^99^, Ani Manichaikul ^36^, Patrick F McArdle ^100^, James F Meschia ^101^, Braxton D Mitchell ^100,102^, Thomas H Mosley ^103,104^, Michael A Nalls ^105,106^, Toshiharu Ninomiya ^107^, Martin J O'Donnell ^15,108^, Bruce M Psaty ^109,110,111,112^, Sara L Pulit ^113,45^, Kristiina Rannikmäe ^114,115^, Alexander P Reiner ^65,116^, Kathryn M Rexrode ^117^, Kenneth Rice ^118^, Stephen S Rich ^36^, Paul M Ridker ^34,35^, Natalia S Rost ^9,13^, Peter M Rothwell ^119^, Jerome I Rotter ^120,121^, Tatjana Rundek ^122^, Ralph L Sacco ^122^, Saori Sakaue ^7,123^, Michele M Sale ^124^, Veikko Salomaa ^63^, Bishwa R Sapkota ^125^, Reinhold Schmidt ^126^, Carsten O Schmidt ^127^, Ulf Schminke ^128^, Pankaj Sharma ^39^, Agnieszka Slowik ^129^, Cathie LM Sudlow ^114,115^, Christian Tanislav ^130^, Turgut Tatlisumak ^131,132^, Kent D Taylor ^120,121^, Vincent NS Thijs ^133,134^, Gudmar Thorleifsson ^11^, Unnur Thorsteinsdottir ^11^, Steffen Tiedt ^1^, Stella Trompet ^135^, Christophe Tzourio ^5,136,137^, Cornelia M van Duijn ^138,139^, Matthew Walters ^140^, Nicholas J Wareham ^86^, Sylvia Wassertheil-Smoller ^141^, James G Wilson ^142^, Kerri L Wiggins ^109^, Qiong Yang ^47^, Salim Yusuf ^15^, Najaf Amin ^16^, Hugo S Aparicio ^185,48^, Donna K Arnett ^186^, John Attia ^187^, Alexa S Beiser ^47,48^, Claudine Berr ^188^, Julie E Buring ^34,35^, Mariana Bustamante ^189^, Valeria Caso ^190^, Yu-Ching Cheng ^191^, Seung Hoan Choi ^192,48^, Ayesha Chowhan ^185,48^, Natalia Cullell ^31^, Jean-François Dartigues ^193,194^, Hossein Delavaran ^95,96^, Pilar Delgado ^195^, Marcus Dörr ^196,197^, Gunnar Engström ^19^, Ian Ford ^198^, Wander S Gurpreet ^199^, Anders Hamsten ^200,201^, Laura Heitsch ^202^, Atsushi Hozawa ^203^, Laura Ibanez ^204^, Andreea Ilinca ^95,96^, Martin Ingelsson ^205^, Motoki Iwasaki ^206^, Rebecca D Jackson ^207^, Katarina Jood ^208^, Pekka Jousilahti ^63^, Sara Kaffashian ^4,5^, Lalit Kalra ^209^, Masahiro Kamouchi ^210^, Takanari Kitazono ^211^, Olafur Kjartansson ^212^, Manja Kloss ^213^, Peter J Koudstaal ^214^, Jerzy Krupinski ^215^, Daniel L Labovitz ^216^, Cathy C Laurie ^118^, Christopher R Levi ^217^, Linxin Li ^218^, Lars Lind ^219^, Cecilia M Lindgren ^220,221^, Vasileios Lioutas ^222,48^, Yong Mei Liu ^223^, Oscar L Lopez ^224^, Hirata Makoto ^225^, Nicolas Martinez-Majander ^172^, Koichi Matsuda ^225^, Naoko Minegishi ^203^, Joan Montaner ^226^, Andrew P Morris ^227,228^, Elena Muiño ^31^, Martina Müller-Nurasyid ^229,230,231^, Bo Norrving ^95,96^, Soichi Ogishima ^203^, Eugenio A Parati ^232^, Leema Reddy Peddareddygari ^56^, Nancy L Pedersen ^98,233^, Joanna Pera ^129^, Markus Perola ^63,234^, Alessandro Pezzini ^235^, Silvana Pileggi ^236^, Raquel Rabionet ^237^, Iolanda Riba-Llena ^30^, Marta Ribasés ^238^, Jose R Romero ^185,48^, Jaume Roquer ^239,240^, Anthony G Rudd ^241,242^, Antti-Pekka Sarin ^243,244^, Ralhan Sarju ^199^, Chloe Sarnowski ^47,48^, Makoto Sasaki ^245^, Claudia L Satizabal ^185,48^, Mamoru Satoh ^245^, Naveed Sattar ^246^, Norie Sawada ^206^, Gerli Sibolt ^172^, Ásgeir Sigurdsson ^247^, Albert Smith ^248^, Kenji Sobue ^245^, Carolina Soriano-Tárraga ^240^, Tara Stanne ^249^, O Colin Stine ^250^, David J Stott ^251^, Konstantin Strauch ^229,252^, Takako Takai ^203^, Hideo Tanaka ^253,254^, Kozo Tanno ^245^, Alexander Teumer ^255^, Liisa Tomppo ^172^, Nuria P Torres-Aguila ^31^, Emmanuel Touze ^256,257^, Shoichiro Tsugane ^206^, Andre G Uitterlinden ^258^, Einar M Valdimarsson ^259^, Sven J van der Lee ^16^, Henry Völzke ^255^, Kenji Wakai ^253^, David Weir ^260^, Stephen R Williams ^261^, Charles DA Wolfe ^241,242^, Quenna Wong ^118^, Huichun Xu ^191^, Taiki Yamaji ^206^, Dharambir K Sanghera ^125,169,170^, Olle Melander ^19^, Christina Jern ^171^, Daniel Strbian ^172,173^, Israel Fernandez-Cadenas ^31,30^, W T Longstreth, Jr ^174,65^, Arndt Rolfs ^175^, Jun Hata ^107^, Daniel Woo ^82^, Jonathan Rosand ^12,13,14^, Guillaume Pare ^15^, Jemma C Hopewell ^176^, Danish Saleheen ^177^, Kari Stefansson ^11,178^, Bradford B Worrall ^179^, Steven J Kittner ^37^, Sudha Seshadri ^180,48^, Myriam Fornage ^74,181^, Hugh S Markus ^3^, Joanna MM Howson ^28^, Yoichiro Kamatani ^6,182^, Stephanie Debette ^4,5^, Martin Dichgans ^1,183,184^
